# Antennal Transcriptome Analysis of Odorant Reception Genes in the Red Turpentine Beetle (RTB), *Dendroctonus valens*

**DOI:** 10.1371/journal.pone.0125159

**Published:** 2015-05-04

**Authors:** Xiao-Cui Gu, Ya-Nan Zhang, Ke Kang, Shuang-Lin Dong, Long-Wa Zhang

**Affiliations:** 1 Anhui Provincial Key Laboratory of Microbial Control, School of Forestry & Landscape Architecture, Anhui Agricultural University, Hefei, China; 2 Education Ministry, Key Laboratory of Integrated Management of Crop Diseases and Pests, College of Plant Protection, Nanjing Agricultural University, Nanjing, China; 3 College of Life Sciences, Huaibei Normal University, Huaibei, China; United States Department of Agriculture, Beltsville Agricultural Research Center, UNITED STATES

## Abstract

**Background:**

The red turpentine beetle (RTB), *Dendroctonus valens* LeConte (Coleoptera: Curculionidae, Scolytinae), is a destructive invasive pest of conifers which has become the second most important forest pest nationwide in China. *Dendroctonus valens* is known to use host odors and aggregation pheromones, as well as non-host volatiles, in host location and mass-attack modulation, and thus antennal olfaction is of the utmost importance for the beetles’ survival and fitness. However, information on the genes underlying olfaction has been lacking in *D*. *valens*. Here, we report the antennal transcriptome of *D*. *valens* from next-generation sequencing, with the goal of identifying the olfaction gene repertoire that is involved in *D*. *valens* odor-processing.

**Results:**

We obtained 51 million reads that were assembled into 61,889 genes, including 39,831 contigs and 22,058 unigenes. In total, we identified 68 novel putative odorant reception genes, including 21 transcripts encoding for putative odorant binding proteins (OBP), six chemosensory proteins (CSP), four sensory neuron membrane proteins (SNMP), 22 odorant receptors (OR), four gustatory receptors (GR), three ionotropic receptors (IR), and eight ionotropic glutamate receptors. We also identified 155 odorant/xenobiotic degradation enzymes from the antennal transcriptome, putatively identified to be involved in olfaction processes including cytochrome P450s, glutathione-*S*-transferases, and aldehyde dehydrogenase. Predicted protein sequences were compared with counterparts in *Tribolium castaneum*, *Megacyllene caryae*, *Ips typographus*, *Dendroctonus ponderosae*, and *Agrilus planipennis*.

**Conclusion:**

The antennal transcriptome described here represents the first study of the repertoire of odor processing genes in *D*. *valens*. The genes reported here provide a significant addition to the pool of identified olfactory genes in Coleoptera, which might represent novel targets for insect management. The results from our study also will assist with evolutionary analyses of coleopteran olfaction.

## Introduction

A sophisticated olfactory system is crucial to insects for survival and reproduction [1,2]. Antennae are the primary olfactory sensors of insects, where odorant messages (such as host volatiles, pheromones, or non-host volatiles) are perceived and subsequently translated into physiological signals that ultimately influence the insect’s behavior [3]. Antennae are accordingly well-equipped with a wide variety of sensilla. These sensilla are small sensory hair structures in which olfactory receptor neurons (ORNs) extend dendrites into the antennal lymph, where peripheral olfactory signal transduction events occur [4]. The ORNs act as biological transducers that convert ecologically relevant volatile signals into a sensory input [3]. The entire olfactory system is heavily dependent on the types of receptors expressed on peripheral ORNs [2]. The ability of the insect’s peripheral system to selectively detect and rapidly inactivate minute amounts of odorants once they have conveyed information is the cornerstone of a sophisticated olfactory system [2]. Diverse peripheral olfactory proteins have been reported to have roles in olfaction. These include the odorant binding proteins (OBPs), chemosensory proteins (CSPs), gustatory receptors (GRs), olfactory receptor proteins (ORs), sensory neuron membrane proteins (SNMPs), and ionotropic receptors (IRs), all of which are involved in different steps in the insect olfactory signal transduction pathway [4].

OBPs are small, hydrophilic proteins that are secreted by the accessory cells and accumulate in the sensillar lymph [2,3]. The soluble OBPs facilitate the transport of odorant molecules through the sensillar lymph and are the liaison between the external environment and Ors [2,4]. CSPs, like OBPs, are another class of small soluble proteins that are expressed at high levels in the sensillar lymph [3,5]. The exact role of CSPs in olfactory transduction remains largely unknown. However, the binding affinity of some CSPs for pheromone molecules supports their putative role in insect olfaction [6–9].

ORs have seven transmembrane domains with inverted membrane topology [10,11] and are embedded in the dendrites of ORNs. ORs are the key players in chemosensory signal transduction processes [4]. Two ORs are required in order to transduce odor-evoked signals, an olfactory receptor coreceptor (Orco) [12,13] and a specific OR, which varies according to ORN type [14]. Orco is both highly conserved across insect orders and widely expressed in the majority of ORNs, acting as an ion channel [15,16]. The individual ORs are associated with odorant-binding specificity [17]. ORs respond to a variety of volatile chemicals, including pheromones, and plant- and microbe-derived compounds [16,18,19].

IRs are relatives of ionotropic glutamate receptors (IGluRs) with atypical binding domains that are conserved across proteosome lineages; IRs are far more ancient than ORs [20]. IRs were recently discovered as another class of insect-specific gene products that are involved in odorant recognition in *Drosophila* [3]. The IR family contains a conserved subgroup, the antennal IRs, and a species-specific subgroup, the divergent IRs [20]. The antennal IRs are a novel group of chemosensory receptors and are expressed in sensory dendrites [4].

SNMPs were first found in the dendritic membrane of sex pheromone-sensing olfactory neurons (ORNs) of the wild silk moth, *Antheraea polyphemus* (Saturniidae), [21] and are proposed to play an important role in pheromone reception in insects [22–25]. SNMPs are homologous to the mammalian CD36 protein and have been shown to be required for detecting an aggregation pheromone in *Drosophila* [22]. SNMP1 and SNMP2 genes has been found in several different coleopteran insects [5,26]. Insects express SNMP1 and SNMP2 in pheromone-sensitive hairs, but in different locations: SNMP1 is specifically expressed in the dendritic membrane of a neuron of an olfactory sensillum, whereas SNMP2 is only found in supporting cells or the sensillar lymph of the antenna [27–29].

The red turpentine beetle (RTB), *Dendroctonus valens* LeConte (Coleoptera: Curculionidae: Scolytinae), is a secondary pest of pines in its native range in North and Central America. *Dendroctonus valens* was introduced into China in the early 1980s, where it aggressively kills pine species native to China [30–32]. The behavior of *D*. *valens* in its native range is clearly different from that in China [31]. Since the outbreaks of *D*. *valens* in 1999, it has infected over 5,000,000 ha of pine forest, and more than 10 million *Pinus tabuliformis* Carr. as well as other pine species such as *Pinus bungeana* Zucc [31,33–35] and has resulted in unprecedented economic losses. At present, *D*. *valens* is the second most important forest pest nationwide in China [31] and its spread continues. As with most insect species, antennal olfaction is of the utmost importance in *D*. *valens* fitness because the beetle uses aggregation pheromones, as well as host and non-host volatiles in intra- and inter-specific communication [36–50]. However, there is little information on the molecular mechanisms underlying olfaction in *D*. *valens*. Investigating the repertoire of odor processing genes involved in *D*. *valens* olfaction could provide valuable insights into the molecular mechanisms of insect olfaction, and also identify possible molecular targets that could be manipulated for *D*. *valens* control.

Although the genome of *Tribolium castaneum* has been sequenced, our knowledge of the molecular basis of odorant reception in Coleoptera, the largest insect order, remains relatively limited. Recently, the advent of RNA-Seq approaches (next generation sequencing techniques) triggered an exponential growth in our knowledge of insect olfaction. Genes involved in odor processing in Coleoptera have been identified from insects whose genomes have not been sequenced, such as *Ips typographus*, *Dendroctonus ponderosae* [5], and *Agrilus planipennis* [26]. Additional beetle species need to be investigated to reach a better understanding of the molecular biology of coleopteran and insect olfaction.

Here, we employed a transcriptome approach based on next-generation sequencing of antennae of *D*. *valens* to identify the olfactory gene repertoire involved in *D*. *valens’* odor-processing. In this study, we conducted a transcriptome analysis of antennae of adult beetles, and identified 68 putative chemosensory transcripts comprising 21 OBPs, six CSPs, four SNMPs, 22 ORs, four GRs, three IRs, and eight ionotropic glutamate receptors. We also obtained 155 odorant/xenobiotic degradation enzymes from the antennal transcriptome, putatively identified to be involved in olfaction processes including cytochrome P450s, glutathione-S-transferases, and aldehyde dehydrogenase.

## Results

### Transcriptome Sequencing and Assembly

**Fig 1:**
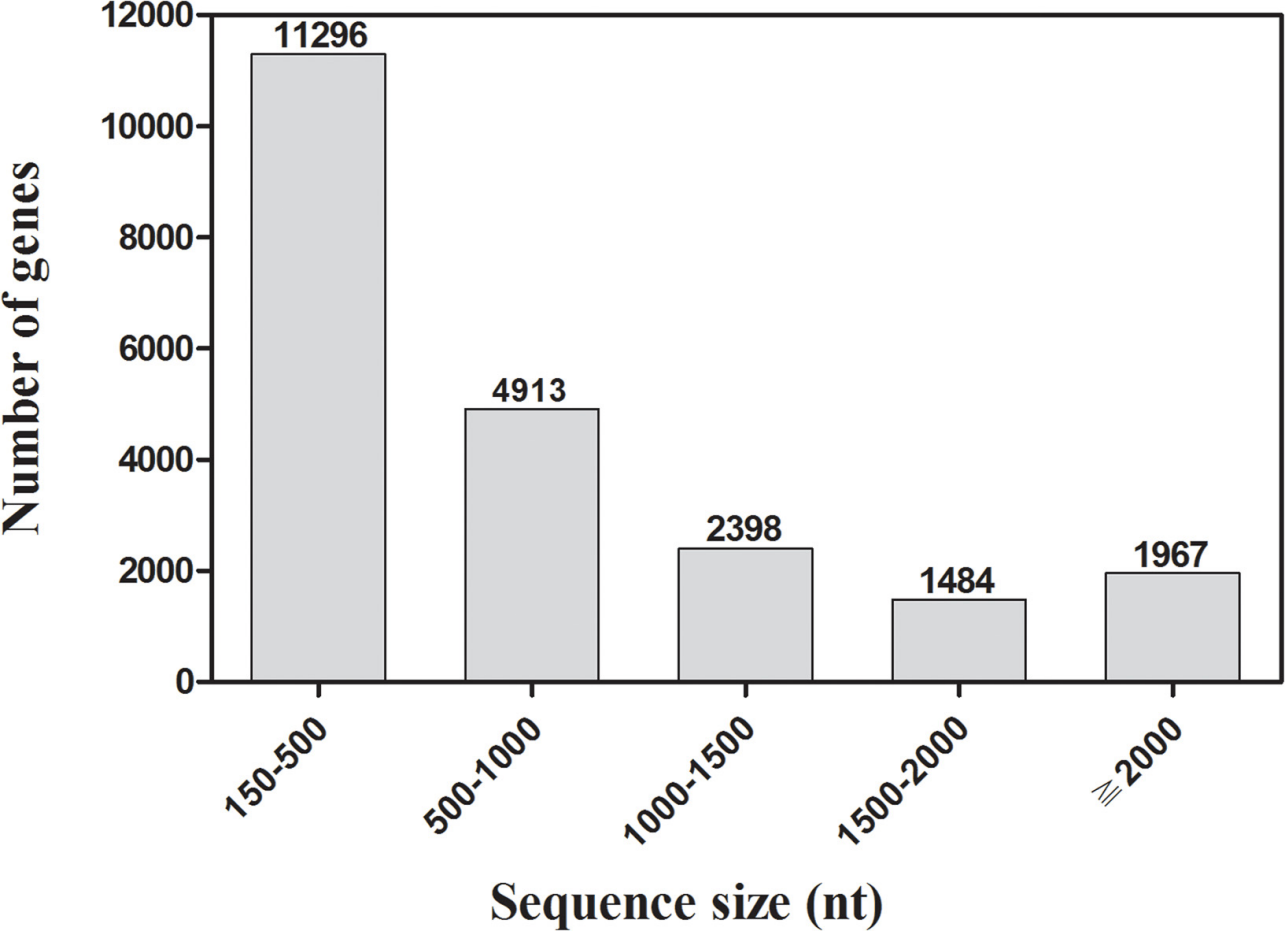
Distribution of unigene size in the *D*. *valens* transcriptome assembly.

A total of approximately 51 million reads (4.86 Gb) were obtained from a *D*. *valens* antennaapp:addword:antenna sample by the Illumina HiSeq 2000 platform, which were assembled into 39,831 contigs with a mean length of 409 bp and an N50 length of 814 bp. After clustering and redundancy filtering, we finally acquired 22,058 unigenes (4,281 clusters and 17,777 singletons) with a mean length of 828 bp and a N50 length of 1360 bp. Of the 22,058 unigenes, those with a sequence length of more than 500 bp accounted for 48.79% of the transcriptome assembly (Fig 1). All the unigenes are referred to as transcripts hereafter and given a unique unigene ID.

### Homology Analysis and Gene Ontology (GO) Annotation

**Fig 2:**
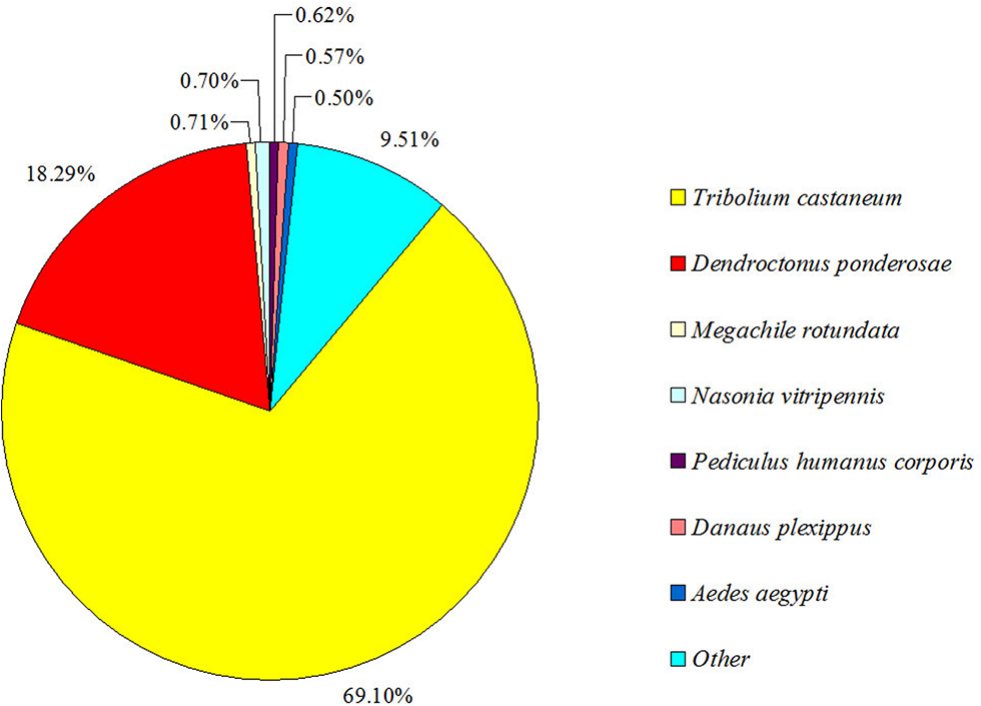
Percentage of homologous hits of the *D*. *valens* transcripts to other insect species. The *D*. *valens* transcripts were searched by BlastX against the non-redundancy protein database with a cutoff E-value of 10^−5^. Species which have more than 0.5% matching hits to the *D*. *valens* transcripts are shown.

Among 22,058 transcripts, 15,531 (70.40%) were matched by the BlastX homology search to entries in the NCBI non-redundant (nr) protein database with a cut-off E-value of 10^−5^. The species distribution of the best match result for each sequence is shown in Fig 2. Overall, there is a strong match preference with *T*. *castaneum* genes, comprising 69.10%. Interestingly, there was only an 18.29% similarity of *D*. *valens* antennal transcripts to those of the congener *D*. *ponderosae*. However, this result does not mean that the identified transcripts in *D*. *valens* share more similarity with *T*. *castaneum* transcripts than *D*. *ponderosae* transcripts. Instead, it is likely due to the availability of more *T*. *castaneum* than *D*. *ponderosae* sequences in the NCBI protein database used in the homology analysis. The protein number of *T*. *castaneum* in the NCBI database is 36,478, 1.2 times higher than that of *D*. *ponderosae* (29,316). With more protein sequences annotated from *D*. *ponderosae*, we believe that more sequences in *D*. *valens* will align to those of *D*. *ponderosae*.

**Fig 3:**
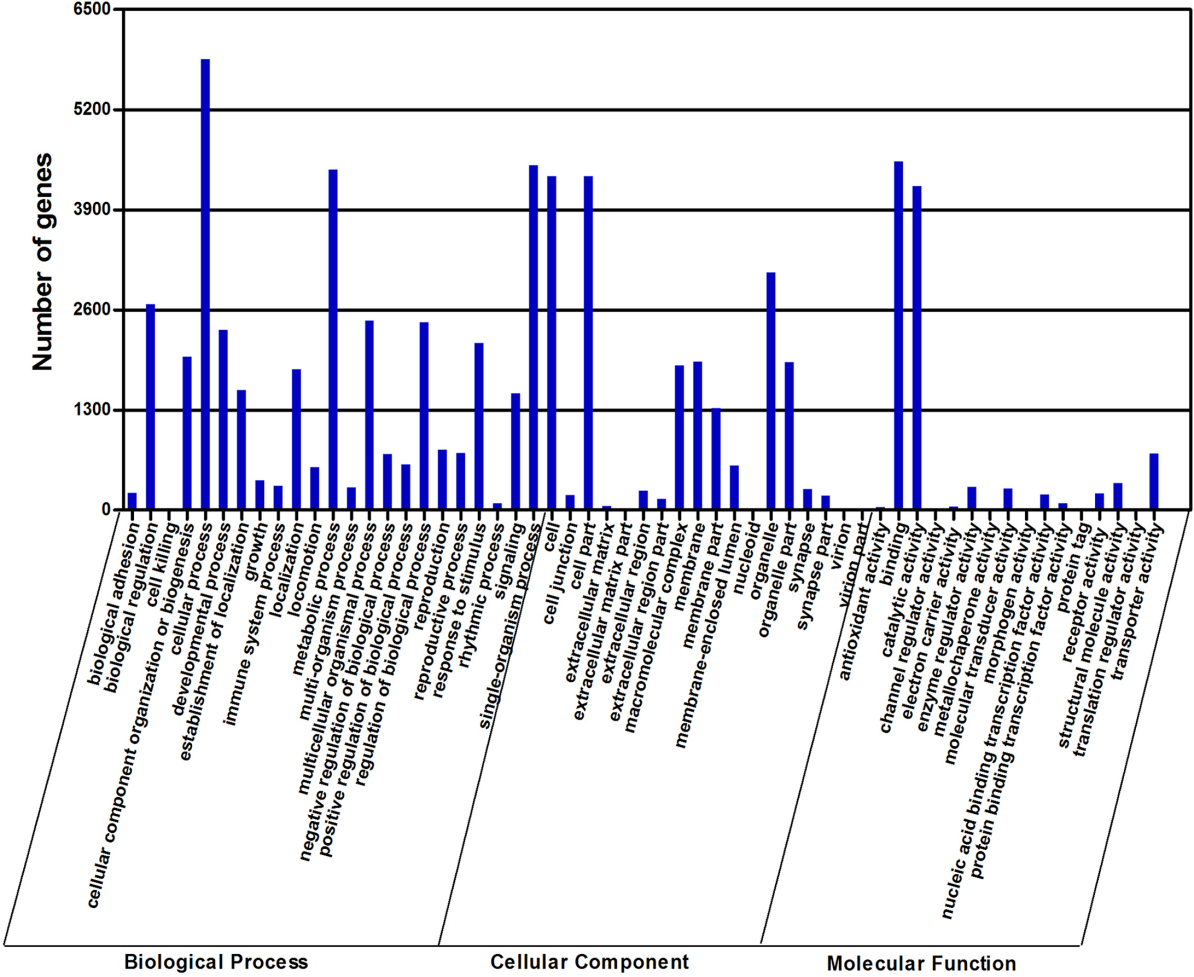
Gene ontology (GO) classification of the *D*. *valens* transcripts with Blast2GO program.

The gene functional annotation was first performed by GO annotation using Blast2GO. Of 22,058 transcripts, 8,743 (39.64%) could be annotated based on sequence homology. Because one transcript could align to more than one biological process, the 8,743 transcripts were assigned to the biological process category (38,416 alignments), cellular component category (20,508 alignments), and molecular function category (11,016 alignments). The major GO terms associated with molecular function were binding (41.09%) and catalytic activity (38.19%), which potentially reflects the metabolic processes of the antennal tissue (Fig 3). Cellular processes (15.23%), single-organism processes (11.66%), and metabolic processes (11.50%) were the main subcategories of biological processes, indicative of the important metabolic activities within *D*. *valens* antennae (Fig 3 and S1 Table). Under the category of cellular components, cell (21.11%) and cell parts (21.11%) were among the most highly represented subcategories (Fig 3 and S1 Table).

### Identification of Putative Olfactory Genes

**Fig 4:**
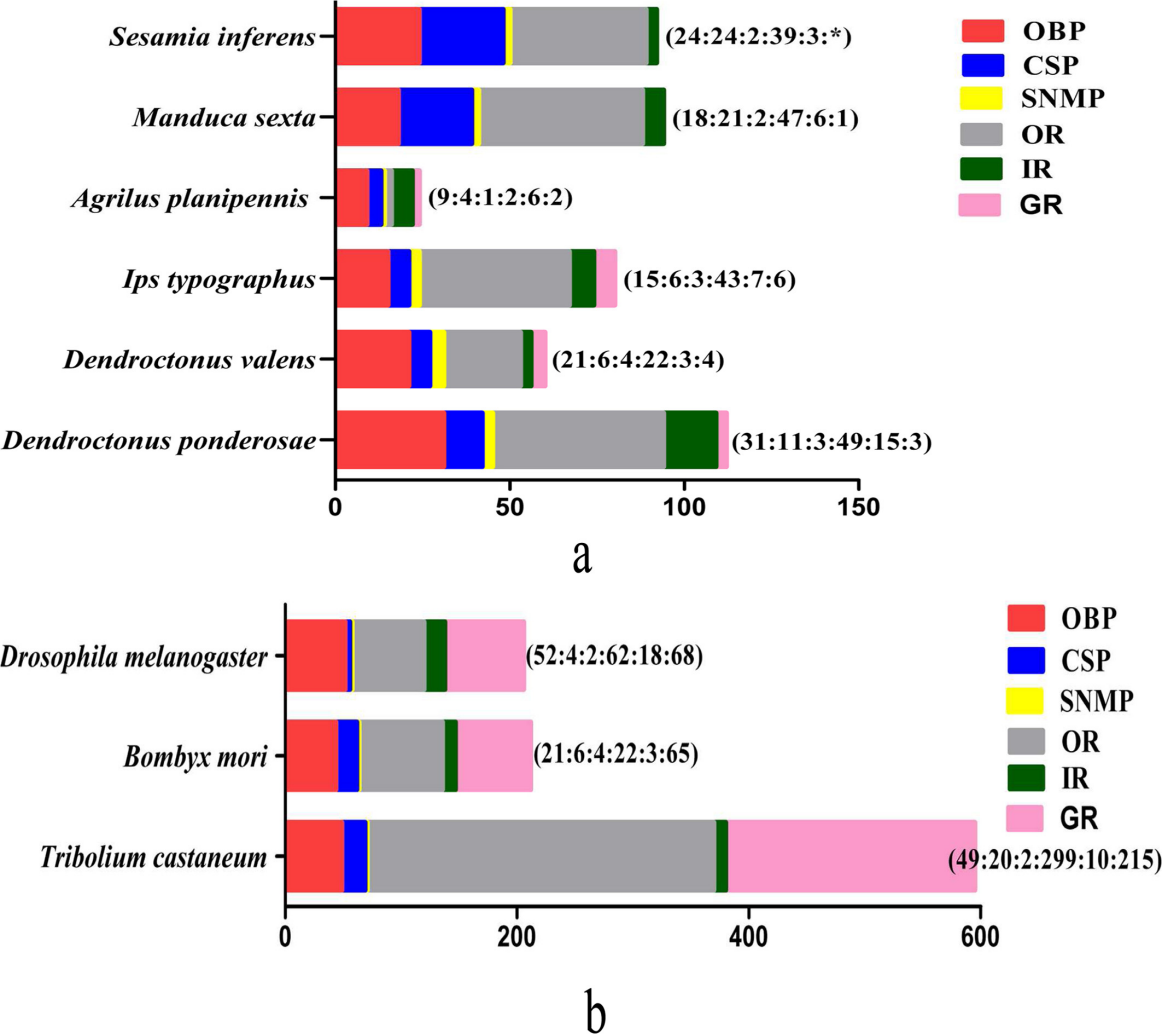
The number of chemosensory genes in different insect species, obtained from antenna transcriptome (4a) or genome (4b). The digits by the histogram bars represent number of chemosensory genes in different subfamilies (OBP:CSP:SNMP:OR:IR). The data were obtained from the current study for *D*. *valens* and from references [7,20,24,91] for *Drosophila melanogaster*, [92] for *Sesamia inferens*,[20,24,62,93–95] for *Bomby mori*, [7,20,24,91] for *Tribolium castaneum*,[5] for *I*. *typographus* and *D*. *ponderosae*, [1] for *Manduca sexta*, [26] for *Agrilus planipennis*.

Further analysis on the transcriptome of *D*. *valens* focused on gene families associated with olfactory processing. By homology analysis, we identified 68 putative odor-reception genes, including 21 OBPs, six CSPs, four SNMPs, 22 ORs, four GRs, three IRs, and eight IGluRs. As well, 155 putative odorant/xenobiotic degradation enzymes were found (Table 1). The number of major olfactory genes obtained in the current study was larger than that of *A*. *planipennis* [26], but less than that of *D*. *ponderosae* and *I*. *typographus* (Fig 4A and 4B).

**Table 1:** Summary of candidate genes from the antennal transcriptome of *Dendroctonus valens*.

Candidate genes	# in occurrence
Odor-reception	
Odor binding proteins	21
Odorant receptors	22
Ionotropic receptor	3
Ionotropic glutamate receptors	8
Gustatory receptors	4
Sensory neuron membrane proteins	4
Chemsensory proteins	6
Odor/xenobiotic degradation	
Cytochrome P450s	49
Glutathione S-transferases	11
Esterases	66
Aldehyde dehydrogenases	14
Epoxide hydrolases	4
Catalases	3
Superoxide dismutase	5
Glutathione peroxidase	3

### Identification of Putative Odorant-binding Proteins

**Fig 5:**
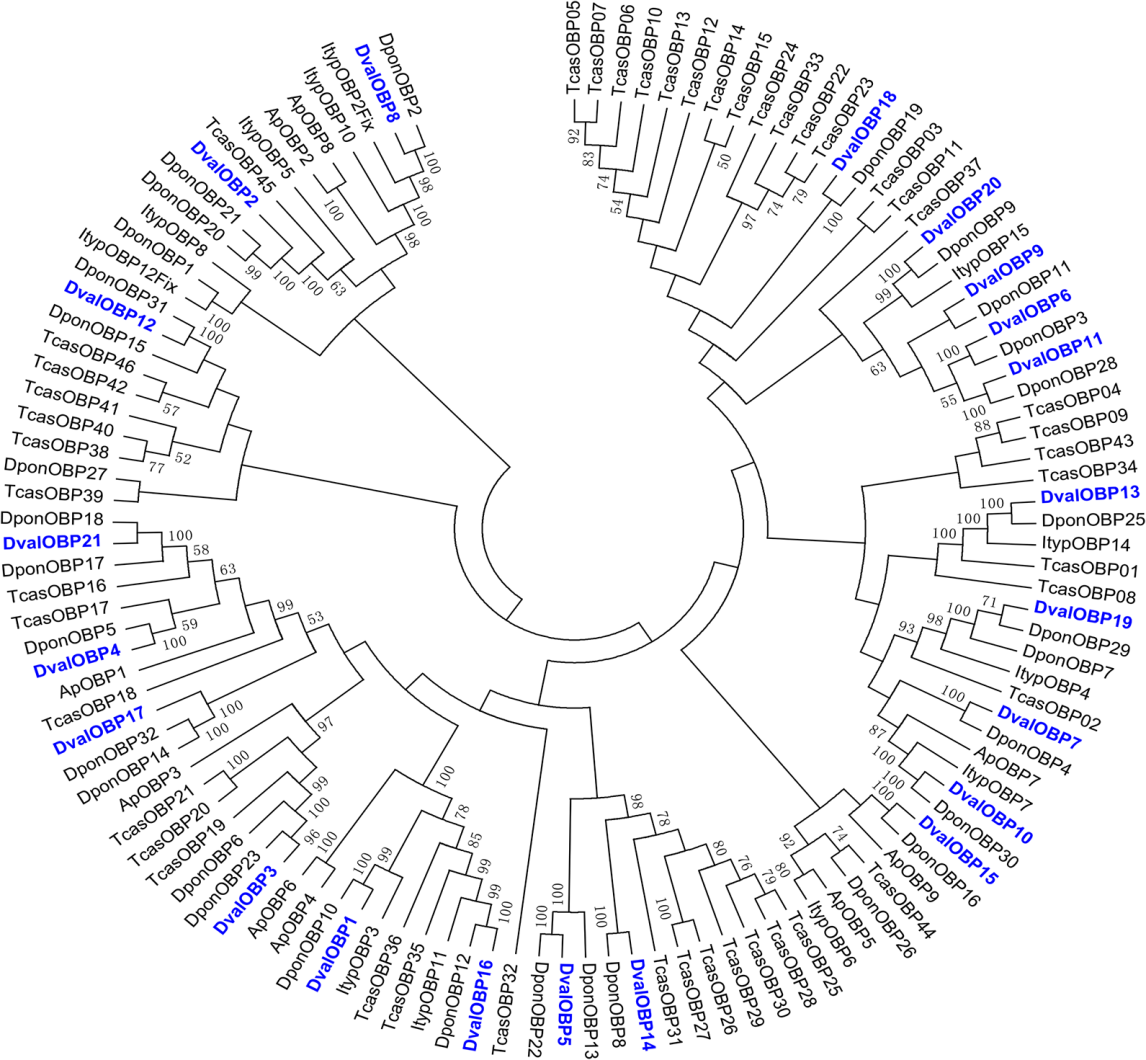
Phylogenetic tree of putative OBPs from *Dendroctonus valens* (*Dval*), *Ips typographus (Ityp)*, *Dendroctonus ponderosae* (*Dpon*), *Tribolium castaneum* (*Tcas*) and *Agrilus planipennis* (*Ap*). The *D*. *valens* translated unigenes are shown in blue. Amino acid sequences are given in S1 Fig. The tree was constructed with MEGA5.0, using the neighbor-joining method. Values indicated at the nodes are bootstrap values based on 1000 replicates, and the bootstrap values below 50% are not shown.

We identified 21 transcripts encoding putative OBPs in the *D*. *valens* antennal transcriptome. The number of putative OBP-coding genes in *D*. *valens* is less than that of *D*. *ponderosae* (31), but more than *I*. *typographus* (15). Sequence analysis identified 18 unigenes with a full length ORF (Open Reading Frame) with predicted signal peptide sequences (Table 2). Twenty of the 21 putative OBPs had high similarity to known coleopteran OBPs, (Table 2). DvalOBP3, 4, 7, 10, 14, 16, 18, and 21 had very high similarity with DponOBP6, 5, 4, 30, 8, 12, 19, and 18, respectively, with at least 95% identity (Table 2). A phylogenetic tree based on the neighbor-joining method is shown in Fig 5. Among the 21 putative OBPs, 19 sequences were clustered with at least one coleopteran orthologous gene (Fig 5). Not surprisingly, the identified DvalOBPs sequences were mostly clustered together with DponOBPs, and fourteen OBP orthologous pairs shared high amino acid identity (>85%) between the two species (Fig 5 and Table 2). The amino acid sequences of all OBPs are listed in S1 Fig.

**Table 2:** The Blastx match of *D*. *valens* putative OBPs,CSPs and SNMPs genes.

Gene	Gene	ORF	Complete	Signal	Best Blastx Match				
Name	ID	Length(bp)	ORF	Peptide	Name	Acc.number	Species	E value	Identity(%)
Odorant Binding Protein(OBP)									
OBP1	455	438	Yes	1–23	odorant-binding protein 10	AFI45063.1	[*Dendroctonus ponderosae*]	6.00E-95	91
OBP2	840	726	Yes	1–19	odorant-binding protein 21	AGI05159.1	[*Dendroctonus ponderosae*]	1.00E-120	87
OBP3	849	417	Yes	1–20	odorant-binding protein 6	AGI05177.1	[*Dendroctonus ponderosae*]	1.00E-75	95
OBP4	10282	408	Yes	1–19	odorant-binding protein 5	AFI45059.1	[*Dendroctonus ponderosae*]	2.00E-72	95
OBP5	10284	405	Yes	1–23	odorant-binding protein 22	AGI05180.1	[*Dendroctonus ponderosae*]	5.00E-79	93
OBP6	1456	426	Yes	1–20	odorant-binding protein 3	AGI05174.1	[*Dendroctonus ponderosae*]	7.00E-85	82
OBP7	14597	222	No	0	odorant-binding protein 4	AGI05167.1	[*Dendroctonus ponderosae*]	3.00E-52	99
OBP8	5689	522	No	0	odorant-binding protein 2	AGI05158.1	[*Dendroctonus ponderosae*]	2.00E-109	94
OBP9	5818	395	Yes	1–17	odorant-binding protein 28	AGI05178.1	[*Dendroctonus ponderosae*]	3.00E-10	32
OBP10	7393	402	Yes	1–18	odorant-binding protein 30	AGI05176.1	[*Dendroctonus ponderosae*]	4.00E-77	98
OBP11	7769	507	No	0	odorant-binding protein 28	AGI05178.1	[*Dendroctonus ponderosae*]	9.00E-63	93
OBP12	8450	387	Yes	1–24	odorant-binding protein 31	AGI05165.1	[*Dendroctonus ponderosae*]	3.00E-67	93
OBP13	8460	447	Yes	1–19	odorant binding protein	ADY17884.1	[*Spodoptera exigua*]	1.00E-16	46
OBP14	9456	475	Yes	1–27	odorant-binding protein 8	AGI05175.1	[*Dendroctonus ponderosae*]	1.00E-120	96
OBP15	9499	354	Yes	1–17	odorant-binding protein 16	AGI05186.1	[*Dendroctonus ponderosae*]	7.00E-72	91
OBP16	9616	426	Yes	1–18	odorant-binding protein 12	AFI45058.1	[*Dendroctonus ponderosae*]	1.00E-93	95
OBP17	9643	435	Yes	1–21	odorant-binding protein 13	AFI45057.1	[*Dendroctonus ponderosae*]	9.00E-83	88
OBP18	9644	426	Yes	1–18	odorant-binding protein 19	AGI05183.1	[*Dendroctonus ponderosae*]	1.00E-65	95
OBP19	9906	411	Yes	1–19	odorant-binding protein 29	AGI05182.1	[*Dendroctonus ponderosae*]	3.00E-61	94
OBP20	9965	423	Yes	1–20	odorant-binding protein 9	AGI05185.1	[*Dendroctonus ponderosae*]	2.00E-80	90
OBP21	9974	408	Yes	1–19	odorant-binding protein 18	AFI45062.1	[*Dendroctonus ponderosae*]	2.00E-71	95
Chemosensory Protein(CSP)									
CSP1	10610	432	Yes	1–26	chemosensory protein5	AFI45003.1	[*Dendroctonus ponderosae*]	2.00E-81	94
CSP2	8762	375	Yes	1–16	chemosensory protein 1	AGI05161.1	[*Dendroctonus ponderosae*]	3.00E-54	90
CSP3	3805	102	No	0	chemosensory protein 2	AGI05172.1	[*Dendroctonus ponderosae*]	5.00E-33	89
CSP4	168	822	Yes	1–18	chemosensory protein 6 precursor	NP_001039288.1	[*Tribolium castaneum*]	1.00E-49	67
CSP5	8684	423	No	0	chemosensory protein 8	AGI05164.1	[*Dendroctonus ponderosae*]	2.00E-82	96
CSP6	11692	309	Yes	1–23	chemosensory protein 11	AGI05163.1	[*Dendroctonus ponderosae*]	3.00E-55	94
Sensory Neuron Membrane Protein(SNMP)									
SNMP1	561	954	No	No	sensory neuron membrane protein1	AFI45066.1	[*Dendroctonus ponderosae*]	0	94
SNMP1a	10985	1674	No	No	sensory neuron membrane protein 1a	AGI05171.1	[*Dendroctonus ponderosae*]	0	95
SNMP2	7572	1338	No	No	sensory neuron membrane protein 2, isoform B	NP_001036593.1	[*Drosophila melanogaster*]	2.00E-83	35
SNMP	11087	225	No	No	sensory neuron membrane protein	AFI45067.1	[*Dendroctonus ponderosae*]	4.00E-39	85

**Fig 6:**
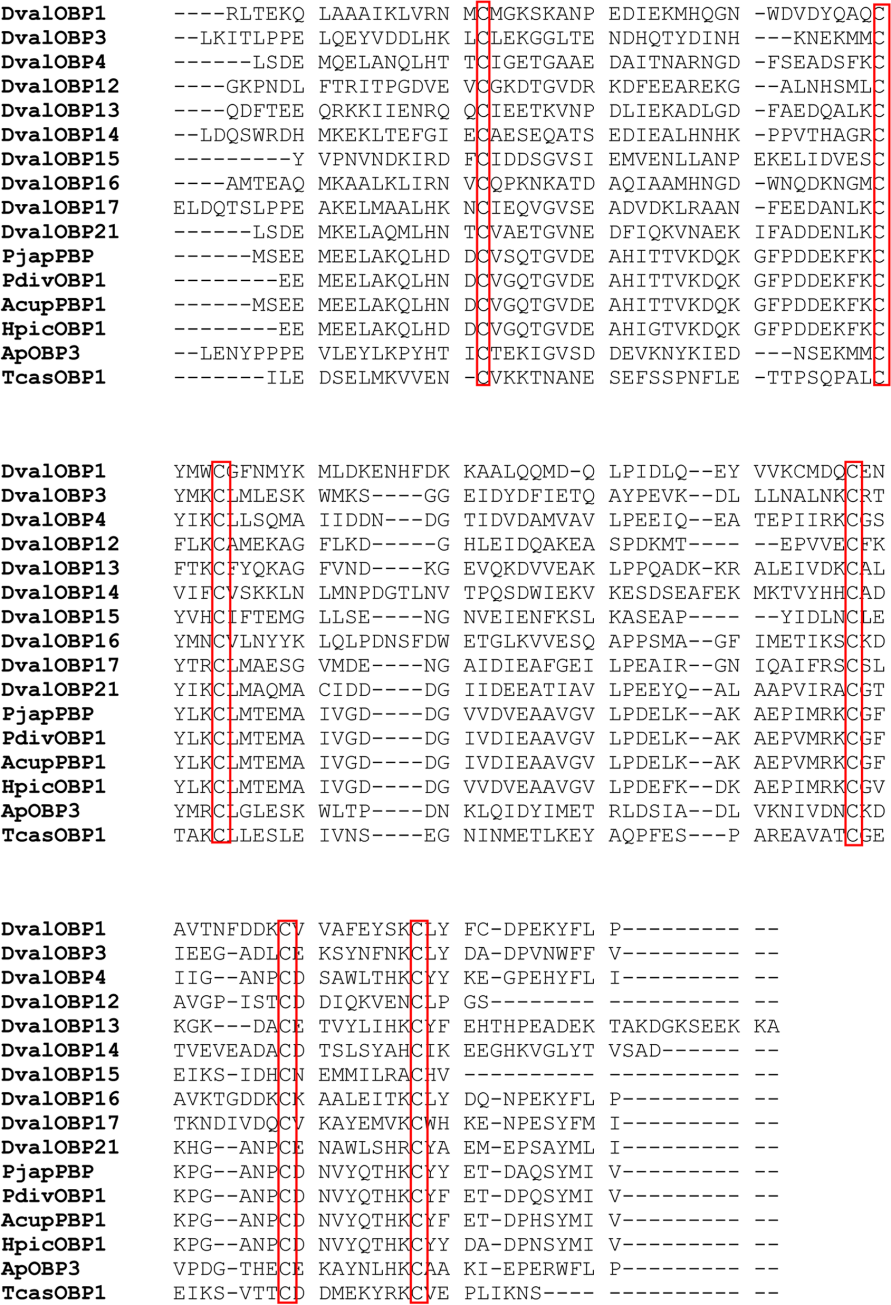
Multiple sequences alignment of OBPs of *Dendroctonus valens* (*Dval*) with other insects OBPs. Analyses included OBPs and pheromone binding proteins (PBPs). Amino acid sequences are given in S2 Fig.

In general, OBPs are divided into different subclasses. The classic OBPs are characterized by a pattern of six conserved cysteines. Multiple amino acid sequence alignment revealed the conserved and defined spacing of six cysteine residues (C1-X15-39-C2-X3-C3-X21-44-C4-X7-12-C5-X8-C6; Fig 6), which are considered to form disulfide bonds that stabilize the three-dimensional structure of the OBP [51].

The amino acid sequences used for multiple sequence alignment are listed in S2 Fig. The Plus-C class has more than 6 cysteines, whereas the Minus-C class has lost cysteine residues, generally C2 and C5. In our study, the members of the Minus-C class were DvalOBP2, 5, 6, 9, 10, 18, 19, and 20 and all of these are missing C2 and C5 cysteine residues, whereas only DvalOBP8 was classified as Plus-C.

### Identification of Candidate Chemosensory Proteins

**Fig 7:**
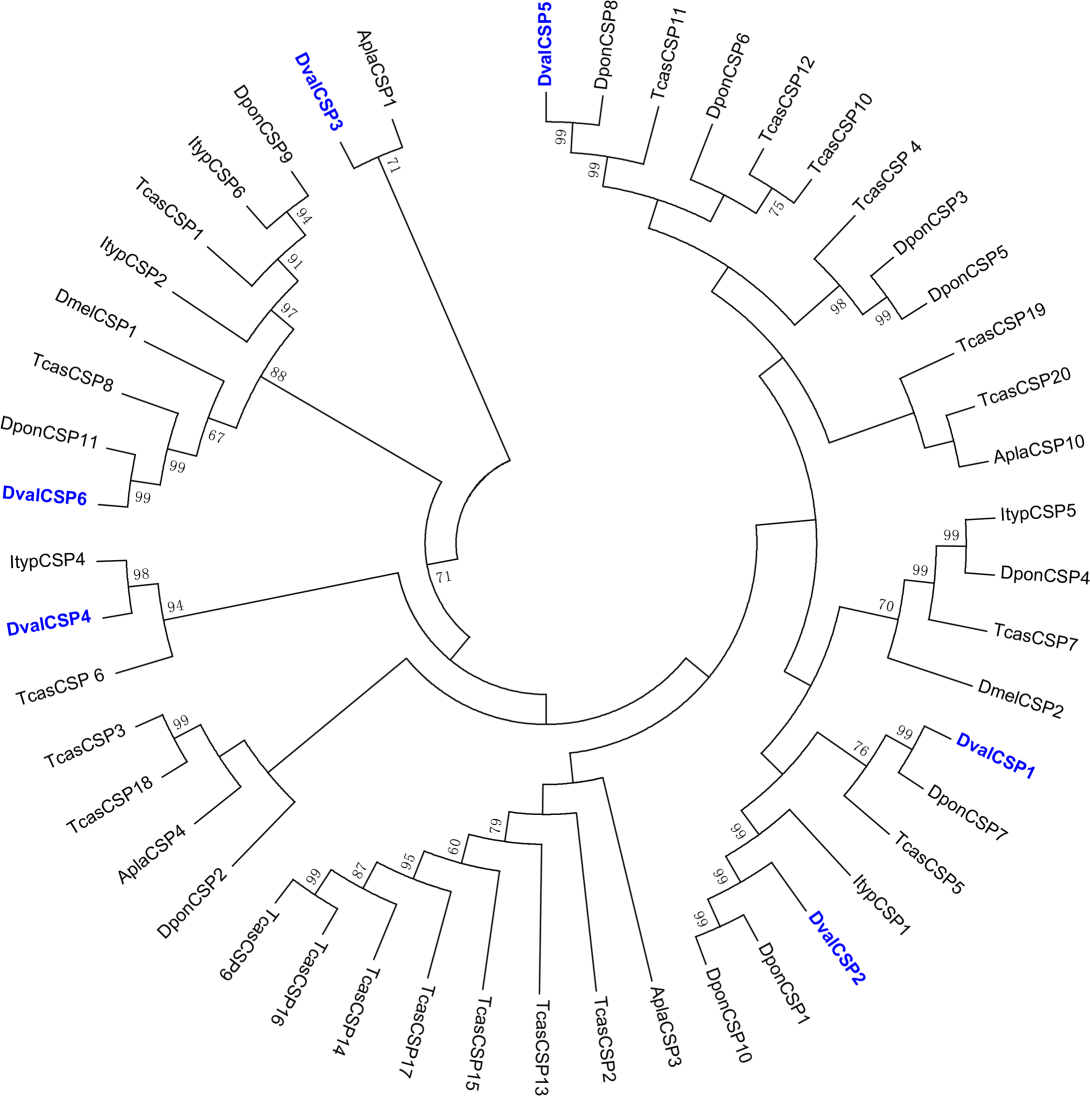
Phylogenetic tree of putative CSPs from *Dendroctonus valens* (*Dval*), *Ips typographus* (*Ityp*), *Dendroctonus ponderosae* (*Dpon*), *Tribolium castaneum* (*Tcas*) and *Drosophila melanogaster* (*Dmel*). The *D*. *valens* translated unigenes are shown in blue. Amino acid sequences are given in S3 Fig. The tree was constructed with MEGA5.0, using the neighbor-joining method. Values indicated at the nodes are bootstrap values based on 1000 replicates, and the bootstrap values below 50% are not shown.

Bioinformatic analysis led to the identification of six different sequences encoding candidate CSPs in *D*. *valens* (Table 2), among which four sequences were predicted to be full length and all of them had a signal peptide. We compared all predicted CSPs of *D*. *valens* with 42 coleopteran CSPs and two dipteran CSPs in order to reveal the diversity of CSPs within the insect order (Fig 7). The CSPs were dispersed in different branches of the phylogenetic tree (Fig 7). Among the six candidate CSPs, five sequences were clustered together with bark beetles CSPs (Fig 7). The information of all the six CSPs is listed in Table 2. DvalCSP1, 5, and 6 had at least 94% similarity with the corresponding CSPs of *D*. *ponderosae* (DponCSP5, 8, and 11; Table 2). The amino acid sequences of all six CSPs are listed in S3 Fig.

### Identification of Putative OR/GR Superfamily Members

Within the *D*. *valens* antennal transcriptome, 22 OR candidates were identified. Among these ORs, only three identified DvalOR sequences (Dval 7, 8 and 11) had a full length ORF (Table 3). As expected, 17 of the 22 putative DvalORs shared high amino acid identity with known coleopteran ORs, and six ORs in *D*. *valens* (DvalORs1, 3, 14, 15, 16, and 17) had at least 87% similarity with corresponding ORs of *D*. *ponderosae* (Table 3).

**Table 3:** The BlastX match of *D*. *valens* putative GRs, IRs and ORs genes.

Gene	Gene	ORF	Complete	Signal	Best BlastX Match				
Name	ID	Length(bp)	ORF	Peptide	Name	Acc.number	Species	E value	Identity(%)
Gustatory Receptor(GR)									
GR1	14175	396	No	0	gustatory receptor 2	NP_001161916.1	[*Tribolium castaneum*]	2.00E-62	73
GR2	16566	207	No	0	Gustatory receptor 21a, putative	XP_001655150.1	[*Aedes aegypti*]	3.00E-26	74
GR3	2217	375	Yes	1–22	putative gustatory receptor candidate 59	EHJ69979.1	[*Danaus plexippus*]	2.00E-04	74
GR4	6503	576	No	0	gustatory receptor 101	EFA02934.1	[*Tribolium castaneum*]	5.00E-14	30
Ionotropic Receptor(IR)									
IR1	11453	171	No	0	ionotropic receptor 8a	AGI05169.1	[*Dendroctonus ponderosae*]	3.00E-31	87
IR2	12733	264	No	0	putative ionotropic receptor IR75, partial	AFC91756.1	[*Cydia pomonella*]	1.00E-10	36
IR3	67	2367	No	0	ionotropic receptor 8a	AGI05169.1	[*Dendroctonus ponderosae*]	0	97
Odorant Receptor(OR)									
OR1	11271	780	No	0	olfactory receptor	AEE62122.1	[*Dendroctonus ponderosae*]	0	97
OR2	40	795	No	0	odorant receptor 20	AEE63423.1	[*Dendroctonus ponderosae*]	7.00E-173	77
OR3	2158	705	No	0	odorant receptor 18	AEE62488.1	[*Dendroctonus ponderosae*]	1.00E-155	97
OR4	5354	789	No	0	odorant receptor	XP_001651754.1	[*Aedes aegypti*]	2.00E-13	29
OR5	8369	528	No	0	olfactory receptor	XP_001864544.1	[*Culex quinquefasciatus*]	2.00E-17	32
OR6	5	723	No	0	odorant receptor 2	ACH69148.1	[*Anopheles stephensi*]	6.00E-06	27
OR7	13471	279	Yes	1–20	odorant receptor 10	ACH69151.1	[*Anopheles stephensi*]	9.00E-10	41
OR8	2313	300	Yes	1–17	odorant receptor 14	EFA09245.1	[*Tribolium castaneum*]	4.00E-05	42
OR9	12394	273	No	0	odorant receptor 23	AGI05173.1	[*Dendroctonus ponderosae*]	8.00E-10	37
OR10	2425	171	No	0	odorant receptor 23	AGI05173.1	[*Dendroctonus ponderosae*]	7.00E-10	44
OR11	2712	241	Yes	1–19	odorant receptor 23	AGI05173.1	[*Dendroctonus ponderosae*]	5.00E-10	39
OR12	4846	390	No	0	odorant receptor 23	AGI05173.1	[*Dendroctonus ponderosae*]	8.00E-22	40
OR13	5024	179	No	0	odorant receptor 23	AGI05173.1	[*Dendroctonus ponderosae*]	1.00E-12	43
OR14	2032	197	No	0	odorant receptor 10	AEE61404.1	[*Dendroctonus ponderosae*]	1.00E-45	99
OR15	9058	1215	No	0	odorant receptor 38	AEE63155.1	[*Dendroctonus ponderosae*]	0.00E+00	92
OR16	9393	378	No	0	odorant receptor 23	AGI05173.1	[*Dendroctonus ponderosae*]	3.00E-61	87
OR17	7310	792	No	0	odorant receptor 24	AGI05166.1	[*Dendroctonus ponderosae*]	4.00E-148	88
OR18	2992	267	No	0	odorant receptor 73	EFA05710.1	[*Tribolium castaneum*]	6.00E-06	35
OR19	1231	195	No	0	odorant receptor 46	EFA02901.1	[*Tribolium castaneum*]	6.00E-11	46
OR20	13762	705	No	0	odorant receptor 59	EEZ99171.1	[*Tribolium castaneum*]	2.00E-10	48
OR21	7442	357	No	0	olfactory receptor 60	NP_001155301.1	[*Bombyx mori*]	9.00E-13	32
OR22	313	798	No	0	odorant receptor 64	EFA10800.1	[*Tribolium castaneum*]	3.00E-112	59

A phylogenetic tree of ORs based on the neighbor-joining method was constructed using protein sequences of ORs from *D*. *valens*, two other bark beetles (*I*. *typographus*, *D*. *ponderosae*), *T*. *castaneum*, *Megacyllene caryae*, and *Agrilus planipennis*. Eleven DvalORs were excluded from analysis due to insufficiently long amino acid sequences.

**Fig 8:**
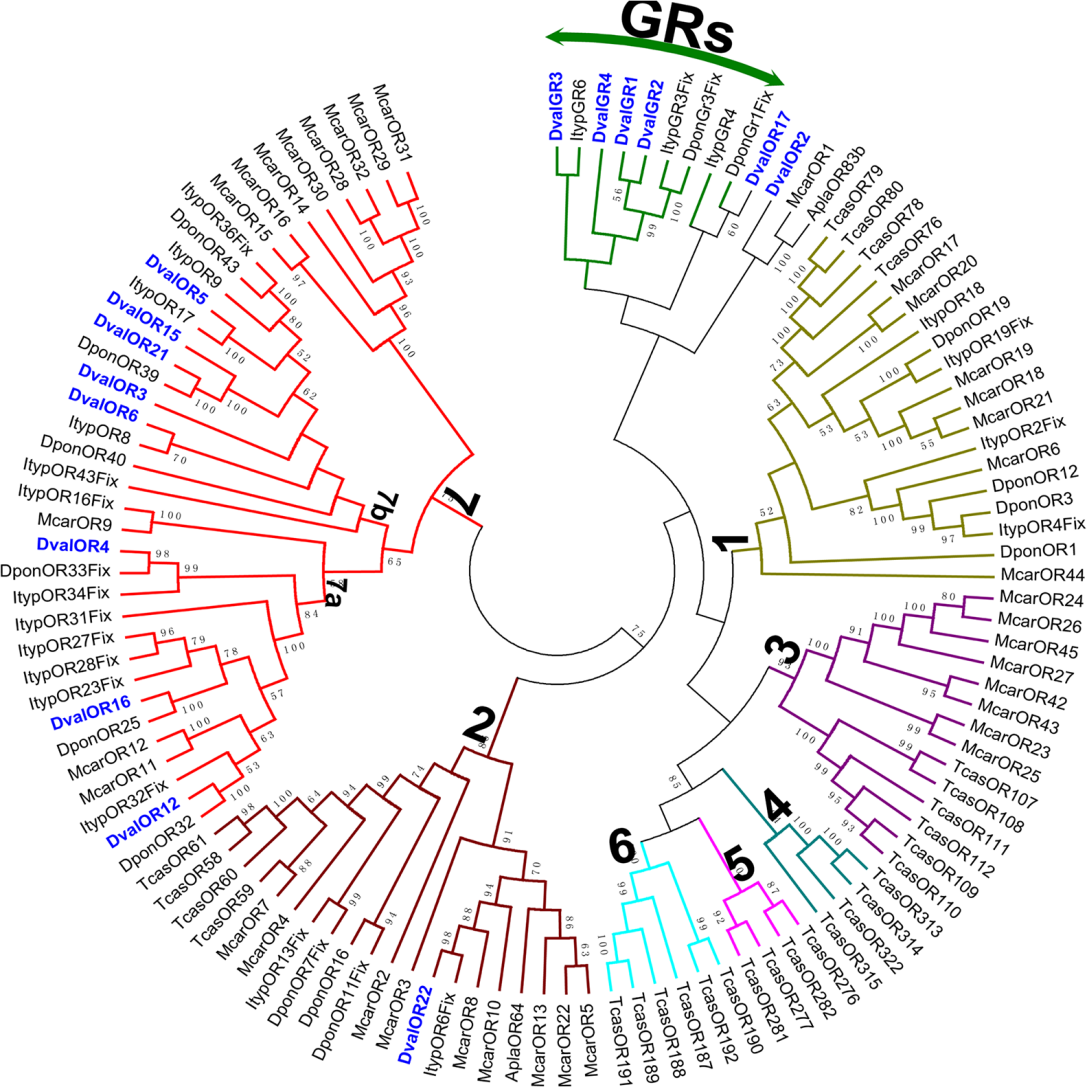
Phylogenetic tree of putative ORs and GRs from *Dendroctonus valens* (*Dval*), *Ips typographus* (*Ityp*), *Dendroctonus ponderosae* (*Dpon*)and *Tribolium castaneum* (*Tcas*). The *D*. *valens* translated unigenes are shown in blue. The branch containing GRs was used as outgroup to root the tree. The different subgroups (numbered 1–7 according to [35,53], and 7a-7b) are discussed in the main text. Originally, DponOR15Fix was found within group 2 [29], as indicated here by the numbers in brackets. Amino acid sequences are given in S4 Fig. The tree was constructed with MEGA5.0, using the neighbor-joining method. Values indicated at the nodes are bootstrap values based on 1000 replicates, and the bootstrap values below 50% are not shown.

ORs are divided into several subgroups of various size and content in the phylogenetic tree (Fig 8). Almost all ORs of *D*. *valens* were clustered with at least one bark beetle orthologous gene in the phylogenetic tree except for two ORs (DvalOR2 and DvalOR17). Among them, five ORs from *D*. *valens* were clustered with one *D*. *ponderosae* orthologous gene.

The numbering of coleopteran OR subfamilies has been standardized in previous studies [5,51], and numbered from 1 to 7. Groups 1 and 2 included OR representatives from all six species. Receptor group 3 contained ORs only from *T*. *castaneum* and *M*. *caryae*. Receptor groups 4–6 contained ORs only from *T*. *castaneum*. Group 7 contained 11 ORs from *M*. *caryae*, but no ORs from *T*. *castaneum* or *A*. *planipennis*. The results showed eight of 11 ORs from *D*. *valens* were present within group 7. These eight DvalORs were further divided into two subgroups: group 7a and 7b. Subgroup 7b was exclusive to bark beetle species, including ORs from *D*. *valens*, *I*. *typographus*, and *D*. *ponderosae*. In three groups (1, 2, and 7), we found most of the candidate genes were grouped together with orthologs in *D*. *ponderosae* and *I*. *typographus*. The amino acid sequences of all the 11 ORs are listed in S4 Fig.

In the OR phylogenetic tree, DvalOR2 was clustered with MacrOR1 and AplaOR83b, which are Orco homologs. In addition, a similarity search for the DvalOR1 against the non-redundant nucleotide (nr) database at NCBI using BlastX revealed 97% identity with the Orco homolog of *D*. *ponderosae* (AEE62122.1,0; DponOR1; Table 3) at the nucleotide level.

Four GR candidates (GR1-4) were identified in *D*. *valens*, which is in agreement with several other recent antennal transcriptomic studies [3,5,26]. Only one of these likely represented a full-length gene (GR3). Because GRs and ORs belong to the same superfamily, both were included in the same dendrogram analysis, in which GRs formed a distinct clade (Fig 8). The information of all four GRs is listed in Table 3. The amino acid sequences of all four GRs are listed in S4 Fig.

### Identification of Candidate Sensory Neuron Membrane Proteins

**Fig 9:**
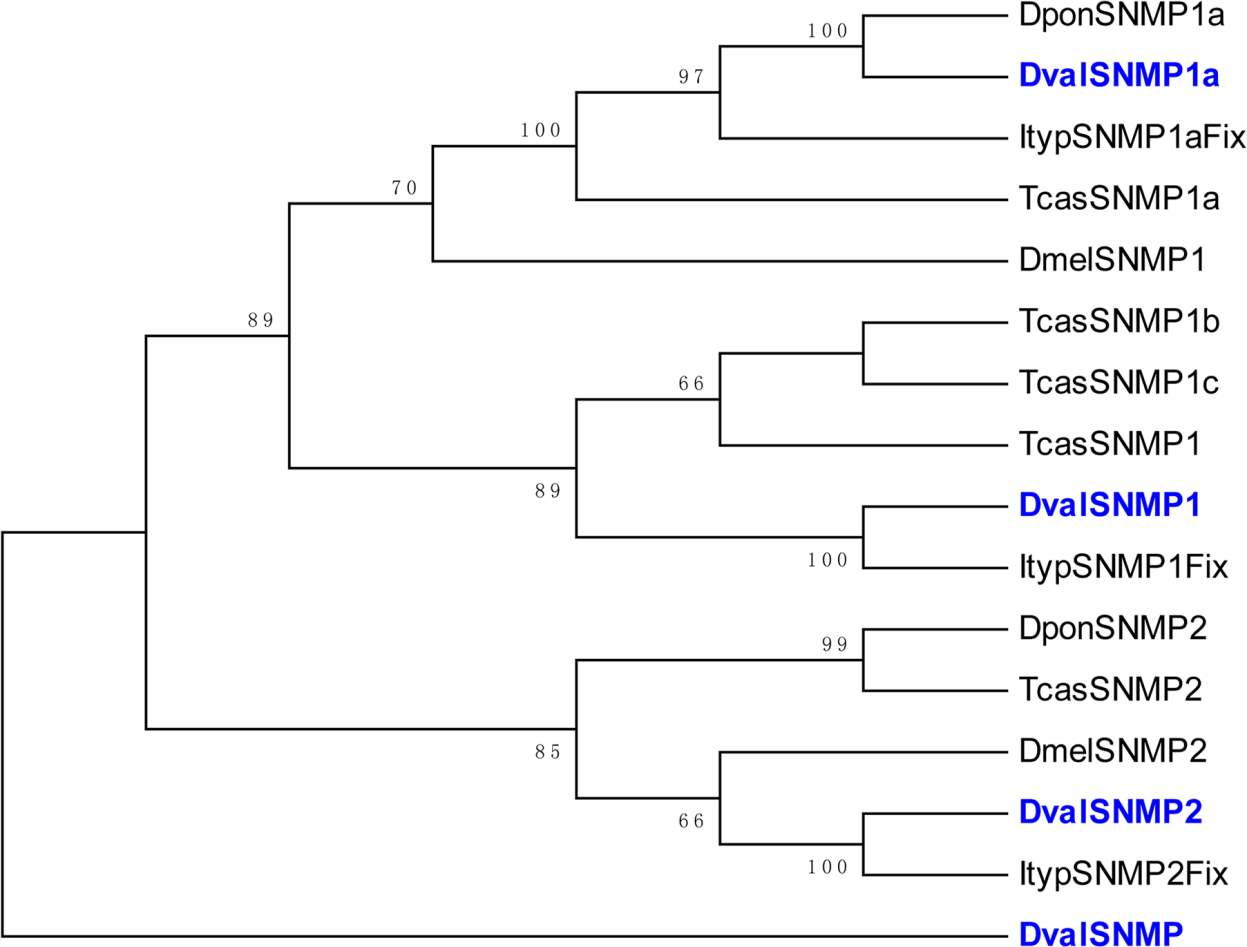
Phylogenetic tree of putative SNMPs from *Dendroctonus valens* (*Dval*), *Ips typographus* (*Ityp*), *Dendroctonus ponderosae* (*Dpon*), *Tribolium castaneum* (*Tcas*) and *Drosophila melanogaster* (*Dmel*). The *D*. *valens* translated unigenes are shown in blue. Accession numbers are given in S5 Fig. The tree was constructed with MEGA5.0, using the neighbor-joining method. Values indicated at the nodes are bootstrap values based on 1,000 replicates, and the bootstrap values below 50% are not shown.

We also found four SNMPs (Table 2) in the antennal transcriptome of *D*. *valens*, none of which represented full-length genes. Among them, SNMP1 and SNMP1a are orthologs of SNMP1. DvalSNMP1 and DvalSNMP1a were very similar to the DponSNMP1 and DponSNMP1a published in GenBank, with 94% and 95% identity, respectively (Table 2). The genes grouped together with orthologs in *T*. *castaneum*, *I*. *typographus*, *D*. *ponderosae*, and *A*. *planipennis*. We found 1:1 orthologous candidate (SNMP1, SNMP1a, and SNMP2) relationships among the SNMPs (Fig 9). DvalSNMP was scattered in a monophyletic clade in the outer part of the tree, indicating an ancestral relationship to the rest of the tree. DvalSNMP was searched against the non-redundant nucleotide (nr) database at NCBI using BlastX (BLAST 2.2.23+, E-value < e^-^5), and showed 85% identity with a sensory neuron membrane protein of *D*. *ponderosae* (GenBank accession number: AFI45067.1). The information for all four SNMPs is listed in Table 2 with the amino acid sequences listed in S5 Fig.

### Identification of Candidate Ionotropic Receptors

We discovered three antennal IRs from antennal transcriptome assembly in *D*. *valens*. IR1 and IR3 have high sequence similarity to the ionotropic receptor 8a of *D*. *ponderosae* (GenBank accession number: AGI05169.1) with amino acid identities of 87%, and 97%, respectively (Table 3), suggesting that IR1 and IR3 are homologs. We also identified eight ionotropic glutamate receptor genes (IGluR) (Table 3), likely as cell surface proteins which allow neurons to communicate with cells in the insect [20].

### Identification of Candidate Odor/Xenobiotic Degradation Enzymes

A large number of odor/xenobiotic degradation genes were found within the *D*. *valens* antennal transcriptome (155). These genes are likely involved in olfaction processes. Among the odor/xenobiotics degradation genes, the number of esterase genes (66) was the highest, followed by cytochrome P450 genes (49). Cytochrome P450s are thought to participate in detoxification, odor processing, and neuro/development functions in insects [52–55]. Esterases are thought to function in metabolic resistance and odor degradation in several insect species [56–60]. The remaining genes identified in *D*. *valens* antennae are involved in odor/xenobiotic degradation, including glutathione S-transferases (GSTs, 11), aldehyde dehydrogenase (14), epoxide hydrolases (four), and several antioxidants (Table 1).

## Discussion

The antennal transcriptome described here represents the first study on the repertoire of odor processing genes of the destructive and invasive red turpentine beetle, *Dendroctonus valens*. In this study, 21 OBPs, six CSPs, four SNMPs, 22 ORs, four GRs, three IRs, eight ionotropic glutamate receptors and 155 odorant/xenobiotic degradation enzymes were identified as putative olfactory system genes. These genes are not only involved in signal detection and transduction, but also in metabolic activity. It is well known that *D*. *valens* exploits host kairomones (such as (+)-3-carene), as well as non-host volatiles (e.g., 1-octen-3-ol, (*Z*)-3-hexen-1-ol, and (*E*)-2-hexen-1-ol) during host location, and uses aggregation pheromones and congeneric kairomones (*trans*-verbenol, myrtenol, myrtenal, and frontalin) in intra- and interspecific communication [36–50]. The odor reception and odor degradation genes identified here likely play important physiological roles in these aspects of *D*. *valens*’s biology and provide a foundation to further unravel the molecular mechanisms underlying olfaction in *D*. *valens*. Functional studies will be required to ascertain the specific role of these genes in *D*. *valens* olfaction. Furthermore, critical molecular targets of important semiochemicals such as (+)-3-carene, *trans*-verbenol, and frontalin could be identified via computational and molecular methods, such as homology modeling, molecular docking, and RNA interference (RNAi). By collating chemical and ecological knowledge with the putatively identified genes in this study, we can begin to elucidate the olfactory mechanisms of *D*. *valens*.

Prior to this study, coleopteran species’ odorant reception genes had been characterized from the genome of *T*. *castaneum* [51], the antennal transcriptomes of *I*. *typographus* and *D*. *ponderosae* [5], and the antennal transcriptome of *A*. *plannipennis* [26]. However coleopteran odorant degrading enzymes have received minimal study, except for the DponCYP345E2, an antenna-specific cytochrome P450 in *D*. *ponderosae* [61], and several odorant degrading enzymes in the antennal trasncriptome of *A*. *plannipennis* [26]. Odorant degrading enzymes are needed to quickly deactivate the odorants once the signal has been transduced so that the olfactory system can respond to continuing plumes of odor [2,62]. In the context of pest insect management, some of these genes or their products could be targets for development of specific inhibitors that interfere with the insect’s ability to respond appropriately to olfactory cues from mates or host plants[4,5,26]. The odorant degrading genes reported here provide a significant addition to the pool of identified olfactory genes in Coleoptera, and will advance our understanding of coleopteran olfactory molecular mechanisms.

In the *D*. *valens* transcriptome analyzed here, 70.41% of 22,058 transcripts had homologous matches in GenBank with the cutoff value of 10^−5^. Among these transcripts, 39.6% of the genes were annotated on one or more GO terms by GO analyses. More than 60% of the transcripts had no GO terms, similar to what was found with *Manduca sexta* [1], *I*. *typographus* and *D*. *ponderosae* [5], and *A*. *planipennis* [26] This result indicates high levels of unknown processes in antennal tissue and a large number of *D*. *valens* transcripts that may potentially represent novel genes.

OBPs play an important role in odor processing by insects, facilitating the transport of odorant molecules through the sensillar lymph and serving as the liaison between the external environment and ORs [2,4]. In this study, we identified 21 transcripts encoding putative OBPs in the *D*. *valens* antennal transcriptome. Even though the number of putative OBP-coding genes in *D*. *valens* is much lower than that of *D*. *ponderosa* (31), the majority of putative OBPs showed a high similarity to those of *D*. *ponderosa*, and most of the OBPs of *D*. *valens* clustered together with DponOBPs. CSPs are another more conserved class of small binding proteins which can bind pheromone compounds [6–9]. Here we identified six transcripts encoding putative CSPs in the *D*. *valens* antennal transcriptome. Among the six candidate CSPs, four sequences were clustered together with DponCSPs. *Dendroctonus valens* and *D*. *ponderosae* are sympatric in North America, and the sequence similarity of olfactory-related orthologous genes is very high between the two species, suggesting that the OBPs and CSPs of *D*. *valens* may have similar forms of expression and function as those of *D*. *ponderosae*. It was interesting that nearly one third of the transcripts which encoded putative OBPs and CSPs in *D*. *ponderosae* were identified only in non-sensory tissues rather than the antennal tissues [2,4], suggesting that these proteins might have physiological functions independent of olfaction.

SNMPs are membrane proteins observed to associate with chemosensory neurons in insects [24]. There are two representatives of insect SNMPs (SNMP1 and SNMP2). To date, the general mechanism of SNMP function is still poorly understood. Benton et al.[22] demonstrated that SNMP1 was essential for the detection of the pheromone (*Z*)-11-octadecenyl acetate (a volatile male-specific fatty-acid-derived pheromone) in *Drosophila melanogaster* and (*Z*)-11-hexadecenal (a lipid-derived pheromone) in *Heliothis virescens*, and suggested that SNMP acts in concert with odorant receptors to capture pheromone molecules on the surface of olfactory dendrites, particularly pheromones with hydrophobic tails [5]. In the current antennal transcriptome of *D*. *valens*, four SNMPs were identified. Because bark beetle pheromones lack the hydrophobic tails of *D*. *melanogaster* and *Heliothis virescens* pheromones, we assume that SNMPs may have an alternate functional role. The expression of antennal SNMPs in *D*. *valens*, similar to *I*. *typographus* and *D*. *ponderosae*, suggests that SNMPs may be involved in pheromone detection by bark beetles.

The IR family contains two major groups, the conserved antennal IRs involved in olfaction and species-specific divergent IRs that are expressed in other tissues including gustatory organs [63]. We identified three IRs from antennal transcriptome assembly in *D*. *valens*. The number of IR genes in *D*. *valens* is much lower than that of *D*. *ponderosae* (15). We could not exclude the possibility that some of transcripts were missed in our antennal transcriptome, because of the lower sequencing depth of *D*. *valens* than that of *D*. *ponderosae*. Sequence alignments showed that the putative *D*. *valens* IRs have high similarity with the known ionotropic receptor of *D*. *ponderosae*, DponIR8a. DponIR8a belongs to the group of IR8a, which is a broadly expressed co-receptor and necessary for odor responses[5]. Originally, IR8a was identified in the fruitfly *D*. *melanogaster* and it may form heteromers with another variant ionotropic receptor [64].

ORs belong to a large multigene family encoding seven transmembrane domain proteins [10,11], and are the key players in insect olfactory reception [4]. Twenty-two OR candidates were identified from the *D*. *valens* antennal transcriptome. This number is much lower than for two other bark beetles (*I*. *typographus*, 43, and *D*. *ponderosae*, 49). The lower number of receptors in *D*. *valens* may be due to technical reasons. Despite the number difference in the putative OR-coding genes, the majority of DvalORs grouped together with orthologs in *D*. *ponderosae* and *I*. *typographus*. In agreement with previous results, we found distinct expansions of OR lineages in bark beetles, and the subgroup 7b was exclusive to three bark beetle species in the OR phylogenetic tree [2,5]. In addition, DvalOR1 and DvalOR2 were identified as co-receptors in the *D*. *valens* antennal transcriptome. Further function studies on DvalOR1 and DvalOR2 may help in deciphering their role in *D*. *valens* olfaction.

GRs are mostly expressed in gustatory receptor neurons in taste organs and are involved in detection of sugars, bitter compounds, and contact pheromones [2,5]. In the antennal transcriptome of *D*. *valens*, four GR candidates (GR1-4) were identified. In the phylogenetic tree, Gr1, Gr 2, and Gr4 in *D*. *valens* grouped together with ItypGR3 and DponGR3, which may function as carbon dioxide receptors, and DvalGr3 was clustered with ItypGR6, tentatively assigned as a trehalose receptor [2,5]. So far, insect GRs have been identified as sugar receptors—BmorGR8 of *B*. *mori* [65] and HarmGR9 of *Helicoverpa armigera* [66]—heat sensors—DmelGR28B(D) of *D*. *melanogaster* [67]—and carbon dioxide and bitter receptors [68]. It is therefore likely that the *D*. *valens* GRs play important roles in the detection of carbon dioxide, sugars, or bitter compounds. Future studies on GR expression patterns of *D*. *valens* in chemosensory and non-chemosensory tissues (e.g. the head and maxillary palps of both sexes) will be helpful in understanding the actual roles of GRs in *D*. *valens*.

It is generally accepted that insect semiochemicals require specific odorant degrading enzymes based on the functional group(s) present on each semiochemical. In our study, a high number of odor/xenobiotic degradation genes were found in the *D*. *valens* antennal transcriptome. This is the first report of genes involved in odor degradation in *D*. *valens*. These genes, including esterases, cytochrome P450 genes, glutathione S-transferases, aldehyde dehydrogenase, epoxide hydrolases, and antioxidants, are likely involved in odor degradation. For insects, the olfactory system must not only precisely detect conspecific semiochemicals, but also rapidly inactivate the chemical signal once the message is conveyed to allow for continued reception of signals[2]. Mounting evidence suggests that various types of pheromones are degraded by antennae-specific esterases, aldehyde oxidases, aldehyde dehydrogenases, epoxide hydrolases, glutathione-S-transferases, and cytochrome P450s [2,57,61,69–78]. Given the diverse expression and functions of these odorant degrading enzymes, identification and characterization of the enzymes specializing in odorant degradation is challenging [62]. At present, in contrast to odorant reception genes, there have been relatively few studies of odor degradation genes in insects. The first odorant degrading enzyme identified was an antennal-specific esterase from *A*. *polyphemus* (ApolSE), which could effectively degrade the acetate component of the pheromone blend [59,60]. A glutathione-S-transferase in *M*. *sexta* (GST-msolf1) has been shown to play a key role in sex pheromone detection by inactivating the aldehyde component of the sex pheromone blend [79]. Cytochrome P450 is one of the most studied groups of detoxification enzymes linked to odorant degradation [62]. DponCYP345E2, an antenna-specific cytochrome P450 found in *D*. *ponderosae*, degrades several pine host monoterpenes [61]. This was the first P450 to be functionally characterized in insect olfaction in bark beetles. In another study, several antennal-specific cytochrome P450’s were found in *D*. *valens*, suggesting that these P450 genes may be involved in detoxification or odor degradation of these monoterpenes [80]. Here, we found 49 novel P450 genes in the *D*. *valens* antennal transcriptome, but their functional roles remain to be determined. Understanding the molecular mechanism(s) of signal inactivation is important in fundamental biology and may lead to novel molecular targets for insect pest control [2].

## Conclusions

This study reports the first antennal transcriptome analysis in *Dendroctonus valens*. The genes reported here provide valuable insight into the molecular mechanisms of insect olfaction, and also represent possible novel targets for *D*. *valens* control or management. In addition, the results from our study provide the basis for a deeper understanding of coleopteran olfaction in the context of complex behavior, and information for comparative and functional genomic analyses of related species.

Recent studies have shown that the numbers of functional chemosensory receptor genes vary enormously among the genomes of different animal species [3–5]. Despite the number of chemosensory transcripts being highly variable within the Scolytinae, the putative olfactory genes in *D*. *valens* demonstrated close similarity in sequence alignments to those of two other two bark beetles. The phylogenetic trees showed that the majority of the odor processing genes of *D*. *valens* cluster with the analogous genes in *D*. *ponderosae*. In addition, we also found a distinct species-specific expansion of OR lineages in our results. *Dendroctonus valens* and *D*. *ponderosae* are sympatric in their host range in North America, and the two species live in similar coniferous habitats and share a number of biologically relevant compounds, including host compounds, pheromone compounds and non-host volatiles (S2 Table). The low degree of species-specific diversification in chemosensory genes can be explained by the shared semiochemical space of the three bark beetles. Ecological adaptation and life history parameters might play important roles in shaping the olfactory gene repertoire [81]. The patterns seen in the current study likely reflect the evolutionary and ecological relatedness of these species. Further functional evidence is required to support this hypothesis.

The insect olfactory gene repertoire may be larger than currently understood. For example, nearly one third of the transcripts encoding putative OBPs and CSPs in *D*. *ponderosae* were identified only in non-sensory tissues instead of from the antennal tissue [2,5], suggesting that these proteins might have physiological functions independent of or in addition to olfaction. Here, we analyzed the antennal chemosensory repertoire of *D*. *valens* of a specific life stage (newly emerged adults) and a specific organ (antennae). As the first step towards understanding their functions, a comprehensive examination of the chemosensory gene expression patterns in the context of different tissues and life stages in *D*. *valens* could provide important information on the functions of the chemosensory genes. Following that, functional studies on the chemosensory genes will help to depict the molecular mechanisms underlying *D*. *valens* olfactory detection from an evolutionary viewpoint.

## Materials and Methods

### Insect and RNA Extraction

Adults of *Dendroctonus valens* were collected by Lindgren funnel traps baited with a kairomone lure (3-carene) in *P*. *tabuliformis* plantations at the Tunlanchuan Forest Farm (N 37° 48′, E 111° 57′, average elevation 1,400 m), Gujiao, Shanxi Province, China. Forest Pest Control Station of Shanxi Province issued the permit for the field

collections (by the director, Zhenwang, Miao). About one thousand newly emerged adults (650 females and 350 males) were collected. All *D*. *valens* were identified using the morphological characteristics of front and elytral declivity [82] and sexed based on distinguishing characters on the seventh abdominal tergite [83]. In total, 2,000 antennae from both sexes were used for RNA extraction. The antennae were cut off and immediately frozen in liquid nitrogen, and stored at—70°C until RNA extraction. The samples were homogenized using a Tissue-tearor, and total RNA was extracted using TRIzol reagent (Invitrogen) according to the manufacturer’s instructions.

### cDNA Library Construction

cDNA library construction and Illumina sequencing of the samples were performed at Beijing Genomics Institute Shenzhen, China [84]. Briefly, the mRNA was purified from 20 mg of total RNA (a mixture of RNAs from the antennae) using oligo (dT) magnetic beads and fragmented into short sequences in the presence of divalent cations at 94°C for 5 min. Then, the first-strand cDNA was generated using random hexamer-primed reverse transcription, followed by synthesis of the second-strand cDNA using RNaseHand DNA polymerase I. After end-repair and ligation of adaptors, the products were amplified by PCR and purified using the QIAquick PCR Purification Kit to create a cDNA library.

### Transcriptome Sequencing and Assembly

The cDNA library constructed from the antennae of *Dendroctonus valens* were sequenced on the Illumina HiSeq 2000 platform. Transcriptome *de novo* assembly was carried out using the short reads assembling program Trinity-2014 [85]. Trinity-2014 first combines reads with a certain length of overlap to form longer fragments without N (N represents unknown sequence) to produce contigs. The reads are then mapped back to contigs, by using paired-end reads that enable identification of contigs from the same transcript and the distances between these contigs. Next, Trinity-2014 connects the contigs based on the paired-end reads for gap filling between each pair of contigs to build scaffold sequences with the least Ns. Such sequences are defined as unigenes.

### Homology Analysis and Gene Ontology (GO) Annotation

The unigenes or contigs were matched by a BlastX homology search to the entries in the NCBI non-redundant (nr) protein database with a cut-off E-value of 10^−5^ to find similarities to the unigenes or contigs of other species. Unigenes larger than 150 bp were first aligned by BlastX to protein databases, including Nr, Swiss-Prot, KEGG and COG (e-value, 1025), retrieving proteins with the highest sequence similarity with the given unigenes along with their protein functional annotations. Then, we used the Blast2GO program [86] to get GO annotation of the unigenes, and got GO functional classification by using WEGO software [87]. The ORFs of the unigenes were predicted by using ORF finder (http://www.ncbi.nlm.nih.gov/gorf/gorf.html). The signal peptides of the protein sequences were predicted using SignalP 4.0 [88].

### Sequence Alignment and Phylogenetic Analyses

The amino acid sequence alignment of the candidate OBPs, CSPs, ORs, GRs and SNMPs from *D*. *valens* and other insect species (S1–S5 Figs) were performed using ClustalX 2.0 [89]. The OBP data set contained 21 sequences from *D*. *valens*, 11 from *Ips typographus*, 31 from *D*. *ponderosae*, 45 from *T*. *castaneum* and nine from *A*. *plannenis*. The CSP data set contained six sequences from *D*. *valens*, four from *I*. *typographus*, seven from *D*. *ponderosae*, 20 from *T*. *castaneum*, four from *A*. *planipennis*, and two from *D*. *melanogaster*. The SNMP data set contained four sequences from *D*. *valens*, three from *I*. *typographus*, two from *D*. *ponderosae*, five from *T*. *castaneum*, one from *A*. *planipennis*, and two from *D*. *melanogaster*. The OR data set contained 11 sequences from *D*. *valens*, 18 from *I*. *typographus*, 13 from *D*. *ponderosae*, two sequences from *A*. *planipennis*, 36 sequences from *M*. *caryae*, and 28 from *T*. *castaneum*. The GR data set contained three sequences from *D*. *valens*, three from *I*. *typographus*, and two from *D*. *ponderosae*. The unrooted phylogenetic trees were constructed by MEGA5.0 [90] using the Neighbor-joining method with Poisson correction of distances. Node support was assessed using a bootstrap procedure base on 1,000 replicates.

### Data Deposition

The sequences of 60 odorant reception genes (21 OBPs, six CSPs, four SNMPs, four GRs, three IRs and 22 ORs) of *D*. *valens* antennae were submitted to the GenBank database (accession number GenBank KP736107-KP736166).

## Supporting Information

S1 Fig Amino acid sequences of OBPs used in phylogenetic analyses. (DOCX)

S2 Fig Amino acid sequences of OBPs used for Multiple sequence alignment. (DOCX)

S3 Fig Amino acid sequences of CSPs used in phylogenetic analyses. (DOCX)

S4 Fig Amino acid sequences of ORs and GRs used in phylogenetic analyses. (DOCX)

S5 Fig Amino acid sequences of SNMPs used in phylogenetic analyses. (DOCX)

S1 Table Gene ontology of *D*. *valens* antennal transcriptome. (DOCX)

S2 Table Semiochemicals overlap within three bark beetle species. (DOCX)
