# Supplementary material for: Antennal Transcriptome Analysis of Odorant Reception Genes in the Red Turpentine Beetle (RTB), Dendroctonus valens
Source: PLoS One. 2015 May 4;10(5):e0125159. doi: 10.1371/journal.pone.0125159 (PMC4418697; doi:10.1371/journal.pone.0125159)
Supplement: S1 Fig — (DOCX) [file pone.0125159.s001.docx]

**S1 Fig. Amino acid sequences of OBPs used in phylogenetic analyses**

>DvalOBP1

MLTKTILIWAAILLTVFIPKGNCRLTEKQLAAAIKLVRNMCMGKSKANPEDIEKMHQGNWDVDYQAQCYMWCGFNMYKMLDKENHFDKKAALQQMDQLPIDLQEYVVKCMDQCENAVTNFDDKCVVAFEYSKCLYFCDPEKYFLP-

>DvalOBP2

MKQLVMVVLTALCVVHCKGLECGLSKISSEHFRKIASECVKDNETLNRIWELTSETSMEEDSVSSDEEVPVTKGREAPNFHDLGSSAHRNMKMSGASRTKRSRKGFNNESPMSNVQKKSSPASTTTEHTTTMQSEENEENAAANNVEESGEVCILQCIFEKLEMTDTNGLPDHKKVASALVKSASGRETQDFLQDSVDECFQETEEGDFENSCEYSTKLVTCLAGKGKSNCADWPVGDLPF-

>DvalOBP3

MHCSRACLIVFFSICGLSSSLKITLPPELQEYVDDLHKLCLEKGGLTENDHQTYDINHKNEKMMCYMKCLMLESKWMKSGGEIDYDFIETQAYPEVKDLLLNALNKCRTIEEGADLCEKSYNFNKCLYDADPVNWFFV-

>DvalOBP4

MNTALKVFLVALAIPTIMGLSDEMQELANQLHTTCIGETGAAEDAITNARNGDFSEADSFKCYIKCLLSQMAIIDDNDGTIDVDAMVAVLPEEIQEATEPIIRKCGSIIGANPCDSAWLTHKCYYKEGPEHYFLI-

>DvalOBP5

MSEKTHFALVALLLTCLVNIIDADQREKAVEFQRDCMEAHGLLEDELHEIMDGKPIQNEAFYFHFFCVVKKAKLISDNGIVNTDHFEENLKGVIDEENMAHVAALTRRCLIQRDDIFTTIKMAIDCFYSSEHKL-

>DvalOBP6

MKSIVALFVCALTATALADAEINETAFKAGRDRIMAMSRTCDENPATAVDQKALKKYLQSNGPAPANGAAHALCITKNLGWQNEDGSVNKPVITEKVKAIFGSVDAKVQQYIEECTEAKATPEDTAEQLLKCYRKHSPKIE-

>DvalOBP7

LYKNSPGHLALTQFGAISQEKKEKKVQIIKECAEESGVSRGAVLSARKGDFQDEPLLKQYFFCINKKSQIQNE

>DvalOBP8

MRLCFKDITGGVKENKPDRGFGNHRNFDLFSCEAVEKRKSDMTCVELCKLQKQGLISDDGSPKAEQISTYLREAFTTQTWFEKESQGIVDKCVNEAINATKNPVKFYTEGNKLCSRSGIVLKHCLFNSIQLSCPADQIKDKNACERFQERAKKGKDLFDQPPGPPPFDDNREE-

>DvalOBP9

MKSLAVVFAVLIAASLADQVQDIWDRVHQQCQQSPNTHVPQEIFDQLKRGETPILPANFGLHANCMLKKMNLQDNDGHIISSGVKEAAQRHYQSAEKINQIVKDCSATKKTKEETALNLFTCLGQNRVNIG

>DvalOBP10

MQLLFVAVLVIALVQVNSLTDKQKELLTQHYNQCVAISKVDQAVLQKARAGDFANDPNLKTHIKCISEKIGFQGTDGKFRRDVIEKKLKETLPGDNAKNAKLIETCVVANKDPQLQAFNAFKCLYTNAKINLL-

>DvalOBP11

MGARALNVYYTLKTSERSFLVKTSSESIAMKYLVVLSLCLAVVSAAALTKEEIKERLKAAHDKCQADPLTAIDEEALKAFKESKGKGQPPANLGPHDLCISKALKWQNADGKVNKELIKERIAENVADASKVDAIVSECAVDKENDIATAENLFKCLLRHHATALHAH-

>DvalOBP12

MHFQWLTNVSVFLCILGVAQLVAAGKPNDLFTRITPGDVEVCGKDTGVDRKDFEEAREKGALNHSMLCFLKCAMEKAGFLKDGHLEIDQAKEASPDKMTEPVVECFKAVGPISTCDDIQKVENCLPGS-

>DvalOBP13

MSNLLKLSIAFAVVSVISCQDFTEEQRKKIIENRQQCIEETKVNPDLIEKADLGDFAEDQALKCFTKCFYQKAGFVNDKGEVQKDVVEAKLPPQADKKRALEIVDKCALKGKDACETVYLIHKCYFEHTHPEADEKTAKDGKSEEKKA-

>DvalOBP14

LHNNSPRICILTMKLLIFASILVCASALDQSWRDHMKEKLTEFGIECAESEQATSEDIEALHNHKPPVTHAGRCVIFCVSKKLNLMNPDGTLNVTPQSDWIEKVKESDSEAFEKMKTVYHHCADTVEVEADACDTSLSYAHCIKEEGHKVGLYTVSAD

>DvalOBP15

MGTTIFLLVGLFMMTNAYVPNVNDKIRDFCIDDSGVSIEMVENLLANPEKELIDVESCYVHCIFTEMGLLSENGNVEIENFKSLKASEAPYIDLNCLEEIKSIDHCNEMMILRACHV-

>DvalOBP16

MKLMWILVLGAALKSTEGAMTEAQMKAALKLIRNVCQPKNKATDAQIAAMHNGDWNQDKNGMCYMNCVLNYYKLQLPDNSFDWETGLKVVESQAPPSMAGFIMETIKSCKDAVKTGDDKCKAALEITKCLYDQNPEKYFLP-

>DvalOBP17

MQVTMNQGWFLLLVSVVSVFAELDQTSLPPEAKELMAALHKNCIEQVGVSEADVDKLRAANFEEDANLKCYTRCLMAESGVMDENGAIDIEAFGEILPEAIRGNIQAIFRSCSLTKNDIVDQCVKAYEMVKCWHKENPESYFMI-

>DvalOBP18

MNGFSVFFLLLLAAVVKSDFDFSNYKEFENLAGDQREKAIKLFKECMAETGATHEMMEKSVEGDIPDDIVFKNHLVCIGKKSGFIDENGMHIKEKLKEKLTLLLGNEELVDKILDKCFMEKGSPQDTAFELAKCCHREYHN-

>DvalOBP19

MKAMFVTLTVATVVVFASADLTEEQKQKIVANGKACVAETGADPELIKAARQGKFADDAKLKAFALCMSKKSGFQNEAGEIQSDVVKQKLGLAIGDEAAAKKLVEKCLVSKGSGEETAIETFKCYYENTPTHIAVF-

>DvalOBP20

MKVFVVLCIVLFAFTLIVSAKKNKSNDEEKAKSYKKVFKECQKKDETRVDASIIRKLKKHKQVDLPANFGEHKLCVFTGIGLLKADNTVDEDKLKKKIASAKPQKDIVDNIVMDCTSSKSTLQETALNLDKCLTTYSIEF-

>DvalOBP21

MALTTWVLSIMLILPAIRALSDEMKELAQMLHNTCVAETGVNEDFIQKVNAEKIFADDENLKCYIKCLMAQMACIDDDGIIDEEATIAVLPEEYQALAAPVIRACGTKHGANPCENAWLSHRCYAEMEPSAYMLI-

>TcasOBP01

MKTVAVLLFLALAACTKQEDDDRQETIRQYRDDCIAETKVDPALIDRADNGDFTDDAKLQCFSKCFYQKAGFVSETGDLLFDVIKDKIPKEANREKALAIIDKCKELKGADSCETVYLVHKCYFLHSYGTDKKTE

>TcasOBP02

MKAFIVLVAVAVCAQALTDEQKEKIKNYHKECSAVSGVSQDVITKARKGEFIEDPKFKEHLFCFSKKAGFQNEAGDFQEEVIRKKLNAELNDLDATNKLIAKCAVKKDSPQQTAFETIKCYYENTPTHVSLA

>TcasOBP03

MKFLVVISTVLMANIVQGLTDEQKSKLEEYSKECLKESKVDESVLKEAEKGVYLDDPKLMNHVYCLVKKINSQKDKGELEVTQIKEKLMMQINDEKEVDKLIQLCLVQEKSARYSLGKCEVSS

>TcasOBP04

MRASAVFLSSFIISIQAAAFNNPEDELRRSAACLEQSKVSSESIKNLQIGNFDDDERLKEYLFCVSKNAGYQDPAGHLQHEMIRLRFKGGRYSDDTINEVLQQCGHQKDTPQETAFQFMKCAYQNAFPRNYK

>TcasOBP05

MKTIICFVFVLAGAWFCFQALTKEQIDKLEPISKECRELNGISEDTILKVRRGEAVNEPKLKNHVLCVSKKTGLASETGETNVEVLRTKLRKVSENDDEVNSIIQKCVVKKSTPEETAFEIFVCLRKVKPNFSPAN

>TcasOBP06

MNFVCVIFILVAIIGAHGLSEQQTEKLNQLSKECRALTGVSQETITNARNGNFEEDPKLK LQVLCIGKKVGIMNESSQIDENVLKAKLRKVSDNDEEVNKIYNKCAVKKPAPEETAFETI KCVMKNKPKFSPVE

>TcasOBP07

MKFLVCLLFVIVAANALTKEQKEKLDKISKECKNQSGVSQELIDKARTGELINDPKLKAQIYCVSKKAGLATEAGEINMDNLKTKLKKVAANDDEVNKIIQKCVVKKPTPEETAFEVYKCLHANKPNFSVVD

>TcasOBP08

MENRLVLLIVINTLLLAQAAAKQDFHKKCLASSGANADTIAKVRNGKFSNDPQTQKYFGCMLRSVGVVNQAGQLQVAALRKQVPKDMKRDEAMKIYMSCKDKKGANNDETAYLLYKCFWEASPRHVKIDGQ

>TcasOBP09

MKYLLFLTVITLTCGIFAFSLSNREQAIFLSTYSTCLETSKVDSERALRTASGIIDDEPKLKEFLFCINKQNGVQDDAGNFVKDAVRKRIEHPLLTDKTMEIIVNKCTRKRETGEETAYQFLKCSYFTIMNEKHQ

>TcasOBP10

MNIYTCLVLVVIAASAQHLTEEQKNNWRKWSNECKVLIGVSQEAINKIRNNEFDSVDDKIKKHGLCFAKKASLADSSGNIIINQIKIKLKRVIEDDEEVDRIVTKCTIRKNTPEETTFETFRCLRENSSKFVPV

>TcasOBP11

MKQIYCLITVVVLIPTLTALENEGQNPDTANCVALGGQRIKDSEIAKMAHCILTKTNLMTDKGTFNSNLLKERLRQSVHSDELVDKVVMMCTVEKETPLKSAFSGYKCLRYLVPWFPLD

>TcasOBP12

MKIVLCLLALATVALAKKCFLAEDTDKLEVMINECKTKTGVPDDILQKARNGEKIDDPKLREHALCMMKKSEMMNDAGEMQMDKIRARIKHAVSNEAEGTRIMNECAVKKDTPLATAYEMICCLIRNKNSVDE

>TcasOBP13

MKVFVCLAVFALVAAAQAETAKEKLRKYSDECKSVSGVSEELLNKVRNHEDVHDPKLDEHGFCILKKAGFMNEAGDILADTIKTKLKENSEHPDTVDALVEKCNEKKDTPQHTASHLFTCLVDKKVHSH

>TcasOBP14

MKLFILLSLLSVCYARKWFDKDPQDVAKWQKECFEASGVSMESMNKLPNITLSEDPKLGENAFCLLKKLGFISEDGTLLIEKLRTSLKNQWGDEIANKLVNECARQKSTPQETAHEMFLCIPAKLK

>TcasOBP15

MKIVLICVLIGLVVAKQQKQDTLDEEKEKMKKWTQECIQESGVTSEILQQLRNQKRVEDPKLKEYTFCTFKKNGFMNEDGKLQYDVIKSTLMKVSGSEEEANKVVKDCVVEKSTPQDTAFETVDCWYRYKKN

>TcasOBP16

MIRYYIVLLLYFFAPPGISEEMQELVNQLHSTCVAETGVSEDLINKVNSDKVMIDDEKLKCYIKCLLTETGCISDDGVVDVEATIALLPEDMKAKTTPVIRSCGAKMGANPCESAWLTHKCYLETSPADYVLI

>TcasOBP17

MSPLLLIFISCLFPRGISEEMQELANTLHATCVDETGVSEDAIESARKGNFAPDDKLKCYMKCIMEQMACIDDEGIIDVEATIAVLPEEYQAKAEPIVRKCGTKIGANACDNAFLTNKCWYEEDPEVSLQLN

>TcasOBP18

MYKTRVIYVLFALCLVEIFVSRAIEMDDDMKELINNLHNTCTGETGATDDQIENARKGNFAEDDSFKCYFKCVFDQMGCMTDDGKVDSEAVIAVMPPELADKIASTVRGCTEVGANPCETAWLANKCYQKSNPDVSKVSSNVRSD

>TcasOBP19

MSRMLPAALFVVLATLTFATAEIVVPDDLKDYINELHDHCLKEMGLTEGDHKNYNIHVKDPKMMCYMKCLMTTSKWMNMDESIQYDFILSSVHPAVKNILLPALDKCRDIPKGTMECEKAYNFNMCLFNADPENWFFI

>TcasOBP20

MMPLKNLIILIVCPLFVDIPPDLQAEIDGYYDICYKQIGLTKDDLKAYKIGDRDPKIMCFMKCVFVEAKWMDENENLQYDYIKNTIHHSIRHITLPELENCGKKAEGDKCEKSFSFFNCMNKAEPEDWVLIQ

>TcasOBP21

MMHLKNFVVLVVCPLFVEIPPDLEAEIDEYFEQCFEPNGVTMDDIKAYKMGDKDPKIMCFMRCLFVSGKWMDENENMQYDYIKETIHHAIRHITIPELENCGKEAQTGDKCEKSFNFFMCMNRAEPEV

>TcasOBP22

MKMCVIFTLLLLVVLASAEEDNVGKIESVEKKCQEKTGVSEESLQKIMRLEEVDDPLVKE NALCTLKAYGVMDDDGNIFPDKFEEKLKPEIGADEAKRVAEKCAVKKDSPEETAHQTLWC ATEENALTDTSQEQ

>TcasOBP23

MKISTLVAILVLAGSAVCADEDNLNTENVQSIEEDCQKETGVSDESLQELSETGDSDDPLVKKNALCILKAYGVIDDQGEISEDKLEEKLEPDRGKEEAEKVAKSCAVKKDSPEETAHEALLCMQQKSQK

>TcasOBP24

MKSFVIFVLIIVITGQINATPSLDDFKKVQKDCQKKTGVSDESINKVNNLEPVCDDLLLQENALCILKTYEVMDEEGKICPDKLMEVLEPKFGKEKAEKLIEKCTLEKDTPQLLAHATLFCLSVQKYVV

>TcasOBP25

MNSVLFLLVCALLDKEFLMQFLQKIKKVSEDCIAETQATKNDIKTLLEHKIPDSHEGKCMIFCFHKHFQIQNEDGSLNKVAAISLLEPIKDHSQDIYDKVVKIFNTCFDSAERDDDSCIYASNLAECAIRESKSVSVQ

>TcasOBP26

MKLFILAGILFTGVCAVDQEFVEKFLQKMEKIGEECAEETHATSDDIADLIEQRDPKTHEGKCLIFCYHKKFNTMKEDGSLDKVGSVLALEEVRDADFELYKNILTIFVTCGDKAKIYDDPCETATALTMCGRDEAKAVSWAYFA

>TcasOBP27

MKSTWFFLLLACSLTCAFLEKMQEFGAQCAEETDATSDDIAELIARKLPPSTHEGKCMIFCMQKKFNMMKENGGIDRAGAIAALKPLQKADPELHQKVLKIFVTCGMRVKPSPDPCDTATELALCGKKEAEAVFCRLGWKTLS

>TcasOBP28

MKYFVVFASLFLATNALSQDFIDKFVAKVKSIGETCVPETNASKDDISSLLAHKMPDSHEGKCLIFCFHKQFQIQNDDGSINREGAIKALEPLKADDAELYEKVISIFKKCESTPVDGDSCLYAASLAECAVKEGRAVSFWKNTNFKLI

>TcasOBP29

MKFLLVFLSVAILCTFAMDESFLQQTRDRVKAIVKECVTEEKATDSDFDDIMALKIPTSHEGKCVFFCSHKKFNMQHPDGSINKEGALDTFEVVKDVDAEFHDKVITVYNHCLSTPVDPDPCVYSVNLFQCFMKEAKAVRK

>TcasOBP30

MKLLITLATLVVATYAIDKEFVQELRQKLRSHVEACAKEVNAGPDDVSAIFAHKLPATHEGKCIFFCMHKLYNAQNEDGSLNMAGALANLELIKDMDPDVYTKVSTSFKNCESAPFDSDPCLYAANLVTCIVKEGRAVSNNFSGFFF

>TcasOBP31

MQLLVVVLAVCVLGANAGVSIILDPKFLEKLTQEVQAVGTSCGEKEHATADDMIEIMEEKFPPTSHEAKCVVACFYKHYKMMKEDGTFDKDAAVKAFDEIKAQDAEIHAKILKVIDACDAKKQMSDDHCVSAASMAGCVKTEAIAV

>TcasOBP32

MLILIAVNCHKCIIVSALSLSATVFGQSLSEDEMRENARKLMTSCKDKVGASDADVEALKMHQMPESREGFCMLECVFDSAKIMQDGKFSKSGMIEGFKPLIGDDKAKLESLEKLSATCESELGDGEDKCETAKRLVECVIKNGKTHGFEVPPPRE

>TcasOBP33

MNFFAICLCFVASTVGVSSEENDINEIRSVEENCQKQTGVSVEKVNNFELVDDPLVKENALCILKAYGIMDEDGNIYEDKLKEQITSELGEKNAEQVAKKCTIKKESPQETAHESLWCVGEQKPIPGASPDEKN

>TcasOBP34

MSKTVFIFVIFFYLDFYATGDESVYLSNHEACVKLSGVDETLLETIYEGDVFEDMKFKTYIHCFFKKSGFQDENGVMHFDAIKSSFHKDFSQTENIDKTITECEEKKLNGESALETAFLHFKCFMGEL

>TcasOBP35

MKAILLLLVATLSFYHMSEAQMKAALKLVRNVCQPKTKATNEQIEAMHTGNWDLDKNGKCYMWCILNMYKLIGKDNSFDWEAGIATLKAQAPESVRDPAIASVNNCKDAVKTTSDKCEAAYEIAHCMYLDNPEKYFLP

>TcasOBP36

MKYFPHLCLCLIFFELSEAAMSEAQLKAAVKLVRNMCQPKSKATNEDIEKMHHGDWNIDRTAMCYMHCALNSNKLITKENVFNRDYAITLAEKNLPTALKTASIEAANLCKDSAKTLDDKCVAAYEISKCLYESNPEKYFLP

>TcasOBP37

MILKASIFLILAVATFGAILEDSELMKVVENCVKKTNANESEFSSPNFLETTPSQPALCTAKCLLESLEIVNSEGNINMETLKEYAQPFESPAREAVATCGEEIKSVTTCDDMEKYRKCVEPLIKNS

>TcasOBP38

MAKKQLVLFFLAFIFLQKFAEVREECLSENSMTMDELHEGWKMENLPESHLCFLKCLLEKREVIDENGVPQKEKIDEILTVKQLSDEKREEISTCITNVEKIENCETMSEIMRCFPKKRRD

>TcasOBP39

MAKMCRLFVVLSLFVASQALDLMADKNFVELRNKCLDKLGLKEEDLRDLKFDGDVSEDLMCFGKCIQEEDGLLDSEGNLNEEKLEKKIETMPFLSRVSDDTKNNIMECLKEIGKIETCQDFGKQRDCIHKYV

>TcasOBP40

MNPITSVILTFLFVFSFGEKESEEAQIFTELDGPAAELRDQCLEKNSMKVTDLKTYNTSNDIPEKELCFYKCFYEGVEFIDANGNLNVNNMKEIPAISELGDEVLNEITACVEKIGKIRCCGDLRKIEQCYQNITM

>TcasOBP41

MWSFVTLLFSFLVLASAQGKYWTTISECLTEHSMGVEDMKKFDLPAEKMSEEMLCFNKCFYDKLLITDENGEINTDNLMSIPLVNAIDASKHDDLVTCLKKVGKIEECDGVKKIEQCFVEFI

>TcasOBP42

MATRFCFGLLILFVGTVLVHEILEVRALCMNETGVSEETARNYKPAEDPASEEILCMVKCIFEKIGCLKDDGSFCVDTMKKKNYIMDVINEENEEKIYECLRGVGKITNCRDMAAVEECFVKNDSK

>TcasOBP43

MSFLILLICVIPAIFCRSFSHDELDTDLSFIKTCNRTSPISMSKFGLFLTEFNLTEPGTMNECFLHCLFMKYGWMDSDGGFLLHDIKQTLEESDVEIASLEFILYKCTATESNNRCERAFVFTQCFWDKMAEQQPSEDQFFYNIEDKK

>TcasOBP44

MKKTILLCLLSQLLLLKAAELQPEDRHQIALQCIDIVGIDQKVVEDAINIEIPKNNPKYKEFLACSYKKQGYQNENGEILMENIKKFLQKFYHPSDLQELNSCSGHNGTNHAENAYQALQCIYNRLSNMTVVGN

>TcasOBP45

MKPIFAIITLTLCTTVHSAIRPWRTCGTWPPCPPNGKMLQNFRIKRASVRLTNTETNETTPEPKAVSSEAQATENCIIQCIFDNLQMTDSTGYPVHTKILDGLLKNTTNRELRDFLQDTTDECFQVMDKEDTMDPCSYSNKLVTCLAEKGRSNCADWPVGELPFKP

>TcasOBP46

MLWRRGRFLYGENLDMFDPAGLQACMKKLSVGETELAKALEDKSKDPPEKIMCLFKCALEDSGFLQDGVVDKSKWPMPECVQDVVKITNCNDMVALKHCFD

>ApOBP1

MWLKFALIAVLPFILCISEEMMELAKMLHDNCQGETGVPEEMIENARKGDFADDDRFKCYLKCVMVQMAVMNDEGVVDPEAVVAVLPDELKDVLSGSIRACGGKVGKDQCENAWLTHKCYYEKEPEHYFLV-

>ApOBP2

MSSSLVVPLLIVGFAVANSFSENRHHAPIFEVCCEFEPFMAAPEDEKMKECISEILGDPKSMNHSNPPTDQELEELAEKIACIEECTAKKYELLDSDGNIVLAKLLEVAQGKINGTFMENHIEEAAKKCIEDIEKEIPKESKCNPKPLFLSNCLFFRSLENCPADQVKDKAKCETMIDEIKNGKFPHHFDPPPPEN-

>ApOBP3

MTTVTYFVAAVLLFCYYTNTNALLENYPPPEVLEYLKPYHTICTEKIGVSDDEVKNYKIEDNSEKMMCYMRCLGLESKWLTPDNKLQIDYIMETRLDSIADLVKNIVDNCKDVPDGTHECEKAYNLHKCAAKIEPERWFLP-

>ApOBP4

MRIFLLLLSCAFSFNRIEATPMNEAQLQNAAKLIRNVCQPKLKISDKLIENIHNGDFAENEKVMCYLECVLRMGQLMKNGKFDEKAALSQISTLPPERQQATKDSIKKCADKGQDDDKCVAAFETAKCIYFDNPQNYFLP

>ApOBP5

SIVVLYLISTNADPGKSSTIEIREWENNLIKDELSCINSTGVSLSVIERTKVTLELPEDDPKYKEYLKCFYTKRGYQSDSGEVLYDNIKIMIHQFTNATEATRIIDLCKEMRGATA

>ApOBP6

MNEAQLQNAAKLIRNVCQPKLKISDKLIENIHNGDFAENEKVMCYLECVLRMGQLMKNGKFDEKAALSQISTLPPERQQATKDSIKKCADKGQDDDKCVAAFETAKCIYFDNPQNYFLP-

>ApOBP7

MTDEQKAKLKQHQDECLKSFPADKLLLEKARKGDLADNKTLKDYLYCVIEKSGFITPDGKIQTAVLETKLASVTAAENAKKLVAKCTSQKNLDKKESAEAIYKCVYNETKFSLI-

>ApOBP8

MSSSLVVPLLIVGFAVANSFSENRHHAPIFEVCCEFEPFMAAPEDEKMKECISEILGDPKSMNHSNPPTDQELEELAEKIACIEECTAKKYELLDSDGNIVLAKLLEVAQGKINGTFMENHIEEAAKKCIEDIEKEIPKESKCNPKPLFLSNCLFFRSLENCPADQVKDKAKCETMIDEIKNGKFPHHFDPPPPEN-

>ApOBP9

MFRLGFIVLVALFLGNVAAEEVNPFEEDIQHLLEMRKICEEKFPVSKETVESLKQGIYPSEADDPNVCENVLCVIKGRGFVDEDGNVMQKNFPEKMWNAMPENCKENRGSDICEKVKNFLPMSICRFSTAMGTNECRHLTRLDYHVLLNQL-

>ItypOBP10

MNAFICMFLVFGVVKAYDFSDSIFNDHLNQIYYTLDNWQHERIRRNAEDVELKCRKPPPPMPKPCCAQDSFRDLMDKEREVLRDCFKEVVGEEHHPGRSNHPNKFDMFSCEAVEKRKNDIICIKQCLGSKLGLVNKDGKLDQAQIGNYVKSTFKNEAWLSPLADQIIGKCLVEAESVAPPKFHIEKLKPCKPSVITFKHCLDREIQLNCPADQIHNQESCERFRNHLNHKNDFDEDQPMMGPPDDD

>ItypOBP11

STGDSTMKFILLVIVVGQMGCVFGAMTESQMKAAFKLIRNVCQPKNKATDAQIEAMHKGDWNQNKNGMCYMNCVLNYYKLQLPDNSFDW

>ItypOBP12Fix

MLTVGKLVLVLVLVLIETSALQKTNNKCEIPTAAPKKIEDVINTCQDEIKIAILSEALEALNINEHKVSRKRRSTFNDDEKKIAGCLLQCVYRKMNAVNQYGFPTVDGLVSLYTEGITQKEYVLATLQSVTKCLGKAQKTYDIPAQNGTASTACDVAYGVFDCVSEEVAKYCGQTP

>ItypOBP14

MYGSVLKVSLVFAVISVISCQDFTEEQRKKIIQNRQDCIQETKVNPELIEKADQGEFIDDQALKCFTKCFYLKAGFVNDEGEVQKDVVEAKLPPQADKKKALEIVDKCAVKGKDACETVYLIHKCYFEHTHPDLPAKAEEEKKA

>ItypOBP15

MIQHHRTNSDLTAVKMKILAVLFVICVLFQFTIARNGGNLHYSKISMKKVQKRCQKNEESRIDPDVLKKLRKGEEVVQLPDNFPDHVTCLMKGMEYLNDDNTVNEEKVRNMVQRRVTDDQDVDAIVGECKAVKTALKETALNLINCLRKHELLWNHNFHD

>ItypOBP2Fix

MNSVAVFAVLALGAVCVIDAYNFQDEDFXSAVVVRDGRIVDSIDSGPVHPRVRRDQEAATVAEEKCPKRHRRPKLCCAEETLDALHAKKKEITKACFKEVTGLEKQDRHDHGPHFKRFDLFNCKEVEKRKSDMICIDQCVGQKKGLLDDSGAPIRDQLIQHLKQHFSNESWFDQTVVEKITSNCLAAAKNATETPIKFSTEGLKACNPSGITLKHCLFREIQLSCPADQIKDKTACDRFQDRIQKEIEIDDLRLAPDDQQ

>ItypOBP3

MATGKVFVYFFVVLFLSEQSVSRMTEKQLAAAVKLVRNMCLSKEKAKLEEVDKMHEGNWDIDHKTQCYMWCVLSQYKLIGKPNHFDRESANIQVDTLLPESMHDYVVGCLDKCENAATNFDDKCVAAYEYAKCLYFCNPKEYFLP

>ItypOBP4

MISAVIFFALVGTIFCADADLTQQQKDKLLADGKACVAETGVSTDLIQAARQGKFTEDDKLKAFSFCMSKRLGFQNDAGDIQTEVVKQKLGGALGDLGVAAQLVTKCLVPKATPQETAFESFRCYYQNTPTHLTVF

>ItypOBP5

MRQPGGNNKNTQQDYEMWTPSTGYQPSGSNNDFNVRTRYDGNTRFNRPSSEECRDQGNGNIPRSPFGSSNLPRRQRSSYFNREDDDNDNDCISQCVLGYMQLLDTDRSPSETLIIKWLQEHVTRNEMDRIKALRDTRKCFGKLVTTDIEDGCEYAKELSKCLELDLE

>ItypOBP6

MVLYLCDLVQLAPTNQTEEVTTSTKRQLTREKKKKIGKTCMLETGVRIETILRAIKEDIPKNDEKYKSYLVCSYKKQGYLSEDGGTMLYDNLYSFLQESAGYAKEDLHYIDDCKTITAETPGDLCLKKLVGILDGLHKVEKNREIDTNTIES

>ItypOBP7

MKVLFAFVCLVVLIQVNSQTDKQKELLAQHYKECLAKSKVNEATLQKARIGQFADDDKLKEHILCVAQKIGFQNSAGQFQNQVIETKLREALKGDAAKTKKLISDCAITNPDPKLQAFNAFKCVYQKASINLL

>ItypOBP8

MTKLQIVLLLTTLMGSFNIITGEKKCNSSNCMYDRMLETVGKEFIEQCFKETGVTPEDIRSVMEQNGYGEKQIVFPKMLDKENWYFGKRWSNQYRLY

>DponOBP1

MFGFRKIFLVLVLVVFESLALQKNNKCDIPLSAPKRIEEVINTCQDEIKIAILSEALEAFKVNEHKVVSRAKRSAFNEDEKKIAGCLLQCVYRKLNAVNEYGFPTVEGLVSLYTEGVTQKEYVVATRQAVTKCLENAQKTHEISTKTVEASKSCEVAYEVFDCVSLEVAKYCGQTP

>DponOBP2

MNKLVTLYAVLLGAACHLVQTYDFQDATFNEILSSDFEDIFDTLDNTYLHPRAKRNEEAVNSDEKCRRRHHRKPKLCCGEDVLDSLQEKEKEIVRLCFKDITGGVKESKPDRGFGNHRNFDLFSCEAVEKRKSDMICVEQCKLQKQGLVSDDGSPKPEQISTYLKEAFTTQTWFEKVSQGIVEKCVNEAINATKNPVKFYTEGNKLCSRSGIVLKHCLFNSIQLSCPAGQIKDKNACERFQERAKKGKDLFDQPPGPPPFDDNREEQI

>DponOBP3

MSINEIFVWRKLNIDLKMKSLVALFVCAFTATAMADAEINQSTFEAGRNRIMEMSRTCDENPATAVDQKALENYLESNGPAPANAGVHALCITKNLGWQNEDGSVNKPLITEKVKAIFGSVDAKIERYIEDCTEAKAKPEDTAEQLLNCYRKHSPKTE

>DponOBP4

MGKQAGFINEAGDVLKDVLKEKSLKLFNDPALVQKLIDQCIVKKETPQETSYHAHVCLYKNSPGHLALTQFGAISQEKKEKKVQIIKECAEESGVSRSAVLSARKGDFQDEPLLKQYFFCINKKSQIQNEAGEYKTDVIRKGLTELFNAEEANRIIEKCARIQDSALNTAFQSFKCFYNEAPEITGVF

>DponOBP5

MKTALKVFLVALAIPTIMGMSDEMQELANQLHTTCIGETGAAEDAITNARNGDFSEADSFKCYIKCLLSQMAIIDDNDGTIDVDAMVAVLPEEIQEATEPIIRKCGSIIGANPCDSAWLTHKCYYKEGPEHYFLI

>DponOBP6

MFKSVSFLLLILALGSLDAKITLPPELQEYVDDLHKLCLEKGGLTENDHQTYNINDKNEKMMCYMKCLMLESKWMKSGGEIDYDFIETQAYPEVRDLLLSALNKCRTIEEGADLCEKSYNFNKCMYEADPVNWFFV

>DponOBP7

MKVFIVAFVVLGVVFAVNADLTEEQKQKIVANGKACVADTGADPELIKAARQGKFADDAKLKAFALCMSKKIGFQNEAGEIQSDVVQQKLGSAIGDNEAAKKLVEKCLVSKGSGEETAIQSFKCYYENTPTHIAVF

>DponOBP8

MDEPTFNRFAYLKANFPIGTQLHNTSPRICILTMKLLIFASILVCASALDQAWRDHMKEKLTEFGLECAESEQATSEDIEALHNHKPPVTHAGRCVIFCVSKKLNLMNADGTLNVTPQSDWIEKVKETDSEAFEKMKTVYHHCADTVEVEADACDTSLSYAHCIKEEGHKVGLYTVSAD

>DponOBP9

MKVLVVLCIVLIAFTLIVSAKKNKSNDEEKPKSYKKVFKECQKKDETRVDASIIRKLKKHKQVDLPANFGDHKLCVFKGIGLLKADNTVDEDKLKKKISSAKPQKDNVDSIFTECKSSKSTLQETALNLDRCLTTNSIEF

>DponOBP10

MLTKTILIWAAILLTVFISTGNCRLTEKQVAAAVKLVRNMCMGKSKVNPEDIDKMHQGNWDVDYEAQCYMWCGFNMYKMLDKENHFDKKSALQQMEQLPTDLQDYVIKCMGQCENAVTNFDDKCVVAFEYSKCLYFCDPEKYFLP

>DponOBP11

MKSLVVFCALLVMALAHDPHGLDSVHKECHNEVASQHYLCMAKGLHLVTPEGKVNVNGVKTHAGHVVSESAKIDQIAKECAVDHASTEETVNHLFKCLEEKHVLSLAGHVAPQHHH

>DponOBP12

MKLMWILVLGAALKKADGAMTEAQMKAALKLIRNVCQPKNKATDAQIAAMHNGDWNQDKNGMCYMNCVLNYYKLQLPDNSFDWETGLKVVESQAPPSMAGFIMETITGCKDAVKTRDDKCKAAVEITKCLYDQNPEKYFLP

>DponOBP13

MFPAIKFLIVLGVVAVATRADRQQVVDFHRPCLDHHEIEDDDLHFALDKIKMRDDDEFYLHFFCVAKQGQLMTEDGTVNTDNFETNMKGIIDEDNMENVAAIVRLCLIQKDTVLQTIRNAVDCFMGKDHKL

>DponOBP14

MPVTMNQGCFLLVVSAVLVFAELDQTSLPPETKELMAALHKNCIEQIGVSEADVDQLRAANFEEDAKLKCYTRCLMAESGVMDENGAIDVEAFAEILPEAVRGNIQTIFRRCSLTNKDIEDQCVKAYEMVKCWHKEDPESYFMI

>DponOBP15

MMTAFLLCVLMAVVNQQVMGHPPRGPPGPPPFLGHPDPESANECRTEVGLTSEDRETKKNGELTEKELCFIRCLGQKNGALSDAGALNIETIKNDLPDHLEDSEAVIACLKKVGTVTTCQHIKKVAKCYPEPKEPMDRT

>DponOBP16

MGPTILLLVGLVMMTNAYVPNVNNKIRDFCIDDSGVSIEMVENLLANPEKQLIDVESCYLHCIFTEMGLLSENGNVEVEKFKSLKASEAPYIDLTCLEEIKSIDHCSEMMILRACHV

>DponOBP17

MALTTWVVSIMLILPAIRALSDEMKELAQMLHNTCVAETGVNEDFIRKVNAEKIFADDENLKCYIKCLMAQMACIDDDGIIDEEATIAILPEEYQALAAPVIRACGTKHGANPCENAWLSHRCYAEMEPSVSG

>DponOBP18

MALTTWVVGIMLILPAIRALSDEMKELAQMLHNTCVAETGVNEDFIRKVNAEKIFADDENLKCYIKCLMAQMACIDDNGIIDEEATIAILPEEYQALAAPVIRACGTKHGANPCENAWLSHRCYAEMEPSAYMLI

>DponOBP19

MNGLCVFFLLLLAAVVKSDFDFSNYKEFEHLAGDQREKAISIFKECMAETGATHEMMEKSVEGDIPDDIVFKNHLVCIGKKSGFIDENGLHSKEKLKEKLTLLLGDEGLVDKILDKCFMEKGTPQDTAFELAKCCHKEYHN

>DponOBP20

MKQLFMVVLTALCAVHCKGLECGLSKISSEHFRKIASECVKDNETLNRIWELTSEASMDDESASSDEEVPITQGKEAPNLDLGSSPHKSMKMSRASRTKRSRKIFNNESPMSQRKPSPASTTTEQTTTVQSEENEDIADANNVEESGEVCLLQCIFEKLEMTDTNGLPDHKKFAAALVESATGRETRDFLKDSVDECFQETEEGDFEDSCEYSTKLVTCLAGRGKSNCADWPVGDLPF

>DponOBP21

MKQLFMVVLTALCAVHCKGLECGLSKISSEHFRKIASECVKDNETLNRIWELTSEASMDDESASSDEEVPITQGKEAPNLDLGSSAQKSMKMSRASRTKRSRKSFNNESPMSQRKPSPTSTTTEQTTTIQSEENEDNADANNVEESGEVCILQCIFEKLEMTDTNGLPDHKKFAAALVESATGRETRDFLQDSVDECFQETEEGDFENSCEYSTKLVTCLAGRGKSNCADWPVGDLPF

>DponOBP22

MSQKIHFALVAVFLTFLVNIIEADQREKAVEFQRGCMEAHGLLEDELHEIMDGKPIQNEAFYFHFFCVVKKAKLISDNGIVNTDHFEENLKDVIDEEHMAHVAALTRKCLIQRDDIFTTIKMAIDCFYSSEHKL

>DponOBP23

MHCLRVCLIVFFSICGFSSSLKITLPPELQEYVDDLHKLCLEKGGLTENDHQTYNINDKNEKMMCYMKCLMLESKWMKSGGEIDYDFIETQAYPEVRDLLLSALNKCRTIEEGADLCEKSYNFNKCMYEADPVNWFFV

>DponOBP25

MSNLLKLSIAFAVVSVISCQDFTEEQRKKIIENRQQCIEETKVNPDLIEKADLGDFAEDQALKCFTKCFYQKAGFVNDKGEVQKDVVEAKLPPQADKKRALEIVDKCALKGKDACETVYLIHKCYFEHTHPEADEKTAKDGKSEEKKA

>DponOBP26

MAKTTIVLCLMGIFCMRSVQPNPVRTHKLLSKSELHEIATSCLEEVQLSGSIVYNILKTEIFPRDNNKYRDFLACSYKKQGFLSEDGTKLLYDNLFHFISHFYGPTEVQALKHCNLIRREDPGFLCFDTMKCIIDALKQLEFDANADIGIETNQVV

>DponOBP27

MNFSFIVALTLYLPISNGFLTVPKCLISTGARIKDLHNLATGDSLPESSRCFVKCVGEESGLILDGTLHSEHFEALPMVSRLKADVFVDARRCIESVQGIKIESCKDIDNLNDCMKIVYRQKYSDSK

>DponOBP28

MKYLVVLSLCLAVVSAAALTKEEIKERLKAAHDKCQADPQTAIDEAALKAFKDSKGKGQLPANMGPHDLCISKALKWQNADGKVNKELIKERITDNVADASKVDAIVNECAVDKENEIATAENLFKCLLKHHATAVHGH

>DponOBP29

MKAMFVTLTVATVVVFASADLTEEQKQKIVANGKACVADTGADPELIKAARQGKFADDAKLKAFALCMSKKIGFQNEAGEIQSDVVQQKLGSAIGDNEAAKKLVEKCLVSKGSGEETAIQSFKCYYENTPTHIAVF

>DponOBP30

MQLLFAAVLVIALVQVNSLTDKQKELLTQHYNQCVAISKVDQAVLQKARAGDFANDPNLKTHIKCISEKIGFQGTDGKFRRDVIEKKLKETIPGDNAKNAKLIETCVVANKDPKLQAFNAFKCLYTNAKINLL

>DponOBP31

MTFQGGVSVFLCILGVAQLVAAGNSDDLFARIAPADVEMCGKDTGVDRKEFEDAREKRALNHSMLCFLKCAMEKVGFLKDGHLEIDQAKGSLPDKMMEPVVECFKAVGPISTCDDIQKVEDCLPSS

>DponOBP32

MNQGCFLLVVSAVLVFAELDQTSLPPETKELMAALHKNCIEQIGVSEADVDQLRAANFEEDAKLKCYTRCLMAESGVMDENGAIDVEAFAEILPEDIRGNIQTIFRRCSLTNKDIEDQCVKAYEMVKCWHKEDPESYFMI
