# Supplementary material for: Antennal Transcriptome Analysis of Odorant Reception Genes in the Red Turpentine Beetle (RTB), Dendroctonus valens
Source: PLoS One. 2015 May 4;10(5):e0125159. doi: 10.1371/journal.pone.0125159 (PMC4418697; doi:10.1371/journal.pone.0125159)
Supplement: S2 Fig — (DOCX) [file pone.0125159.s002.docx]

**S2 Fig. Amino acid sequences of OBPs used for Multiple sequence alignment**

>DvalOBP1

RLTEKQLAAAIKLVRNMCMGKSKANPEDIEKMHQGNWDVDYQAQCYMWCGFNMYKMLDKENHFDKKAALQQMDQLPIDLQEYVVKCMDQCENAVTNFDDKCVVAFEYSKCLYFCDPEKYFLP

>DvalOBP3

LKITLPPELQEYVDDLHKLCLEKGGLTENDHQTYDINHKNEKMMCYMKCLMLESKWMKSGGEIDYDFIETQAYPEVKDLLLNALNKCRTIEEGADLCEKSYNFNKCLYDADPVNWFFV

>DvalOBP4

LSDEMQELANQLHTTCIGETGAAEDAITNARNGDFSEADSFKCYIKCLLSQMAIIDDNDGTIDVDAMVAVLPEEIQEATEPIIRKCGSIIGANPCDSAWLTHKCYYKEGPEHYFLI

>DvalOBP12

GKPNDLFTRITPGDVEVCGKDTGVDRKDFEEAREKGALNHSMLCFLKCAMEKAGFLKDGHLEIDQAKEASPDKMTEPVVECFKAVGPISTCDDIQKVENCLPGS

>DvalOBP13

QDFTEEQRKKIIENRQQCIEETKVNPDLIEKADLGDFAEDQALKCFTKCFYQKAGFVNDKGEVQKDVVEAKLPPQADKKRALEIVDKCALKGKDACETVYLIHKCYFEHTHPEADEKTAKDGKSEEKKA

>DvalOBP14

LDQSWRDHMKEKLTEFGIECAESEQATSEDIEALHNHKPPVTHAGRCVIFCVSKKLNLMNPDGTLNVTPQSDWIEKVKESDSEAFEKMKTVYHHCADTVEVEADACDTSLSYAHCIKEEGHKVGLYTVSAD

>DvalOBP15

YVPNVNDKIRDFCIDDSGVSIEMVENLLANPEKELIDVESCYVHCIFTEMGLLSENGNVEIENFKSLKASEAPYIDLNCLEEIKSIDHCNEMMILRACHV

>DvalOBP16

AMTEAQMKAALKLIRNVCQPKNKATDAQIAAMHNGDWNQDKNGMCYMNCVLNYYKLQLPDNSFDWETGLKVVESQAPPSMAGFIMETIKSCKDAVKTGDDKCKAALEITKCLYDQNPEKYFLP

>DvalOBP17

ELDQTSLPPEAKELMAALHKNCIEQVGVSEADVDKLRAANFEEDANLKCYTRCLMAESGVMDENGAIDIEAFGEILPEAIRGNIQAIFRSCSLTKNDIVDQCVKAYEMVKCWHKENPESYFMI

>DvalOBP21

LSDEMKELAQMLHNTCVAETGVNEDFIQKVNAEKIFADDENLKCYIKCLMAQMACIDDDGIIDEEATIAVLPEEYQALAAPVIRACGTKHGANPCENAWLSHRCYAEMEPSAYMLI

>PjapPBP

MSEEMEELAKQLHDDCVSQTGVDEAHITTVKDQKGFPDDEKFKCYLKCLMTEMAIVGDDGVVDVEAAVGVLPDELKAKAEPIMRKCGFKPGANPCDNVYQTHKCYYETDAQSYMIV

>PdivOBP1

EEMEELAKQLHNDCVGQTGVDEAHITTVKDQKGFPDDEKFKCYLKCLMTEMAIVGDDGIVDIEAAVGVLPDELKAKAEPVMRKCGFKPGANPCDNVYQTHKCYFETDPQSYMIV

>AcupPBP1

MSEEMEELAKQLHNDCVGQTGVDEAHITTVKDQKGFPDDEKFKCYLKCLMTEMAIVGDDGIVDIEAAVGVLPDELKAKAEPVMRKCGFKPGANPCDNVYQTHKCYFETDPHSYMIV

>HpicOBP1

EEMEELAKQLHDDCVGQTGVDEAHIGTVKDQKGFPDDEKFKCYLKCLMTEMAIVGDDGVVDVEAAVGVLPDEFKDKAEPIMRKCGVKPGANPCDNVYQTHKCYYDADPNSYMIV

>ApOBP3

LENYPPPEVLEYLKPYHTICTEKIGVSDDEVKNYKIEDNSEKMMCYMRCLGLESKWLTPDNKLQIDYIMETRLDSIADLVKNIVDNCKDVPDGTHECEKAYNLHKCAAKIEPERWFLP

>TcasOBP1

ILEDSELMKVVENCVKKTNANESEFSSPNFLETTPSQPALCTAKCLLESLEIVNSEGNINMETLKEYAQPFESPAREAVATCGEEIKSVTTCDDMEKYRKCVEPLIKNS
