# Supplementary material for: Antennal Transcriptome Analysis of Odorant Reception Genes in the Red Turpentine Beetle (RTB), Dendroctonus valens
Source: PLoS One. 2015 May 4;10(5):e0125159. doi: 10.1371/journal.pone.0125159 (PMC4418697; doi:10.1371/journal.pone.0125159)
Supplement: S3 Fig — (DOCX) [file pone.0125159.s003.docx]

**S3 Fig. Amino acid sequences of CSPs used in phylogenetic analyses**

>DvalCSP4

MLLIISVLIGMALDLTDAKPAAKNYASKYDHIDVGAILNNRRMVNYYSACLLSQGACPPEGVELKRILPEALQTNCARCSEKQATIALMAIKRLKKEYPKIWSELSAKWDPSDSFVKKFETTFESLHGPGRRVESTTSAGNKLDPSEADGNTIDANITQTSPEGSDRVNQPDTTTTPQIITTNPSFSTSTKPAFSSTKRPSPIPGLVPFNTFFTNPPIPIRPIVNLNLGGNIGATVKAIKQVEKMVADIALEKIGIIRSILRPWRKAKKTRYA-

>DvalCSP1

MVPLAGSFVLIAVLTLLIGEENGANARSVKRSAQTYTTKYDNIDIDQILASNRLLKNYVNCLLDKGACTQDGKELKKYLPDAIATECSKCSQAQKKIAGRVLQALLLNHRDDWELLTNKYDPDGNFQKKYLQEDEDYSDLEEA-

>DvalCSP6

MALFPQYWLQFGALLLLLALADGQVLNGNVYVEKQLLCALDRAPCDNLGRQIKDALPEIIGKNCKACDNKQLSNAKRIARFVQNKYPDVWNDLVRKYGNPTN->DvalCSP3

MYAISAEIRAKRGQFPYQKRPRPMEEDYEEIRS-

>DvalCSP5

MAIDHKIKCKSVIMKICILVCCAFIGLVLADTPKYTTKYDNVDLDEIIKSDRLMKNYVNCLLEKGKCTPDGAELKRVLPDALHTECSKCSESQKKGSRKIMRHLIDNKPEWWTELESKYDKEGAYKKQYREELKKDGIKL-

>DvalCSP2

MKVVLLLVVVVGVAYGEEYTSKFDNVDLDQILSSDRLLGNYMNCLLDKGKCTPDGTELKKNLPDALENDCSKCSAKQRDGAKKVIRYLIDNKRDYWDQVAAKYDPEGKYYKKYQEEAKKENIKL-

>DmelCSP2

MKASLALVFCVCVGLAAAAPEKTYTNKYDSVNVDEVLGNNRVLGNYLKCLMDKGPCTAEGRELKRLLPDALHSDCSKCTEVQRKNSQKVINYLRANKAGEWKLLLNKYDPQGIYRAKHEGH

>DmelCSP1

MLLLNKNRVISLVVNFIFLIILISSSVQADERNINKLLNNQVVVSRQIMCILGKSECDQLGLQLKAALPEVITRKCRNCSPQQAQKAQKLTTFLQTRYPDVWAMLLRKYDSA

>ItypCSP1

MKLIISFLLIAVAALSYADKYTSKYDDVDIDQILQSERLLRNYLNCLLDKGRCTPDGAELKKNLPDALENECSKCNESQXKGASKVIRYLIDNKRQYWDELAAKYDPEGVFFKKYEAEAKKDLLDQIGRA

>ItypCSP2

MGGHRKSYLVLALVLVNLVSLNRAAESTTRAPISDDALEKTLSDKRYLTRQLKCALGEAPCDPVGRRLKS

>ItypCSP4

MALLIFFVVILTVGLASAKPAVKHYASKYDHIDVETILNNPRMVKYYSACLLSQGPCPPEGVEFKRILPEALHTNCHRCTEKQATVTLRAIKRLKKEYPKIWSQLSQMWDPDDVYVRKFESTFGNRNKIPSVVVNNGWDLGSSTTSNADEPRPDTTTHQIITSPNIMSFTTSKTSSTPITTSSTANPSTKTSTTTVGTTTKPPSRPAPIPGLLP

>ItypCSP5

MQCLGLFVVLVLGCSLVAAQSPYTSKYDNVDVDKILKNERVLTNYIKCLMEEGPCTPEGRELRKTLPDALASGCSKCNEKQKDTTEKVIRHLMDKRTKDWDRLSKKYDPQGVYKQRFEKELSARKLA

>ItypCSP6

LIGLAPLVLRGSCPQCTEQEKKQIKKVLAYVQVNFPKEWNKMLQTYASG

>DponCSP1

MKVVLLLVVVVGVAFGEEYTSKFDNVDLDQILSSDRLLRNYINCLLEKGKCTPDGTELKKNLPDALENECSKCTPKQRDGAKKVIRYLIENKRDYWDEVAAKYDPEGTYYKKYQEQAKKENIKL

>DponCSP2

MKFCVVLVLVLQIAICLGQTYTSRFDNINIDEILSNKRVLNNYVRCVLDEGPCTAEGRELRTHIPEALRTSCAKCTPSQQKFVRKGANFLIKNDPDQWKRIAKKFDPEGKFAPQFRQFLNA

>DponCSP3

MWKLVLLGSLLICIGQTLAEVTEKSQYTTKYDNVDINEVVHNERLLKNYVNCLLDRGPCSPDGLELKKNMPDAIETDCSKCSDKQREGLEAMMRFLIDNKPEYWNPLQEKYDPTGSYKKRYLDAKRAEVAIQPAEKTP

>DponCSP4

MHCASVFFVVSALLVLISAQSSPYTSKYDNVDVDKILKNDRVLTNYIKCLMEEGPCTPEGRELRKTLPDALASGCSKCNEKQKSTTEKVIRHLQTRRAKDWDRLSKKYDPEGVYKQKYTAELKTETTA

>DponCSP5

MWKLVLLGSLLICIGQTLAEVTEKSQYTTKYDNVDINEVVHNERLLKNYVNCLLDRGPCSPDGLELKKNMPDAIETDCSKCSDKQREGSEAMMRFLIDNKPEYWNPLQEKYDPTGSYKKRYLDAKKAEVAIQPAEKTP

>DponCSP6

MKTIIFLVVVASFYGLSSCKPQEKYTTKYDNIDLDAIIRNDRLLRNYIDCVLGKKKCTKDGEELKVHLPDALQSDCSKCSEAQRNGSRKIITHLLKNKRGWFNELQAKYDPAGNYLSKYSEELRKEGIVI

>DponCSP7

MVPPGSFVLIAVLTVLMMDEENGANARRVKRSAQTYTTKYDNIDIDQILASNRLLKNYVNCLLDKGGCTQEGKELKKYLPDAIATECSKCSQTQKKIAGRVFQALLLNHRDDWELLTNKYDPEGNFQKKYLQEDEDYSDLEEA

>DponCSP8

MKIFIVVCCAFIGLVLADTPKYTTKYDNVDLEEIIKSDRLMKNYVNCLLEKGKCTPDGAELKRVLPDALHTECSKCSDSQKKGSRKIMRHLIDNKPEWWTELENKYDKEGAYKKQYREELKKDGIKL

>DponCSP9

MNSHCFQLQLIALLVLVALVSLVRNETTERPAISDEALEKTLSDKRYLQRQLKCAVGEAPCDPVGRRLKSLAPLVLRGSCPQCTEQEKKQIKKVLAYVQVNFPKEWNKMLQTYAG

>DponCSP10

MKVVLLLVVVVGVAFGEEYTSKFDNVDLDQILSSDRLLRNYINCLLDKGKCTPDGIELKKNLPDALENECSKCTPKQRDGAKKVIRYLIENKRDYWDEVAAKYDPEGTYYKKYQEQAKKENIKL

>DponCSP11

MAPFPQSWLQFGALLLLLALVQGQILNGNVYVEKQLLCALDRAPCDNLGRQIKDALPEIIGKNCKACDNKQLSNAKRIARFVQNKYPNVWNDLVRKYGNPTN

>TcasCSP20

MRFFVIFFVACVSVALARPEDQYTIKYDNVNLKEILQSDRLTENYVNCLLEKKPCTPDGEELKRVLPDALKTSCAKCTDKQKQGAKTVIQHLYKNKQDWWKQLEAKYDPEHTYVKAHEDELKAL

>TcasCSP19

MKFFIAFLMLLGAVWCEQYTTKYDNINVDEILASERLLKNYFNCIMDRGACTPDADELKRVLPDALKSDCAKCSEKQKEMTKKVIHFLSHNKQQMWKELTAKYDPDGIYFEKYKDKFDS

>TcasCSP18

MLFTVFLVLTCAHVVFLEEYVIPDNIDIDDILSNERLLKNYVNCLLDKGRCTPEGKKLKSTIPEALSTDCAKCNEKVKANVRKVLHHLIDNKPDMWKQLEAKYDPSGEYRSKYKDELEKNGIHV

>TcasCSP17

MFKVLFVVFACVQAYVYAEEYTVPQNIDIDEILKNDRLTKNYLDCILEKGKCTPEGEELKKDIPDALQNECAKCNEKHKEGVRKVIRHLIKNKPSWWQELQEKYDPKGEYKSRYNHFLEEEGLN

>TcasCSP16

MTAIVFLLALACLKTYVSSQEYLVPQNIDVDEILKNDRLTRNYLDCVLGKGKCTPEGEELKKDIPEALQNGCAKCNEKHKEGVRKVIHHLIENKPNWWQELESKFDPQGEYKKKYDELLKKEGLAN

>TcasCSP15

MIFKIHFLVFGALLTYVSSVEYLILREIDTILKNDQMTRNYLDCVLDKGKCTKEAEKLKKGITETMKNGCVKCEQKQKEDVHKVFQHLMIHRPNWWHELETKFNPHHEIKLQHLHQSKFNPHEEVKLQHLHQFPHHDFLEREGFIR

>TcasCSP14

MFATSALFAFICIQGLVSAEEYLVPQNIDLDEILKNDRLTRNYIDCILGKGKCTPEGEELKRDIPEALQNECAKCNEKHKEGVRKVLHHLIKNKPNWWQELEAKFDPKGEYKQKYNKLLEKEGLQA

>TcasCSP13

MFLAIVLVVCACTNVLSEEYTNQYNDELDAALKSERLMKSYFECLLGTGKCTPSGEELKKDIPDALKNECAKCNDKHKEGIRKVIHYLVKQKPEWWEQLQKKFDPQGIYKKRYQNYLDKEGLKA

>TcasCSP12

MKTLVLVLFVAVLSVVFAADKYTTKYDNIDLNQILKSDRLLKNYVNCLLDRGKCSPDGQELKNNLADALQTSCSKCSQRQKDGSRTIIRYLIKNKRDWWNELEAKYDPTGIYKNKYADELKAEGIVL

>TcasCSP11

MKTLVPLLFFVIAIASSLAENSKYTTKYDNVDLDEIIKSDRLLKNYVNCLLEKGKCTPDGAELKRHLPDALHTECSKCSETQKNGSKKIMRHLIDHKRDWWNELEEKYDKEGEYRKKYEAEIKGKKD

>TcasCSP10

MKTFVLVAFAAVLGLALARPQEKYTTKYDNIDLEEILKSDRLLKNYFNCLMERGTCSPDGEELKKALPDALHSGCSKCTEKQKEGSRKIIHYLIDNKRDWWNELEAKYDKDGVYRQKYKDVIEKEGIKL

>TcasCSP9

MTAIVFLLALACLKTYVSSQEYLVPQNIDVDEILKNDRLTRNYLDCVLGKGKCTPEGEELKKDIPEALQNGCAKCNEKHKEGVRKVIHHLIENKPNWWQELESKFDPQGEYKKKYDELLKKEGLAN

>TcasCSP8

MPLVKSLVVVVLLIGVVYQVQGQLGLAGNNYIEKQLLCALDKAPCDALGNQIKGALPEIIGKNCERCDSRQVANARRIARYVQTKHPDVWNALVKKYSV

>TcasCSP7

MKLISAVILCAFLVAVSAAENKYTNKYDNVDVDKILNNDRVLTNYIKCLMDEGPCTSEGRELKKTLPDALSSGCTKCNQKQKETAEKVIRHLTQKRARDWERLSKKYDPQGQYKKRYEEHVATSRAA

>TcasCSP 6

MIPLIAIAGILAVSAAPAEFYESRYDHLDVESILNNRRMVNYYAACLLSKGPCPPQGVDLKRVLPEALQTNCAKCTEKQRTAAYRSIKRLKKEYPKIWEQLRAVWDPDDVFIRKFETSFESGKPSGVISTNTSPPSPILSNRFGENEEADAASNVISSTPLPPTTSTTTRTTLTTKFTTKPSTKPTNKPVVVTKPPQAPPFATVGANLQATVSFGTNLVGGIVRSLGTLGSRVVESGTKLANMVISAAIRP

>TcasCSP5

MKTFVILFFGVFFIIFSDFVNGKTLHRSTRDDKYTTRYDNVDVDRILHSKRLLLNYINCLLEKGPCSPEGRELKKILPDALVTNCSKCSEVQKKQAGKILTFVLLNYRNEWNQLVAKYDPDGIYRKQYEIDDDYDYSELDSAKK

>TcasCSP 4

MYSYLIPLYLFLFVHYGWSEDTTHKYTTKYDNIDLENVVKNERLLKSYVDCLLEKGRCSPDGLELKKNMPDAIETDCSKCSEKQKEGSDFIMRYLIDNKPDYWKALEAKYDPDGTYKKRYFESQKDEVSKVEA

>TcasCSP3

MLFTVFLVLTCAHVVFLEEYVIPDNIDIDDILSNERLLKNYVNCLLDKGRCTPEGKKLKSTIPEALSTDCAKCNEKVKANVRKVLHHLIDNKPDMWKQLEAKYDPSGEYRSKYKDELEKNGIHV

>TcasCSP2

MKIIILAVLIATAVAATYDVYPTKYDNVDIDAILHNKRLFDNYLQCLLKKGKCNEEAAILRDVIPDALITGCRKCNDHQKVSVEKVIRFLIKERNSDWQQLISVYDPKGEYQTQYAHYLEKI

>TcasCSP1

MLILQIAHLCAQFCLLAAIFTCVKPQLTRISDEAIESTLNDRRYLLRQLKCATGEAPCDPVGRRLKSLAPLVLRGSCPQCTPQEMKQIQKVLAFVQKNYPKEWNKILHQYAG

>AplaCSP1

KSFAIVTLLAVAMAVVYAAPDGVKFTTKYDNIDLDEILPKRETFQQLLQASNRRGKCTPDGEELKKAFLRP

>AplaCSP3

SRIVCTTEWKIHHQVRQCRLGSNPQQPKATRKLLQLPYGQRKVYSRWTRTEKNLPDALNTKCSKCSEKQKEGTHKVVEYLIKNKNDWWKNLESKYDPSGNYRRDYGPELAQRGIKI

>AplaCSP4

MRSAIVSLFLVFTIFTFNSLGDASDYSKRYEALDLDSILSNERILKNYINCFLDKGPCSPEARDFKKDIPVFIKSKCGDCTDVQRKVVLKASKFLIEKKPDEWKLLLSKYDPSGEYEAFLLGELKKQNS

>AplaCSP10

MSATKYTTKYDNIDLDEILKSDRLLGNYVKCLMEEGKCTPDGAELKKVLPDALKHKCDGCSDKQKQGSKKVVNFLIKNKQDWWKKLEKKYDPEGQYVKDYKDELEKEGIKL
