# Supplementary material for: Antennal Transcriptome Analysis of Odorant Reception Genes in the Red Turpentine Beetle (RTB), Dendroctonus valens
Source: PLoS One. 2015 May 4;10(5):e0125159. doi: 10.1371/journal.pone.0125159 (PMC4418697; doi:10.1371/journal.pone.0125159)
Supplement: S4 Fig — (DOCX) [file pone.0125159.s004.docx]

**S4 Fig. Amino acid sequences of ORs and GRs used in phylogenetic analyses**

>DvalOR6

MMFLAHFHFDQNVHYKRALLLQTITVLLSIIYFCLCPILYLGVLGFIRAELQVLQYRFRNFDSCKRSVSDVEMMKQLIQTHQFIIWFVKDFNESVRSLILIEFLISSVNIACVAFQLISANRITDDIFTPCFLFVLFGQLLVLAWPANEMSMEASNVAFNWRKYTTVFQSVGVSIAIYDFPWYEKSIPIQNMARVVLMRAQSPLKLTIGMFNPLTTDTVIKVDFFFRGFATGTEAIGFRC-

>DvalOR1

MINKFKVVGLVADLMPNIRLIQASGHFMFNYYADNSGSLHILRLGYCCMHLFLVLVQYGCIFGNLVKEKDNVSHLAANTITILFFTHCLTKFIYFAARSKLFYRTLGIWNQANSHPIFVESSNRYHALALKKMRNLLYIILVGTLFSASAWTGITFVGESVHFIKDPDNDNETITEEIPRLLIKSWYPFDAMSGMTYYAALVFQIYYVFFSLLQANLLDNLFCSWLIFACEQLQHLKEIMKPLMELSATLDTFVPKSADL

>DvalOR2

MLYPVRKGLPFYPNLLILKLAGYYPKSSNYNKKLFFYCLFCWMLIWTGTSWNLIILLYLSIQNKTNYGFVEAMGYLLGITSFTLACLHFALKHEDWSHLMDDLMDFQKYGKPPKFNKVQTSASKLGIAFTTGFFSAAVIYAILQVLLEEKCEKLSFGNTSCGMLLPTWFPASYAENKLIKRLVLIYQVLACCTMAPYTMIVSLVLEANEFIAYRIDHLKSQLRKVGCNGDSDLFLASVQYHHDIIRQVGQTFSIYMDLCAHCVF-

>DvalOR3

MMAPHVQFPLNRHKYFMETYVLSIGTTLGGVIYYTLTQVYFVVVTAFVISQLKIIQRLAREFHLYSNGGDQEKEALTVFRKIYHKHLYIIDFVGELNVLMKYLIFAECTVISIIIASVLFQLIFVPTSLAASTLYIAVSCTVLSQIFVLSWISNEIEVESLSISDALFESRWYEQTQKVKKIIIIIMMRSRKPLRIMIGPFYPLTIHTALNSLRAAYSYVTLIFAMSSSGQLQI-

>DvalOR4

MAGMVMVVICYVCAPAFRDPIEIQKENETIRIRQLPVSAWSPVEEYFLLDFVWKSLVGAYLAYFFVTTDLILYSFIAFGACQVRILQHYIRNFNRYCEEIMHTQGCSKNESAKVLQKQLIAMHQDVISYVNMINGSIKQLMMLEFLPGSVQLAGMLYQLMTNLNAIQCIFLGQFISCLIARIYIYTNSANDLSQLSQQLAVDWFEIDWIELPKDIKMNLNICIIRCQKNLCITVGDLNAIDMTTFLTILKGSYSFLTLLTTI-

>DvalOR5

MYGSLRLKVLRIGMQRFHHSPCGGVNMKSFVVEHLDSIRYIDDLNEATKSILFITFLLNSIKVASVLFPLMAVKSLSALAFPAIFSSMLVAEVFYLGWICNEVKEQSLKISTDAYKILWYNENKDVSVLLQMMIRRAQRPLTMQMGPFGPMVTDTILSTMKAAYSYTTLMLNARE-

>DvalOR12

MNIFMVVDFAQSSLLLTSVFAQLLWVEPSITFYGFVFMYVTYLNQRLFMNYYYSNEVWLLSENLNLSVWKCNWYEQSRYVKFMIHFFIMRTRKSLKFKIGPFGFMNLSTYLAILKASYSYIALLHSTKK-

>DvalOR15

MKPSKTKKAQLISWTKIFMIGGGFWNQPLSKSYIGEKVYFCYSIFMKCGCLMWWSMMVAELLRFVAYGYTLDVILAQFGLVINASKIMFKLIVYIKENLLALFKDITEKDIEIWNLDNEEIHTIYRKNIKLIKSYVLALSVSTSLCLGMLDVSGIHIILKTVEHNRAFNDTLEAHAMYETILPLNKLDNLPLLFTWQAYLAVIGLLYNCLTHLMFATLLVYAATQIQILQIRSKNFIGTEQLSDSDMKDKLLVLKDISKDHQYIIGFVENLNSRTRYIVLVEFILSSFDLASVSVNLITLDFSSNDIAGQLIFNLSFFVLLSIQISILGWSCNEIKCESEELANALYASNWYLLNPKGQKMMQIMMARAQKPLIMTIGPFGAMTTTSVLAILKGAYSYVSIMKK-

>DvalOR16

MEIVMVFDFLQSSLHIASILPEVLGSEISVMMVLSVVSFLASMLFRLILYYYHADKVILLSAELSYSIYESNWFDQAPKVKQMILIFILRTQRPLTLRIGGFGVMSIQSLIAILKATYSYMMLMI-

>DvalOR17

MSVFEETTQIRKKWRSSIAITEKVLVITEIWPSDDTSLLRTIKILFITIACIVFNITVIDELKMLSIRQDYKSLSMHLTTFGLYIGFSVKIMLFQLTKHGPLKKMLDSMDSPIFHAYPPEMQKYQDNCIRVSNLIGKFFVYLVGGTILFYLSKPLYSPYPLPVTFSHPLTTTTFYMLLTLQCFCSYYFIMIGICFDMLVMGLANVATAQLDMLIEEITTFTPKNKEPLEKEEHRFIKRCAQRHNAIIRYINSIEDVFTYIFLA

>DvalOR21

MIEFGLSTFDMASGSVGVIKMKGYESLWLLFFMMVLLTQLFLIGWTANEITVQSEAIAAALYQSNWYELNKEGRQLILISMIRAQRPLHITIGPMGPMTTGSILTVLKGAYSYINLMR-

>DvalOR22

MGAVKVIFWYFYGHLLRDIMNALENPRLHYEGYMDFSPNRISHLYKAIGRRYSLLFLSLAHATLISSYIPPLMAVAEYLTQPEGGIQQLPSRLPYFCWMPFSYDTPGKYLLAVAYQAGPMFSYAYSVVGMDALFMNILNCIAENLVLIQGAFRTVRERSSLVPHWDGIRESPLVRRQMDLEMKKIIKHLQITLRACKHVEGIYHVITLSQVTATLFILCTSLYLVSTASPFSKQFFAELVYMMAMLFELFLYCWFGNEVTLKASY-

>DvalGR1

MTNIIISVYGFTSEVVDHGVKFTFKEMGLLVDAIYCFTLLYIFCDCSHQASANIAERVQWALMEINLNQVDHATIKEIQMFLKAIHLNPPKVSLRGYTVVSRELVTAMISTIAIYLIVLLQFKISLVNMRG-

>DvalGR3

MITGRILDLVIGALIFASLTESLPAQLKERNLLYSDPGVVAVYIRPGDTPLQDINPDLAEAFNFNDVKYGRRAFGRDINKILNEKKENSVLFSSGELAEGESYAPKKKSLKERGESHIQKIPKH-

>DvalGR4

LVLKNKFHDINSLLSSYEADQSTTLIKGTQKVKDLYSSMTDVVGRFNELFGPQLFLMGLHCSSQILQWSVSLVYSMFTTKDDNVNDYARLAVGIIYIFLMLIWLGITMFCCDMAVLESEEIITICYRIQLKYSVFSEAYQSIKNLLYLVTNTKIRFTAMDYYEINRSTMFDLIGTTATYFVVLIQFYDGNKK

>TcasOR1

MMKFKVTGLVADLMPNIRLIQASGHFMLNYHADNSGALHTLRLGYCCMHLVFVLVQTFSCNFVNLVLERGDVNDLAANTITVLFFTHCVTKFVYFAVRSKLFYRTLGIWNQPNSHPLFVESNNRYHGIALKKMRRLLYIIIIWTSFSAIAWTGITFVGDSVHNIKDPENENLTITEPIPRLLVKAWYPWDAMSGMPYYITLVFQVYYVFFSLAHANLLDSLFCSWLIFACEQLQHLKEIMKPLMELSATLDTYVPKSADLFRAPSATSQDQLIENGTNPAKKNEDLKGVYSTRQELGGHFRGGALQNFGSGGVGPNGLTKKQELMVRSAIKYWVERHKHVVRLVTAIGDAYGVALLLHMLTSTIMLTLLAYQATKITGVDKYAATVLGYLLFALAQVFHFCIFGNRLIEESSSVMEAAYSCHWYDGSEEAKTFVQIVCQQCQKAMSISGAKFFTISLDLFASVLGAVVTYFMVLVQLK

>TcasOR3

MKLSSVTTCLFSSDFHTRMNFDWKDTIKLNFLMMKIVGLWPKEKYKINFYTLYTLISVNLFICGHVIFHTVAVFVVGRDLKHLIGALYMSLTETLLLVKICYFIKNSRLVKSLLTSLDGDIFQPKNEKQLELTNPSLIFWKKVHKSFAILVANTVFLFVSLPILSKSTKLYRLPLEAWYPYNTQKSPNYEITYLYQFISTLFRGMASVSMDTFIAALNMYIGVQCDILCDNLRNLNETNFMENLSLCIKHHKAIVSFARECNKFYNGIVLGQFFSTSIALGLAMFLLSLVTPLSTESNTLLFYLGATTSEIFLYCWFGNEVDVKSSKIPYSAFESDWTGAPIEAKKNLLIFILRTQKPIKMSAINLFSLSLETFTTILRTSWSYFAVLRQVNGQA

>TcasOR7

MNKLQKFDWKATIRPNIAFLHYLGIWPEGEEYYKLNFYTLKTILYIIILVISTIVFQVINIFFTLDDLTSLTANIYVLLTEILYFIKLCFLVKNMPALKLLMKTLDHKLFQPKANQIVIIQPLLNFWKLIFLAFVITCSFTVLFWAIFPILDSSEEEKRLPLLAWYPYDTKISPNYELTYLHQVASYIYICYSHLNIDTFITALNTYIQCQFDILCDNLKNIKSDTKNVDTKLAKCIKHHLLILMFANTSNEFFSWIIFFQFTSSAAITGMTLFQLTVVKPFTTEFYNFMAYVTAEVVQIFMYCWFGNEVQVKSSNIPYAAFGSDWTEFSPNKQKSLLFLITRSQKSVKMSAFNVFDLTTDSFILKSAWSYFALLNQVNS

>TcasOR17

MDDFNWISTVKTNLLLLHIGGIWPRGDGTHKLNLYTIYAIFITFTFTTYHCFSQIINFFFVDDLQALTESIFISLIQSMALVKAFYILKNMRILKNILKNLETNKMLQPRNLKQIKMVQPSLTQWRLLSQMFWISAVFAMCLFGAFPIVESTYKEFRLPYLAWYPFDTKSSPFYEIMYLHQFVSSYTIAIVDIGADTLIAALNVFVATQCEILCDNIRNINGSVEEMDSKWKECFTHHKEILKVARHCQKFFNWIVLMQFCASVICIGLTMFQLTLVVSFSSEFFSSLFYFGAITVQIFMYCWFGNEVELKSSKILYATFEANWVEAPHQVKKNILIFAIRCQNPIKMSSLNVFYLTLETFMAIFRTSWSYFAVLRQIQNRISEE

>TcasOR20

MNSFNWQESIKTNLKALRLVGLWPKSDFYKFDLYTFCTSLTVGVIVCGHNLSQIVYILQVYSDLKALTATIFVASINFLGAVKMYFFIKHIKTVKILFKMLKTYQFKPKNIHQTQLIKPFLNLWKILYVGYSINVYLIVAMWSLLPVLNGWTWQKKLPFPARYPFDVTKSPYYELAYVYQFICIWYITVANLNLDTINIALMMYTSCQCDLLCDDLKNLTETRFFHKKLIECIKHHKAILVFAEKSNGLFNMIVLSQIATSTVVLALTMFQLSMVSPLSSEGLNHLFYIGGIIMQILLYCWFGNEVEAKSSNILYAIYESTWFEASKNSKKNLLIFSIRCQRPIKATAVKLFALSLRTFITIVRSGWSYFAVLYNVGSK

>TcasOR24

MEEDFDFLSSLQTTLFCLRCVGTWPSNTYKLDAYTLYATASITICLFGHNFFQTVNIFFIFNDLNTLTGVIFVALTCLVAILKSLLFIFNMRRLKKLLLVDIRQKLFKPRNRQQVVMVQSRVNFWKKIYFMFTGMGVATMFFWALFPIMDGTVKEHRLPFLAWYPFSVNKSPFYEITYIYQIVSVFFIVIVNMNSDMLLVALMNILGVQCDLLCDNLKNIQFRERINEEFLRCVNHHMQILSYASDCNKFFNTIVLAQFFTTVVSLGLTMYQLTIVTPFTSEFYSFIVYGGAVLMEIFLYCWFGNEVEFKSLNIPFASFGFDWTIGSVGLQKNLIIFIAKSQRPIRMSALNLFHLSLETFVKILRTAYSYFALLNNVNSLN

>TcasOR46

MSKSEKIHTLATYFDSNIAFLKLTAFWIYDDETTRRKKYLQHAYNIFWIFYLFVAYQPAELLYVYYSFNDLSVFLRALRDIGNHVSLAYKAFNYFIMRRDILKLMETLQHGNYHYEDCGDFQPKLIVDEEKKEALKWTKYFLNFCNAICLSMFANGVFTFIFLSDKQYVERNGQRVYHQEQPVNTVSPFGSGTKLRFFVTFIYTMIALTFYAWTIVALDSLFITIMSCISSHLKILQGAFKTVRARFIKLCASLSKLLISVSGKLESIYSTQTFVQTFISLGEMCFSLYLLSETADQNIGNEITYLIATGFELLMYCWFGNRITEASLKISYALYESDWFPTSLSFKKQIIFTMTRMQKPINVTIGKITPLAFSTFLTIARGAYSFFTFLKQRHGINH

>TcasOR58

MPFTIKDYDLRNAFETERTLLTLSGFYPRRTKKYNFFYNTSALINLFIAYGQLFSMVVQMVIDRNELSKLSETLLFFMTHFTFLCKLTNFVYYKKKMFEIEDNLSRKIFYGFELWQIKPKIDSCKFIAKIFRILCILVVLFYTLVPYLDDKEDLSLPLPGWLPYNTKKYYYPTVIFQVMSVSVSAYNNSSIDVLTCMLITVASAEFNLLKGALKTIDFHPKGHNTKQLIEAKFENCVNHHKEIVKFAYQIETIFSKGIFLQFFASIIVICFTGFQMIVVPIPSMQFIFLIIYFSCMMCQVAMYCWYGHDIITTSDSIGQAFYMSNWYESDVKIRKNICIFLERTKKPVILTAGKFVTLSLTTFTTILRSSYSYFAVLQHLYKEDS

>TcasOR59

MDEEFLIGTFETEKKFLRYGSFYPCGKRIKFIFLGLFMFVYSWTEFLSMITVLFVERDNLTKLSETLLFCMTQAAFLFKLVNFLYHNKTMLRIESILKNPILNCLDQFEKNIIEKYMIRVKYLARLFRILCILTVSFYGLFPFIDEDPDHMLPLPGWFPFDVKTHQIELVIAQTCGIAIGAFLNSTLDILPTILITLGSAQFDILKIRLENITSVDTSKSWLVKKAIKKCVIYHTILLNYITQIEILFHKGIFVQFTASVVVICLTGFQMLVISVRSIQFILLMIYFSTMTCQIALYCWYGNELMYRSMGLSDACYMSEWNKCDTSVCKSLAIIMERGKRPVVLKAGNIFSLKLTTLMTVLKSSYSYFAVLQRLYATSE

>TcasOR60

MSEDYTFRNVFAREKKILTISGFYPLREYEKNYFHFFSGTIQWIISLGMLFSMIIQSVIKRNDLMVLSETLYFLTTHLTFVCKLANLEYHKKLLLDIEDMLKTTRFQKTLSLDLIEKTGMNEKIRKFNLVAKTFRIVCVWCVVLYVLVPYFDPGKSKTLPTPGWFPFNWTDKYYYGTYFFEVAGISITAHMDSSIDILSWLLVTIASFQCDILKENLKNIYYNYDKEHDIRETFKDCIRHHEEIIKFTTKVEQSFSQGILLQFLCSALVICFTGFLMLVVPVLTFQFANTIMYFCCMMIQLGMYCWYGHEIMTTSDEIGQYFYLANWYDSSLTLRKDFAIFLERAKRPITLTAGGFVVLSLNTFTRILRSSYSYFAVLKHLYNKS

>TcasOR61

MGDYDFRAAFAFEKAIFSLSGYYQRQAGFSSLIICAIASLITIAQFLSMVMQIIVAGNDLTVLSETLLFFMTHFTYMCKLVNLLFYKSKLLHIEDLLSRPRFYGFSQNELTIIKDGIEATNTVANLFRIFCVLACIAYGLVPYLDHTKAMALPLPGWLPYDTTKYYYPTYFFQMVAVSITASVNSTIDILTWKLITIASVQFDILKRKLKDLDYKLETTSLQIQFKTCVKHHKEIVNYVKNVEKTFSKGIFIQFFASVIVICFAGFLIIITPVLSMQFLYLTLYFMCMISQVAIYCWYGHYVMTTSDEIGQDFYMSNWYESDVAFRKDIIIFMERVKKPVTFTAGNFITLSLVTLTRILRSSYSYVAVLQHLYNEV

>TcasOR63

MGFMIQDYDLRNAFSLERKLMLVVGFYPKRDNKHEILYWLSAFFNLLISYGQLTTMIIQMVFDRSDLSKLTESLLYFFTHFTFLCKLLNFQYYSKDLIEIENFLTDPIFYGYSFEQLDIIKAKIRSCAFISNAFRICCTFTCSFYCLVPFIDESRKKILPLPGWFPYDTTNYYYSTFFVQSLSLFISAYCNTAIDILTWKLITLASAQFEILKENLTKIDYEGGFNETKGALVRCITHHAKIVNYTERVEAIFSKGIFLQLFGSVIVICTTGFQLIVVPIPSVQFAVLGTYLCGMTTQVATYCYYGHEVMTTSDAIGMSLYLSNWYASHVKIRKIVMIFLEKTKKPTIVKAGNFITLSLATLTQILRSAYSYFAVLQRLYKDS

>TcasOR64

MMSDEYVKDVFIANRWMLRCAGLWTPSTRSKLVQIPYKIYAIVVFLFVNVYFTSTEFLSLFYTHKNLYNFIKNVNFFLTHFMGAVKVIFWFFKGHVLRDLMRTLESPEFHYEPCEGFQPGLIWRKYRRIGFKYSLGFLALAHMTLSSSYIPPLLTKLPYFSWMPFSYSTPRSYLLALGYQAGPMFSYAYSIVGMDTLFMNIMNFIAAHLVILQGAFASSKMRVLDPGQMNNEMKRNCRHLQTILRVSEDLERVHRYLTLGQLTATLFILCTSLYLISTTPASSKQFYAELVYMVAMGFQLYLYCWFGNEVTLMASEIPVNVWKADWYDCDQSFKKSMIFTMTRMQKPIYMTVGKFAPLTLQTFVYILRTSYSIFAVIKNTSI

>TcasOR65

MTATKSLKEIPPIYLRVHLTVLQILGIDILPVESVPQNLFYTYTALIISTMCLFTIAEFLDMVLNYEDIYRLTFGLCYCVTHVLGTVKMFLMLYLRKKLWGNLTTLEEGIFKPNPTRGGPEELQIVNDAITMCNRQGYVFYTLVFLIIGARLLYASLANWPYDKHNYFDGNVTVIVNTKEMPYTTWMPFDYNDSPLYETIFAFQIFSTTVYGFYIGAADAVICGFMMLIKAQFLIVKRELETLIERAQKAAIAENPDNEDNFGREIERIELLDKRTQDYVAKYANECVYHHQELIALCDHAEEDFCYLMLLQFISSLLIVCFQLFQVSTLSPDSVEFFSMVCYLLLMLFQLLCYCWHGNEVQIVSGELSRYAFGINWIIMRESPKKTLLLLMMRAQRPCYFTAGKFSLLSLQTFMTIVRGAGSYFMFLRQMNI

>TcasOR66

MSKNLKEIPPVYLKVHLTVLQILGIDILPNERIPQTLFYTYSVLLIATMVVFTTAECLDLVLNYEDIYKLTFGLCCCVTHVLGAAKMFLMLYLRKKLWGYFTTLENGIFKPNPCRGGAEEFEIVTSAINMCKRQGYVFYVLTVGVTGGQGLYAALANLPYDKHNYFDGNVTVVVNTKQMPYATWTPFDYNDSPLYEIMFAFQIFSTTLYGFYIGAADAVICGFLMLIKAQFLIVKRELETLVERAQRAGNPDRGDFGGGINRIEMLDDGTQVFVEKCANECVYHHQELIALCEHAEEDFCYLMLLQFISSLLIVCFQLFQLSTLSPGTFEFFSMACFLLFILFQLLCYCWHGNEVQFVSGELSRYAFSINWIIMRESPKKTLLLLMMRAQRPCYFTAGKFSLLSLQTFMTVVRGAGSYFMFLKQMNT

>TcasOR67

MDFTIRDFDLRNSFSLERKLLLVLGFYPIRDKEKHRILHQLSAFLNLLLYYGQLLTIIIQMVIDRNDLSKLTDSTLYFLTLFTFLCKLFNFQYYGKDLIEVEKSLTDPIFYGYSFHKLQIIKAKVRSCTLVCLAFRISCTCSCFIYSVVPFIDRSGQKTLSIPGWFPYDTAKHFYITFFLQSLSLFISAHCNSATDTLPCKLISLATAQFELLKDNLRTIDYENSFEETKHALVKCITHHRKIVNYTKRVETIFSKGIFLQLFASVLVICTTGFQLVIVPFGSLKFAIHGIYLCAMTAQIAIYCYYGHDVMITSDEIGTSLYMSNWYASHIKIRKIMVIFLEKTKKPTIVLAGNFITLSLVTLTQILRSAYSYFAVLRRLYADD

>TcasOR72

MAKLEYLTGATFTLKCAVLYPIDSNNPKIKKILYAVWAIFFILTFVTGFIQCFVFVCINPFDLVQEAMIIMSLVFYSTTFFYFIVFYKNWQNMVALVTNINKNFHRATDNVIEKISMDQASELSDKLAYVWTSSLAVGSVVPVVLAIATGNLEMPMPAWFPYDYNKSPVFEITYLWQVFCLITLAIIYGASDMFFPCITIIIGQQFKILASNFKNNFYTSLIKLGAEESIVQNFSKDIKTHEFRSFYIKYGNIFKILNNAKFQTLNRAFLKRNIKHHKLLLRFCEDLNKILNTFLLIRVSAIVFNLIFIGFNIIINADFLWTLYECPWYLCDVTYQKMLILVQMRVKRMVSTKAGNFFTMIAPSFIAFQRAVFSYITLLKEVTDLGKD

>TcasOR73FIX

MTRKHIFLNFTVTILKLSFLWPSNDNYDQWRLVKDASLIVSLMPCALPILAHFVLQITGDVYNMVTITENLIALICIIGMIYMTICFVKNRKLVKTLVKNLPAFTKYSKTTDIILTDKKANLYTKIFVFYGVIGNVVYMIMPYLNIEKCQQRQNNDVPCGLVTRCWFPFKFDYSPVFEIVFVHQFYTCLMVSVIILDLTMLICGFLMHITNQLKHLRGFIKRFDCSSQKIAEDVIYCVKFHTAIITYSEKTNEAFGTMMMLHITLTSLVISALGFEILIVDNFNDSLRFTLHLLGWLVLLLLICYYGQLLIDESIAVAEDIYYVPWHLAPVDVQKDIYMILMRSQKPLTLNAANIGVMSFPTFLRVISSAYSYFTLLLNIKS

>TcasOR76

MMESTVTRLKRMYLWPTASVTSRKPAFFLITFSCFLLYGSVMHLIVNDISMEEVHVIETTAGQFGVLYYLTLFTIYRKGILEIYADLSNFTKFGKPYNFDKRNKQLNQWSRWFSVVLYFFVISVFAWPGIFTQSCEDLNVALNKTEVCGVVSPVWLPFRFDYKPMKQFVYFWQSFCCLYSNGGAGTISFAMSETIEHLILRVEDLKILFPKIVAERSPEVRRKMLAKWVDYHLWLLSIGKLMNDTYRYSFSVIVLCAGTLFGCIGYTVMKNASTNFNSSFIFFGWMESVFVICVCGQRLMDAFHSVGTTVYNSEWCDTDVDFQKGVILITIRAQKPVRIYAGPFSYVSHLLILTVFQTSYSYINLLNASS

>TcasOR77

MKYILMKKTIAFLSVTGFWPKTKESTKTRAFCILFSSSFLLFGSLGYLIVYRKFGSDDIDSIETATSHFGVLYFMFFWILKRDGLVHIVNLLSDFSKFGEPRFFNDRNRQLDYLLQYCIFVLSVATGGVFLCPIIFVKNCEMVKQEKNLTKVCGLVSNVWAPFDYSEYPMKRVVSLWESYCCFINFGCGGIMSFTMIKTMEHLHIRVEQLKDMFPDVVNEKNLAVRKQKLEKWVKYHLHLYDIGELMNNTYRYCLSVIVLCVGILFGCIGISTMQPGSSHNSLFLFMGWFQSICILCMVGQRLLDVFLSVGVMAYDSAWYEKDVDFQKAVLMIMIRARRPVLIYAGPFTNLSHLLILGVLQTSYSYINLLNAK

>TcasOR78

MGHAIMTEILTYLTLMGFWPRSPKSSKASAFLIILSTSFLFFGILFYLIVNRQFGSSEIDSIETITSQFGVLYYLILFTWKRNDIVEIVELLSDFSKFGKPPFFDQRSTRLNYRLSCIVLILIVANIVVAALPVIYIDSCHKANEQLNLTKTCGLIAPVWLPFDYNEYPRKHLVFAWEVYCCVMNYVGSGIGALTMVGTMEHVIIRIEQLKYIFPKILDQPNPRIREQMLKNWVRYHLALFEIGRLMNDAYKWSLSVIVLCVGALFACIGISMLQSTASQINSICLFFGWFPSIAFLCMWGQRLLDSSLSVGTAVYSSRWYDMDVAFQKSVLMILIRSQKPIRISVGPFTHLSMLLLLGVFQSAYSYINLLNATS

>TcasOR79

MGHVIMNEILTYVTLLGLWPRSRKSTKTISYLIILSSSFLFFGSLLYLVVHRKFGSNEIDSIETVTSQFAVLYYMTFFTLKREGTVRIIDQMSDFSKFGKPPLFDQHNKRLNYLLSYFVICLFVAIVGVVALPAIYTGSCHKANEQLNLTKTCGLVAPVWLPFDYNGYPLKFLVFAWEGYCCIITYACSGISSLVLVGTMEHLIIRIEQLKLMFPEILNEANRHIREQKLKNWVQYHLALFGIGKLMTATYTYCLSVIVLCVGILFGCIGVSTMQSASSNNSVFLFLGWFQSLIVLSVCGQRLIDTCLSVGIAVYNSRWYDMDVSFQKSVHMILIRSQKPILIYTGPFSYLSHLLILSVLQTAYSYINLLSARG

>TcasOR80

MGHVIMNEILTYLTFLGLWPRSRKSTKTVAYLIISSTSFLFFGSLFYLIAHRKFGSNEIDSIETVTSQFGILYYWVLFTLKREGTVEIVERLSDFSKFGKPRFFDQRNRRLNYLLSYFVLVLMVAIGGVVALPVVYIDSCHKANERLNLTKTCGLIAPVWLPFDYNEYPRKNFVFAWEVYCCIMTYACCGIAALVLVGTMEHLIIRFEQLKLMFPEILDEPDRHTRQQKLKNWIEYHLTLFDIGKLMTSNYTYCLSVIVLCVGILFGCIGVSTMQSASSHNSVFLFFGWFQSIGVLCIWGQRLLDTCLSVGIAVYSSRWYDMDVSFQKSVLMILIRSQKPILIYAGPFSYLSHLLILSVFQTAYSYINLLGAKG

>TcasOR84

MTEEKELRLCLWSCYYLKLSLMWPLKREEFKSSKGLYLRLLVFVIISGSTFTAMIFMHLYKSLKVGSYDVSEDLAILASNIGYVLMMTMYVSRQKDLELLLLDLSDFKTYGKPPNFDKVRKRMDLYAHLIFFYSMFGSFVYNMDKIILIDKCKEARRINEVCGSAIPFWTPFETEDLFTLTLVITYVLINIFVVVKVAMTVSVQVLEISSHINLRIEQLKIFIAGCFDRDFKASRERLDFCIRYHNVIIDFSERFSRCFSYVMFIHLAITGIIIGCLENQIVQEHQPEAMLHMGGWSTATFIACYGGQLLMDASTSIADEFYNCPWYEADVKMRKDLILIILRAQKALFVSTGPFNVLSFALFVSIMKLSYSIFTVLS

>TcasOR86

MALNQEDAICSKSCFYLRYSFLWPEEAPTRSFYAKFILVLILSFLTAFLPLFIHFLILVERGLDPSEDLFVIISYTGFALIMIIYVIHVKKTSYLIVQLSDFEKFGKPRGFDYWDKKFRLISSGVYYYVLIASSGLNLGRWVGMAECRKERDFQVCGIVIPYWLPWKVDSWLFFILLDLYVLKMTLVVNCALFLIIIQILEITTHLKLRIDHLKEMLVKCFDSDSQTNRKQLVNCIRYHTYIINCSKLFKKCFTHAMFSLIVTMALSCGCLESQVVKFDLWALPPISAWIFILFIACMAGQILMNASLSIGDAGYHSKWYQTDANFRKYLILVLMRSHKALVLSAGPFNILCFELFVAIMKFSYSVFMLLNQN

>TcasOR87

MKHVIMDELLIFLTFLGLWPRTPTSPKIISYLMIYSTSFLFFGSSIYLILHRKFGSDEIDTIEIITSQFGVLYYLTLLVVKRDGITKIVNLLSDFSKFGKPPLFDQRSRRLNLLLRLFVTVLLAATVAIVSVPVVFINSCNKQNLQLNATKICGLAAPVWLPFDYTQNPRKYFVSAMEIYCATMNYAGSGSGAFLVIGTMEHLVIRIEHLKNMFPEILNEPDKQIREKRLKKWIEYHLSIFEIGELMNETYKWPLSVIVLCVGILFGCIGVSTMQSVSFQNSSVFLFFGWFQSIFVLCFWGQRLLDSCLSIRKAVYNSKWHEMDVSFQKSVLMILIRSERPVLIHAGPFSYLSNLLVLGVLQTAYSYINLLNARS

>TcasOR88

MTEEKQLRICLSSCFFLKWSFMWPTKSEEFRTSKGLYFRLLAFVIISGLTFTAMIVMHLLKSVEAGDYDISEDIAILATNTGYILMMLLYIIRQKDLESLLVDLSSFKKYQKPPKFDEVNRKLEWCTRMVFGYCVFGSVFYNLVKILAIPSCKKSRRINEVCGVAIPYWVWFDTENWSIKLPLILHTFLVIIIVDKVTLLVSLQVLEIACNIKLRLDQLNCMLVSCFDGDVEASRRRLNECIKYHKEIISYSEIFSKCFSIEMFTHLTTTGIICGCLENQVVQEHRPEAILHIGGWITAIFVSSFGGQILIDSSLSVAEAAYSSAWYEADVSLRKDLILVILRAQKALFVSTGPFNVLSFALFVSIMKMSYSILTILQ

>TcasOR89

MKEAVLQQSKKEMHLLNLWPKGHVKHFRFRYVITLIIVSPFTLGTLTHFINVLKENLDVDLSGDISVIAVVTGLHFMLITFVWGHKKIAYLWENLGPHEYFGKPDNFEKRCKQLNFYSRLYAYYCYLGLTVYIIMKNRGGIECRRLNVERNLTEICGLVTTFWAPFDIDFFPFRQILFVDQVFATYFIVKGGAAISFTTLEVGEYIILKIKHLKRLVKEVFDDPREEVQRKKLVFCIKYHQYIISIQELYDGRYKHCNGCYILMVGIIIASLSNEIMKNHNIEALLHLVGWVFSFYICCFSGQSLLSESLTIPDAAFESKWYEAPVYMQKDLLLMMLRSQKPLMLHATPIGVMSLSLFITLVKTSYSYFTLLNQST

>TcasOR90

MAKDTSPVLRESIEVMKYLQLWPQNERTNLRRRYFIVIFLCSPLHLGLATHLVVCLKDNLDVDLSANIAVLSAVTGLTYMLIVFVWSQDKLVHLLAKLDTHEIFGTPDNLTKRSRRLNFYAKLYSYYCYFGIVIYSLVQIIEMPQCRKMNEEKGLSEICGMIVPFWAPFDIDWFPLKQIFWLNQLLGIYIIIKGGAAVSITTFEVAQYICLKIKHLNRLLREAFDDPCDVVVEQKLLHCIRYQQHIIRTNELFNVCFKHCNGCYVVMVGIIIASLLNQILKEKSVGALVHFAGWICSFFICCHAGQAVISESLTIPEAALDSHWYEAPVKYKKVLLLLLVRSQKAFNLQATPIGIMSFDLFIALLKTSYSYFTLLHKST

>TcasOR92

MKNQEIKICRATLTVLKYSLIWPSEADEMNPGKWYYIRVVTFILFTCPWVLSVFMHLIVSIRNNADIHLSEDVALMVAFTGVYYMTIIYVKKQPKVAFLLRDLSYFQFGKPPGFDETERILGFLSKLTFCYSVMAVVIYNYIKYRQKPECERMNKLKGLKENCGMLTPTWWPFEINYSPAFQLIFLYIFTSTQVMMKLSLMISFNVLEMAHHIILRINHLKTMILESLDEQDYEASKRKIKTCILYHLEILGFAERMDDCFSNGMFAHLTITAAICGCLEKQFVDGDNQLGSLLHIFGWILALFLACLGGQHLINASETISDAIWSSKWYDADLRLRKDLIFMMARSQVGLYLNVGGFGILSYALFLSVIKMSYSILAMLTS

>TcasOR93

MTNLEIKICRATLKILKYSLIWPNEADEMNPGKWYYIRVATFLLITSLWVLSVFMHIVMSIIHDADVHLSEEVAFCVAFCGLYYMTMIYVKNQPKVALLLRDLSKFQFGKPPGFEEKERILGFLSQFFFYYCVMAVMVYNLVKLLQKPDCEKMNEIKGLKENCGLLTPTWLPFDINYFPAFHLTFLYVFISTQILMKLALIISFNALEMAYHVILRIDHLKIMITECLDQRNYEVSRRKLKTCILYHLEILSLSNRLNDCFSNIMFAHLTITAAICGCLEKQFVDGDNRLGALLHVCGWISALFVACIGGQHLLNASLSIPDAIWSSKWYEADVRIRKDLLFMMAKSQVGLHLNVGSFGVLSFSVFFSVLKMSYSILAMLTS

>TcasOR94

MAIKICKFTRKNMQISLIWPREFEEINPGKWYYIRIVIFLITYGVFPFCTFLHAVVVIHNNLDIRISEDIGAVVSNIGISYMAIIYVQQQNQIAYLLKDLSDFKDFGKPPFFEEENKRLNFWSICTFIYPTCGASLYNLSKILEKSECNKINEENGLPATCGFIFPIWVPFNINYFPLFHIMLISTWFCTTMFVRLHLSISYNAFEIAHHIILRIKHLNGMIITCFDCQDYKISRQKFTTCVLYYKQILDLSNRLNQSFSSIMFVHFTMTSAVCGCLEKQFVDGEYVGGFIHLVGWIISLFIASVGGQDLVNASQSISEAIWSSKWYLADIRLKKDVLFMLMRSQKDLHMSVGSFGVLSYAFFVSVLKMSYSILAMLTS

>TcasOR95FIX

MVVKESEIKVSRVTRKILQYSLIWPKEGDEINPGKWYYIRIFTFLSFTSLWCIAICMHFIIVLKDKIDWDVTEEIAIIIAIYGTYYMVLAYVKNQKKAARILRDLSNFERFGVPPGFEEEEKRLKVYIIGIFIYAFLTITFYNFFKLSQKGACERFNEEHHLDENCGLLSPVWIPFKVDRFPQFELVFLYLFTCCHLLMKLPLVVSYNALEMVHHIILRINHLKIMITECFDEPEYEISRRKLTQCILYHIEILEFATRVDDCFSNCMFAHLTLTGAICACLEKQIVAGISRFGAILHFIGWILALFIGCLGGQHFINASDTIPESIWASKWYNANLRLRKDLLLMMMRSQRDLHITAGPFGVVSYALFLSVLKMSYSILCVLTS

>TcasOR97

MNNQKIQISNMTRKVLRYSLLWPKTNEELNPGIEYQFSVLGFFLVTGVLVLCITIRFFITIKAVHEVDAEVLAILIASYGSYYMICAHLKNQHKVALLMRDLSVFNNFGKPPNFDKRNNQLNFVAKLLALYSFLATIFYNGEQLINKTECKRINKEKGLSDHYCGLLAPCWLPFEIDYFPVFHLILIYAFTSGYLLIKMAIHISYNAFEIVSNIVLRIEHLKAMILETFENRNKQVCHKKFLQCILYHIEILDFAARLDDSFFNSMFGHLALTGGICACLEKQIVSGVNVVAGTLHFIGWILALFIGCVAGQYLINASEILPSAIWTAKWYDADLELKKKVLFMLARSQKSLFIRAGPFGILCYPLFVTVLKTSYSILCMLTS

>TcasOR98FIX

MVKKESEIKISRVTRKLLQYSLLWPTEGEELNPGKWFYFRIFAFLSFTSLWCIAICMHFIFVMKDKPDWDPTEEIAIIIAIYGTYYIVLAYVKNQRKAAGILRDLSNFDKFGVPPGFEEEEQRLRVYIICVFIYGFITITFYNFYKMSQKKSCERFNIEHNLHENCGLLSPVWIPFRIDKFPRYELVFLYLLTCCHLLMKLPLIVSYNALEMVHHIILRINHLKIMITECFDDPDYEISRRKLTQCILYHTEILEFATRVDDCFSNCMFAHLTLTGTICACLEKQIVAGFSRFGAILHFFGWILALFIACLGGQQFINASDTIPEALWASKWYNADLRLRGDLLLMMMRSQRDLHITAGPFGVVSYALFVSVLKASYSILCVLTS

>TcasOR100

MSPKDKIKICGITRKVLRYSLLWPVENDELSPGIRYKLTILAFFSITGILVFSISVYSVLEIKQGYDIDVEDVAILIAVYGTYYMVSAYLNNQHQIALLERDLSQFYKFGKPPGFEQLNSQLNFAVKVLIIYSFLGTFVYNGTKMLLREECKKNSQEKGLSDNHCGLIATFMFPFRVDYFPVFYIVLVITFLLAHTLIKLCMHISFNAYEIVNHIVLRIEHLKEMILSCFNERNQTIVQKKLRVCILYHIEILDMAARLDKNFFNTMFGHFALTGAICACLEKQIVLGVNIVAGTLHFIGWIIALFVGCVAGQCLLNASEIIPNALWAAKWYHADLRTQKTLLFMLARSQKELTIKAGPFGILCFPLFVSVLKTSYSILCMLTS

>TcasOR102

MQNQSKPCQLDMMDETYLQFFVKSFTYLNMLPEKTTFCTTIQQYYVSVIITITTFPILADLVSQFYEESISFTSVNENFVALSALFAVIYVSVCFINRKHKIRALIADLALFETFSSKAVITETDKSVKFYTKLFIVYGIVGNLCYGLLPILGYKKCHESKSVHMTRYGIPCGLVVRFLFPFKFDYSPLAELVALYEILVCILGTSVVIVVTTLICGVLIHITVQLQCLRKIILDLSQVNDLEILEHKMKFCVKYHTAILDYGIRTDLAFNQMMLLHITWTGFIISVLGFEISTTDDYVEAFRFFMHLLGWLGMLFVVCYYGQKILDESLAIADAVYTFLWYKKSVIVQRYVLLILLRSQKPLTLRACGVKVMSLATFLGVLYSAYSYFTLLLKLKP

>TcasOR103

MKQALKLADVLGFNPLKNDNLTKLKKYSSLICMISVVVSAILEFVSNFSALETYESAPESLVPQFQTLAKISSLLLSQKDITELIDEIKYFWKLDQFGDFHTRKLKKIYKYVTIFFYFYTLMLSGACVLFTITTVIFTPEKPLFLCYGGLHGLPSPQFEIYFVVDLAAIVIMSFGVAAYDGIFFYFAFHVYAEFKLVKVAFKGKSTFIEAVKHHDFLLKYLRKLNEIYSPIFLCQFFSNLLGICFCLFMLSRSGMPPELTSFSKYFISLVAFTVQTYIFCLIGDLVSELSLDISNVIFYVDWLDDEVYKSKTARLVIMNKAQSPVKLTIGKFTGMDLRTFLLIVRNAYSFLAFVNNALD

>TcasOR105

MKPALKLANVLGLDPLRNDNYTQLKKMFCALCIVSLFVSAYLEFFSNFTTFETYETAPESLIPHFQTMFKMYSLIFSRTEIVELIQMAEQFYKFSQCDERKKLTKLYKRVDLFFYVYASLVAAACVLFAIVTLIFKPGKPIFLCYGGLHGLESPEFEIYLVVDLIGIVIISVTVPAFDGLFFYFALYIYTEFKLLKIAFKTMSGQELREAVKHHDFLLKYIKKLNSVYSPIFLYQFFCNLLAICFCLFMLSRSGIPPEMVSFSKYFLCLLAFLVQSYTFCSIGDLITELSEDVSNAIFYTDWLDDEAYENKTARLIIMSRAQNPVMLTIGKFANMNLRTFILIVRNAYSFLAFVNHALN

>TcasOR106

MESALKLIDIIGLHPLKSDKYSTMRKTISFLSLVVILISAQLEFLSHLSVFEVYNSGPHSTIPPLQSLLKMATLHFYKNELIDLMEKSKSFWKLDKFGDLYKQELSKLHRLVTIIVYIYIALLTATCVQLAVLTLIFRRGKPIFLCYGGLYGLESPHYEIYSILDAIGIGVISIAVSGYDAMFFFFALDIYTEFKMIKSAFKRHSDQTVSSYNKQFIEAVKHHDFLLQYINQVNDIFSPMFLFQFFSGLLGICFSLFMISRSGLQDINTLSIYSAGLLGFTAQSYTFCLVGEVISELSEDISNEIFYTDWLDDEVYRNKTAILIVMNRAQESPKLTIGKFADMNLRTFIMIVRNAYSFLAFINNALD

>TcasOR107

MENPLKLLHIIGLDPRQSDKYSTIKKVISFLIVLAVLLSALIEFFLHHNESQVYDTAPQSTVPNLQALLKMFALIIYKKELIDLFTKGNHFWKLDKFGDCHKQKLTKLHKYVDLFFYVYAVIITGAFLQLALLILIFEPGKPIFLCYGGLYGLESPQFEFYAVLDFLAIGVIAISVTAYDSIFFYFALYIYTEFKMIKIAFKRENCAQFIEAVKHHDFLLQYISKVNEVFSVIFLTQFFSGLLGICFNLFMISTQGTRDMKSFSTYFVGLVGYTAQSFTFCLIGELISELSEDISNEIFYTDWLDDEVYRNTTARLIVMNRAQESPKLTIGKFADMNLRTFIIILRNAYSFLAFINEVLD

>TcasOR108

MGSILLLNSVLKKMEKALKLVNILGLDPRKNDTFSKFRSIFCFTILISASFSSHLEFFLNFKGLETCERAAESIIPQYQTMCKMATFLLYKTEMLDLIKKSERFWKLDRFGDLQAKNLHSTYPIFQIFFYVYVVILFLTCAMFALVNWIFDTGKPISLCYGESEGLETPWVEFYIVLQSVEVTIIFLGITGYDMVFLYYAGSVCIQFQMLKMAFAERKMNERQFLKAVKHHEFLLQYVEQLGDIYSMWFLLQYFSSLFGICFGLFLISKEGLPTEPERLSKYFPYIFSFTMQSFTFCMTGTMLSDWSSEISDEIFHSDWSDDQVYKNKTARLIVMNRAQRPAKISIGKFLDLNLRSFILLMRSVFSFLAFVNNILNRIN

>TcasOR109

MGKVKFTEPLEFLNVVGLNPENCSNFSLFRRVISLGFFLVVITLGLLELLLHFEGLETCSRASEAMIVQYQLFIKIAVLLKHRKNLVVLMQKTRKFWPLDKFGQDAKIERPHKLLKAFFFAYKLIMILMALQYILRKFVSKNGKPLAIAFGESKGLSPKVDHLYFVLHSTSTFVVLHAVTGFDRLFFFLIGHVLTELKLVKKSYRLTQNRREKFLETVQHHAFALEFVRKLNRIYSQVLLNQHLSCLFGICFGLFLVSKDGIPPDLGHVTKYVPYVISFITQTFTFCFIGSLLITWSLQVPDAIFYNDWGKNQAYKYKTDKIIAMIRGQRAAKLTLGGFGDLDLESFNLVVKNAFSFFTFVNAMNQK

>TcasOR110

MDKVEFSDPLFFLNVIGMHPFKADKFSKFRLAFSIAVYFAVIFSGVLELIVNSQGLETYARASDTLIPQCQLVCKIFVLAKYKKQIARLLNGSQRFWDLGQFGARYGNSFGKTHKYLKSFFLLYKVMLTFTCLQFLAVKIIFKIPKPIAISFGETKGLEPLYDHLYLVLHAMITLVTINLVNGFDGLFFYFIGHVLTELKMVKVAFGDSPIETNWSEEKRFKFAVRHHRFVLDFIEQFNIVYCTMLLVQHLTCLFGICFGVFLMTKDGVPPDLDRASKYLPYIVTFIFQTFTFCFAGNLLLSWSLEIPNEIFYHDWAKKTTYENKLAKIISMKRGQRAARLTLGGFANLDLDSFRMVLKNALSFFTFVNAMMNKKAVTSV

>TcasOR111

MEKVRLTEPLFLLHIVGMSPHDSGTFARIRKIFSILVYTSTVVLSMAELFFNYKDLETVIRATESFFTQYGLAWKIAVFVVYKTELAQIIRLCDNLWPLDEFGTGHNFQFLHKFLRRFFLLYTGNLALLCTQFAVTAFFDDQFKSVMVYYGEKESRSQIYDNFVFTLQVIYLYVGCFVVAGFDCFFFYLLGHAVTELKMLTISFSCKEIGRNWGYEERFKCSVKHHIHVLELLDKINKVYSVMLLNQHLCSLFGICFGIFLMTKDGIPPNVDHFSKWSTYIFTFILQVWTYCFAGDQIMHWSLKIPDEIFYDNYWNKYSLKNGLNKIIAIQRGQKAAGVSLGGFAMLDIESFNVVIKNAVNFFMFMDKMYKRE

>TcasOR112

MITRLMAQFAIKGRVGTGGYIMDKVKLAQPLAHLNIIGLDPLKNDRFSKIRTVITVAVFALCNVFSFSELFLHYNNPHVIVRSSEVVFPFFQNDWKIAIMLVYKKNLAQLIQNTSRFWQIDAFGKNYQYSMGIKHKYVRIFYLVYRLMLMFSCSQYILLTIGSDRPMILSFGETGGLGSGALLFYLIFHIVYLLIIFNVINGFDGLFFFLVAHVLSELQMVKVAFSSSKVITFWNHKRRFKSAIQHHRFVLDYINRLNSIYSILLLNQHISCLFGICFGLYLFISDGFPPDYEHISKYVPYVIYYITQVWVFCFAGQLIIDWSVNISDEIFYHDWTLNRTYENKTDKLIIIQRAQHAARLSLAGYGNLDLQSFNLVLKNGLSFFTFVNAVIHK

>TcasOR159

MRGKTIESTTNPYSSLKKVFIDFAYSKLVISYTKASLTFHVLSLLLEVYYLVTNFSVELICRYGCMMCLMTYMYSKKLKLLEKPCLLDFWKVYNSSTATQRLISEKSSKTNRRLYCALTCCFFLAIILFPIWGDLNEFFIFSQVYEKYFTSWAPAFCYFYVSTLLWCCFYCFHLPGIIMYLTLHLDLQFKLIKDKITEIDKNCSQKEIYQILRLCISHHVALKKWMDKLADLLVTIMPFFFLFGALNSIATSFFVLYTLQNTTMILKIRLGTLTLCNFIIVSTFAEVGQIFSGQNNSLFEQLMDCSWYLWNIKNRKTLLMFMLNCMKPKTFSWGGITLNYSFVLFILKTSLSYASVLFKLRGETF

>TcasOR160

MSGKTKRITTKTIHLSNPYSSFKKVFSDFAYSKIMIFYTIATLAFHMLSLFLQIYYVATNYSVELICRYGPMMCLAIYVVTAKVVGVFYYKTFTMLENQCLFVLWKTCNSSPTTQRLILNKSLKMNQKLHLALMSYFLLAIVMLPTWGDLNELFIFSQVYERYFKFWAPVLYYFYISTFLWCSYYSFHLPGCILYLTLLLDVQIKLINDKITEIDQNFSQNEISETLRLCISHHIALKRWMSTLAKMVNSVMPVFVLLGALSTVAVSFFVLNTLQNTTMILKIRLAILTVCNFVIVSTFAELGQIFSDQNNSLFEHLIDCPWYLWNVKNRKILLMFMANCMKPKTFSWGGITLDYSFAISILKTSFSYALILFKLRGETIRN

>TcasOR164

MSGKTKRTTTTRKINLANPYSSLKKVFIDFAYSKIMMFYTKATLAFHVLSLLLELYYVATNFSVDLICRYGCMICLMTYVVTAKVVGIMFSKPFKLLEKQCLFVFWKTYNSGPTTQRLILDDSLKMNRKLYLALMFYLLLAIVLLPVWGDLNEIFIFNQVYETYFKFWAPVLYYFYISTFLWCCYYSFHLPGSIFYLTLHLDLQIRLINDKITEIDQNFCQNEISETLRMCISHHIALKSWMSKLAKLVDAVMPVFVLLGALSTVAVSFFVLNTLENTSLILKIRLTTLTVCNVFIVSTFAELGQIFSNQNNTVFEHLMNCPWYLWNITNRKTLLMFMLNCMKPKTFSWGGITLDYRFALTILKTSFSYALVLYQLRGETN

>TcasOR165

MSDNTKKATTKSLDLTNPYSSLKKVFINFAYSKIMIVYTSATLIFHILSLMLEIYYLATNFSVELICRYGCMMCLITYMVTAKFFGMLFSNQFKFLEEQCLLDFWKAFNSGPTTQRLILKESSKMNRKIHLALTFYVILAIIMLPIWEDVNDFFMFSQVYENYFANWAPVLYYFYISTFVWCSYYSFHFAGVIMYLTLLLDLQFRLINDKITEIDQNSTQNEICGTLRLCISHHIALKRWMNKLANSVDTAMPVFILLGALSTIAVSFFVLNTLQSTSVILKIRLATITVCNLIVVATFAELGQIFSDQNNSLLEHLMDSPWYLWDVENRKTLLMFMANCMKPKTFSWGGITLDYSFALSIFKTSFSYALVLYQLRGNTF

>TcasOR167

MAKTGDIFPVRDPVKRCLFIPKLLLESTNFWPEKRNFLTKFANWVMLIICVLIESGQIAFVVVNIKDITKIASAMSTVSTTFQAITKLTVLYIYNDKLRLILKSVWYEFWPSYTAGREINTKLETYNKIVIVSFLTILISGICFAFGFLSSPLISGERILPFETVYPFDWTKSPYYEIIYVTEWMTNIAFILIGICGHDFLFMGLCSNVVGQFTLLRELFGYLGTKNVAQIIKKLGHDTNIEPNRQLLRICIIHHVRVTEICKEIAEIFSFSCFIQLLSSVTALCVGALIMTFADIDAALFTVSSAYIVGHLLQLFLYATLGNEVIYYASRLPNAIFHSHWYNIDLEVKKDILFVLQRAQKEVKISAMGVSVLDYQTFIQVLRLSFSFYTMLSKVTDH

>TcasOR171

MVKLFLLLKHLTMKAQDSDNPYIVLRRVFVDFAFTSHMIIYTKITFVFHFLTLLLETYYMITNFNVELFSRYGCMMCLMTYSNVQIVLAKLLEILFARHIKFLEEERLSHFWKLEESSEETQKVVNAESSKIRKKTFFVLSWFVALGFVLFPIFGDLNDLFMFGRVYRNYFGSWAIIPFCIYVSTFPSIAYNSICLPAVVSYFIFHLNLQISLINDKLGKISEKSRQSEIYQKLCSCVAHHVRLRRWTNIFQNELESALPFYLFLGAINSIAVSFFILYNLQNMTLIFEIRLVVISVCNVLILWIFAEAGQEFSDNSDSIFDAVVACPWYSWNAQNRKIMLIFMLNCLKPMTFSWGGVKLDYQFTVTIVKMSYSYALVLYNWRYEK

>TcasOR172

MSFQALKHLLKMCAEKTPDLDNPYLTLRRVFIDFPYSKSMKIHTCITLLFHFLSLILEIHYLVTNFSFELSSRYGCMMCLMTYVISVKIFVIMFAKPLKILEEQRELHFWKIGDSSHAMQQSVATEALQVKKQTYFALSCFVLLAVILYPVWGHVNDLFMFSQVYEKYFGDWSVIPYYFYVFTFMSSSFNSFQLPGVILYFTLHLNLQISLINEKITKISGENYCQDEVFKQLRDCISYHVALERWMARLIDLTKTAMPVFILLGALSSIAVSFFVLYSLENTRFILKIRLTVVAICNVLIVATFAKAGQRFSDKTGLIFDAIATCPWYSWNVPNRKIVLIFMANCLKPKTFSWAGITLNYQFAIKIVRTSCSYALVLYKLRNGNY

>TcasOR187

MSTKREVVKNFPYYYLFKICIDFGYSNVVKRLNICCITMIVMFHLTQIHYMQENFSKELILKYGSGIALGIYTILSMSVQMLIEHEIKDLIAEALFSMWAVDSCGPQVEKLILRRAKVMNIIYCSIFAWFALMATVMLPMWGDHSEWLLYDPILVEDVKTRLKIIYYLSTFIIFPMIAFSAIRLPGILLYGILQIHMQIMLINHKLVQVSEDLDDLNNVKKIDQDDYQERIYKELCLCVEHHIKIKLWLNKLMKIVQLLMPPYFLLGSINAIYLLFFVVYNDTSNILKVRLCILLIVGGQILCMFAEAGQALGEETGRIFDTLVNCPWYLWNKKNKQALTIFLSNSFQPYTIAFAGFTLNYSLALALLRSSVSYALVLYNMRN

>TcasOR188

MFVKRQVLEGFPYYYLLQLCLDVGYSKMMKIANIFCIIINLLNVLAQIGYIKQNFGKELLLRYACGIQLTIYTIVTMLFEFLVEQNVKKLMDEALSEMWPIDFCGLEIKKLILKRSTVMNSIFYFMFAWFAILAIVMLPMWGDQSEWLLYDRICKEFFATWWKIPYYFYFTTFPVVAFSGIRLPGLLLYTILQTHMQIILINQKLVQISGGLDGINDVRMIDQKNYQKRIYKGLRLCVAHHVAIKRWLQKPVKIVQSLMPIYIIMGSTIFISLLFATVYSFRDSSNILKVRMSVVLMICCLILCMGAEAGQALSNETSRVFDTLVNCPWHLWDQKNKKALTIFLPNTLQPVTITLAGITLNYSFAVGLLKSSASYALVLYNMRN

>TcasOR189

MEKMFPQIRTEDMKKFPYYYLLKICIVFGYSKIVKLLNVVCIIITSSTIVLQVYYLKQNFSKELILKYGCGISLTIYTIASMLVEFLIEQKTKKLLNEAGTILWPVNFCGVKVEKLILKRVTVMNIIYYFMSAWFALMGIIMLPIWGDHSEWLLCDVISNEYFETRWKILYFACSCFSFPVIAFSSIRLPVILLCTILQTHMQIILINQKLNQISEQMGNLNNIKLVDDKCYQKRIFEDLRLCVSHHGKIKKWLNKVLKLVQSIMPLYIILGCLNFISLLFFASDGLQNASNILKARLCVVLIVCCLVLSMFAEAGQALSDETSGVFDTLLTCPWYLWDKNNKKVLSIFLSNSFQPDSISVAGITLNYDFAVALLKTSSSYALVLYNMKN

>TcasOR190

MSTKKQDLLKHFPYYYLWKVFINFGYSKLTKLVTISCIIIHSSSLFVEIYYIYCNYNKEIIFKYGCMMSLLGYITISMVVELLLEKDTNNLVCEARSLFWTIDSCGVQAQQIIHKRAVVMNATFGFILMWVATLGVIMFPIWGDQSEWVLCVKIFENYFENWSQMANFVFFSTFPMVAYSTIRLPAMLLYGILQTHMQIFLINQKITEISRSKDQEKIYKELCLCVSHHVEIKRWLQRFLKMVQLTMLMLIPLGVLSCVCVLFFVIYSFLDTSNILKMRLTVVVACTVLIVYIFAEAGQDFSDEISCIFDTLVTCPWYFWDQKNKKALVLFLANSLKPYTLSIAKITLNYDFAVALVRTSVSYALVLYNMKN

>TcasOR191

MRLEIEALKNFPYYYLLKICIDFGYSKIVKCINVVCIIINSSTLFIQVYYVQQHFNKELIFKYGCGMALTIYTIASISVEFLIEKNAKNLVNDATAFVWPVDFCGEKVKKLILKRATVMNKICYFMSAWFALMGIIMLPVWGDHSEWLLCDLLSKEYFETRWKILYFACSCFSFPVVAFSSIRIPGILLCTILQTHMQIILINQKLNQISEQMGNLNNIKLVDDKCYQKRIFEDLRLCVSHHGKIKKWLNKFLKLVQSIMPLYIILGCLNFISLLFFASDGLQNASNILKARLCVVLIVCCLVLSMFAEAGQALSDETSGVFDTLLTCPWYLWDKNNKKVLSIFLSNSFQPDSISVAGITLNYDFAVALLKTSSSYALVLYNMKN

>TcasOR192

MVSEQTLLKNFPYYYLLRIFIDFGYLKITKVLSVACIIIHSLSTLLEIFYICQNFSKELVFQYGCITSLATYVITSMTTGFIIENDAKNLIRETVTAFWPIDFCGPQVEQLIFKRVARINTFNFFLLAWFAIFGIIMFPVWGDESEWMLCVIAFKKYFPKWWRVPYYVFFATYPMVAYSAIRIPAMLLYGILQINMQFFLISQKIIQISQKPQNKTHQPGFYQKTVYKKLCQCISQHAEIKRWLQRFLKMVKSVMPVFIFVGGLCFMSILFFVVYTFQSTSNILKVRLGVILMICNLILVTFAQAGQTVIDESSGIFDTLMTCPWYLWDEKNKKTLVIFFSNSLKPITFSIASITLNYAFAVALLKTSASYAIFLYNIKN

>TcasOR193

MSELEKQLPYYFLMQFCINFFYSKTVKVVTSSCIIIQSLSLLLQVYFIITNFSKELILKYGCEMSLATYLLTSLLVDVVVENTTKQLISEGHTSFWSIDSCGHDVKNHIIANSARLSVVIYFILAWFAVLGISVLPVWGDQSEWILFVQIFNTWKKILCYVYLSTLAVMVFLSIRLPAMLLYGILQIHVQIILINQRIIQIGRENTNDIRMMNQMSYQNRIYKELGFCVSQHARIKRWLKKLLGIVQSAMPIFTVLGGLIFISVLLFVLYSFENASCFLKIRLGMVVISCSLVLCMFAVAGQAFSDETSRVFDTLMTCPWYLWDQKNKTILLIFLSNSLQPINFSIANITLNYSFAVALLKTSTSYALILYNMKN

>TcasOR194FIX

MAMKQYPFLYKIFLDFAYAKIGKMVTYSCIIIQSLALQLQVYFIVTHFSKELIVKYGPGVLVVTYLVTSLVVELMIENKTRKIIDFARLTFWPTDFCGLEAKNRLIKNSSKVSIVIYLILMWFAAQGIVMFPVWGDTSEWRLHVEIFDQWKLFYYIYVSTFTIIVFSAVRLPGILLYSIFQTHMQIVLINQKITQISQNDPNDIRMMNQTGYQKRIYKEMCLCVSQHIAIKRFIKKLLEIVRPVQPIFMVLGLLGVISIFFFALYNLENTSNILKIRLVMVVISCILILCLFAEAGQAVSDETSRVFDTLLTCPWYLWDQRNKKALAIFLSNSLQPISFSMAGFTLNYGFGISMLRNSASYALILYKMKN

>TcasOR195

MFRERVYDDRFIVLKTIFLEFAYCKEMKIYNMFCLVFHLFSFSLQVHFIVLNFSVELITRYGCMLTVFLYLIAAKSFSIIIEKQVRMLEMEATSFFWPIDCCGPQVKKNIYDRAARQNIQNYFTLAWFALFGIIMLPVWGDQSEWFLCIQVFQQYFGCWKLFYYFYFSTFPMIAFTAFRLPALMLYGILHEHLQLILVNQKIVQLSVRRSLKENIVDNANYQKTVLKKLKLCISHHVKLRDSLGKLIGVIQLAMPVFLFIGALGSIAVLYFVLYIFLSSSNILKIRLVVITICNGLIVYTFSAAGQALADETGRVFDTLMTCPWNTWNIKNRKVLLIVMSNTIQPLTFTLAGITLDYKFGLTMLRISCSYALILYNLH

>TcasOR197

MFKKRKFDDRFIVFKKIFFEFAYSKEMKIYNMICLVFHSFSFVLQVYFIVQNFSVELITRYGCILAVFLYLIAAMSFAIFIEKQVKMLEVETTSFFWPIDCCGPQVKKLIYDRSARINILNYFTLAWFTLFGIIMLPVWGDQSEWFLCIQVFQQYFGSCWKLFYYFYFSTCPMIAFTAFRLPGLMLYGILHIDLQLVLIYQKIAQLSARRIFSENIVDNAHYQKTVFRKLKLCISHHVKLKTCLRKLIELIQMAMPVFIFVGAVCSIAVLFFLLYVFSSSSHILKIRLAISVVSNVLIVYTFSAAGQAIADETSHVFDTLMTCPWNAWNNKNRKVLLIIMSNTLRPLTFTLAGITLNYKFGLTMIRISYTYALILYNLN

>TcasOR198

MPNVTNKRQKRLFSKTRTKSEDPFVMIKDVFVDGGYHPVTKMLNYICLVIHSCSLLLELNYFVHNYHFDLMMKYCCAMSLMGYIIATMLFAIFQEHSAIDLTKDILSLFWPIDYCGPRVKEEIVKKATKINRIHYIVLLFAGALGITMFPIWGDQKEWFLCVQVYQHYFGKWSKIPYYVYFFTYPMLAFSSVRLPFMTMYAIVQIRMQVYLLHQHISEISGEYVYDMKNLQILCDQNYQNEIYDKMRLIISHHIMLKRWMRKLVHTVQISMPVFVLLGTMTSISVLFYAIYSFHNINFILKVRLISVSVCTVLVVYMFSEAGQALSTETTGVFDLLMTCPWYVWNIKNRRILLIFMANSLEPMTFSLAGVTLDYRFALGMLRTSCSYSLILYKLKTGI

>TcasOR204FIX

MTNFFSNFCSPLKNHWAKTKHLFSKFSLSSDQPFIMIKLVCVDIGYHPVAKTINYICLAIHISSFLLEMNYLRLNFSTDLLIKYGCGISAVVYDISTLIVAPMIERPTIGLSEGITTSFWPIDFCGPKVKQLILEDTKKTSKIYYRTLVTIFGFAAVIMLPIWGDQKEWFLCVQVYEHYFGKWAQIPYHIYFLSFMWFAFTSVRLPLMMSYAIKNIRVQVFLVNQKIAKMSKEYEEAKIEDVNYQNRVYKNLRLCISHHVLLKWWLRKLQKIVRFCLPVFVVIGILTESSVVFYLIYNFKKVNLLLKIRFLLLACTTGVIIYFFSEAGQSLYIETSQVFDSLISCPWYSWNVKNRKVLLIFLTNSLQPMFFSLVGFTIDYRFALTMIRTSFSYAIILYNLSSGSQIASI

>TcasOR205

MTNIFSNFSLYFKNTWTKTKQRFSKTLPSSNVPFMMIKLVFVDIGYHPVSKIINYICLAIYMSSFLLEMNFLRLRFSTHLLIKYGCGSSLSVYFISSMTVAAMTELLAVDLSEGILSSFWPIDFCGPQVKQLILKQSRADKRMHYVVLLVFSITGLAMLPIWGDQKEWFLCVQVYEYNFGEWSKIPYYIYFFTFPWVAFSSLRLPFMMNYAILNLRMQVFLINQKIAKMSNAYDQTTIEDVNSQKRIFKNLRLCISHHILIKWWLRKFVNHVKFCIPIFVIVGIATSISIVFYLIYSFQQVNLVLKIRFLSIACCCWFVIYLFSEAGQSLYEYTEIFHSLISCRWYIWNVKNRRILLVFLANSLEPMTFSLAGITLNYRFALNMMKTSCSYALILYKLNCDSQIMD

>TcasOR213

MAKFNDPFKFVRTIIFVDMNSYKVIKTCNVLLNIIYSLIHCLLIYYLCKNLEINLLIRYAPAILLFILVIFGAVFSIYMDEDILEVRSVFRENRWSLSVLKENSQTKLGRKCQFINIFILLVLLLIVSTLAINAPCFGNQRELLICIQVFEEYFGEWSFIPYYFFFLGFPLLYYNFFRLWMTFVYGLLEGQLQFFILEEYLCGIYETEDSKSWKYLQDSRYQQEIEKSLRLCISHHIGLKKFLKMVENQTLKVMPFYLVFGVLILICYFSFIINFADTVTTIGKIRMFMTAICMMGVAILLSWIGQQLIDVTSDIYFTLGGAPWYYWSQKNAKLLLMFLTNCTKNESVTLAGISLDFTLFVSIVHTTLSYALVLYNLRESSLVSSSQK

>TcasOR229

MSARPLHLRNFPYYFLKVLVFDFEQYSAGKVLSYFCAIVHSISIFLQMHYLVKNFTKETMFQYGCVLTVLTYCVVALFFAIASGNFVEKLESEISSFVWPLDICGEDVKAAILKRAFYTSLVAYITIIAFPIFSVIMFPVLGDQSDMFLCVRVFNEYFTKWSQIPISLYFYSFPVIAFSGIRLPGMLLYAILITHIQMFLLNRRIEQISELSNQRRVFETLCSCIELQAKLKRLIRNVFQLVYIAMPIFILLGAVSSVFVLFFVVNSLETASYFLVLRMGCFFGANVLVVFIFSQSGQSFSDETGRIFDTLVMCSWYNWDKRNKKVLLMFLANSLEPMSITIAGITLDYKFALAMLRTSCSYALVLYQMKN

>TcasOR230

MREKPLHLSHFPYYLLKIMLCDTEQYRLGRFLSYSCAVIHSISLLLQMYYLIDNFNKETVSRYGCVVIVTTYCVVALIYEILYAQPSVSMMSQQISTLWPMDACGEKVKQMILKRAFFTSVVTYSILFSFPIFGIIMFPLWGDQSDMFLCVRVFNEYFTKWSKVPIYLYFCSFPVLTFSGIRLPGMLLYAILITNIQIILLNQKIAHISDLGDQRLVFGTLCSCVSLQIKLRQMLNKVLQFVYLVMPVFLLLGALTAISVLFFLFYSLENPSDYLMIRLACFLGGNILVVFTFCESGQALSNDTGRIFDILLTCPWYKWDKKNKNILLMFLVNSLKPMSITIAGITLDYKLAVTLIRTCCSYALVLYQMKN

>TcasOR234

MQQAALRNFPWHYIKRIFIDFGYHRTMKIFTIVYFILYSGSLLLDLYYLFNNFSIAAMVRYGCMIMLISYVIAGMLFCFIFEKQLLNLLSEAETIFWPPEMITSELPKFIHRTNVLNYFIIAWFGLLGVILFPVWGDQSEWFLNVWAYKAYFGSWWYIPYNLFYYSQPMAAWTCVRLPFIMMYFSLQIKLQIFLLNQQILEIPKGHNTNSETAPDDLSYQEAVSQKMCLCISHNVKIKRWTKSFLRKVIQAMPVFVLLGILGSIFVTFSVLYSFESTSTILKIRLVVVVGCTILSVYMFVEGSQRLCDESSQMFEMLAYSPWYLYNKNNRRILLTFMTNTLEPITITWGGIILNYNFGLTMLRMSFSYALFLYNIH

>TcasOR264

MVYLKDPFITLRVMFLNFNKYKIVKCCDFSFIIFYSLVFCLQIYYLISYFSANPLIRYATTILLVLWGIVGAILSVTLEKQILEATAFLDEMCWPLNMVRKEAQTKLERSCRIINIYITCSLLLILITVVFNMLCFSSQRDFFINIQIFEEYFGELSHVFNGLYFTGFPYLCYHGARLCYVFVYAILQIQLQFSLIEEYLLQVYEIDCLKSWRYLRDTRYQQEMGKSLRLCITHHNALKKFVKMINDMSLICMPFCLVLGVLILISCLAFVINFGDTLTIFVKLRILIFVVSCLCVLSVFCWSGQQLTDVSSYIFLTLARAPWYYWRLENIKILLTFSTNCTKNDSIVLAGIRLEYMLFVSMLRISCSYALVLFNLRK

>TcasOR276

MTMQFIVKRATRGIFHDLRVLKFISSDIFDIKIMKLCLFITFLIHLTACAITIHAFMFNNFSRREFISCAPVLFGCFYGLLGLGTILFKPSMTRTLMLELKAWDITAADDAVSSRIKFEINVITVFCLVNYLLALVASFFYYMSFYGDEEIFYLIRFLEDHCPNHKRVLIKLYKISFVLLGYVMVVHACQVLYATQHVRFQLILCAHFMANVTKQAKNIKDEHLPDDNNYQNMIRERLKFCIIRHQEIRRFYFDKLEEMGNLIGGFALLGCFLGISFAMHMLTSEFLRYHFARTVSSIIAGVTTFATVIAAGQSVETEVDISTRVVKEVKWYTFNESNKRSYMLMLLNSMQTYKIKFSENYSINYELGLSIVRGVFSIVSVVVQLDY

>TcasOR277

MDQVLEKFPENDWLRGVKFISSDIFQRKLVKAVLFMVLLVHLTASVITIRAILIKDITAKEFTFYGPVFFGCFYGMLAIYIILFEKNFIANLSGELKMWSFRSAGAEITRQIRFESRVVTIYAIINFVMVVIASCLHITPLESDYETFYMIRFFEDKIPDYANVCKTSYRSTFLVMGYVMMVHVYQIIYATQHGKFQIMLYLEYVKRVTQFNEKIGEKCLFYNESFQKMVARKLKNCVIRHNEFLKYHRKNTREMSHWIVAFSLCGCLLGISVFFYILSGVIYREQYFRVAVLLTTAASTFVAFIVAGQSLESRVDNGYSVVSRIEWYNFSETNKKTYFLLLVMLMQPWKIKFSDKYSINYELGLSIVRGIYSIISVMVNIRFDS

>TcasOR278

MNQPQESFLKNDYLKVLKLISSDVFEPRLVRAILFVVFAVQLTASIITVRALLIKELTAKEFVLYGPVFFGCFYGMLAIYIIIFQSSFITNMSQELEMWSYSSGGEEINRRVKFQSRVITIYALVNFLLAIVASYLYFSPLDSDNETFYMVRFIEEKIPDYAKICKIAYRTTFLAMGYVMIVHSYQVIYASQHVRFQIIFFTEYVKKVVEFDEKISEECLFYNERFQTIVGKRLQNCVIRHIQFLKFDRIKIKEMSNLIAAFSLCGCLLGISISFYVLSGIFYREHFLRVALISVTAVSTFFALILAGQSMESKANSAHIIMNNIKWYNFNQSNKKAYLLLLMMSMKQYKIKFSENYSINYELGLTIVRGIYSIISVMANMHFDN

>TcasOR281

MDYSEKSLIQGDCLKLLKVISSDIFQPKLVKLILLIVFGVHLVVDLLTLRALLVNELDFKEFIFYGPVFFGSFYGMMALLTLVLKDDFISNLKQEFRLWPLDCAGDEIYSQIKFENKIIKIFVVFNCIVTFIGSYLYFLPLDSDNETFYAVRFIEENYPDHRNLLHGLYRSTFLIFGYAMTVHVYQVIYNSQHLRYQIIIFTEYVASIGNPDKRKENELFYDKGFQKVVYERLKFCIMRHQEFLVISNKKVGDMRVFIVGYSLCGCLLGISLTFYIFSGKFYREHFPRVSVACVGAVTTFWAVITAGQAIESEYDSLLSTLLGKIEWYYFNDSNKKNYLIMLINLMQPWKIKFSEEYAVNYELGLAIVRAIYSIVSVIASMHFEA

>TcasOR282

MHDYCFEPLTKNDYLKTVRFLCCDVFEAKIVKLGLWITFGTHLIVSVVTVRALLYDLTINEFVHYAPVFVGSFYGLLALWTILFRIEMVRDVRKQFKFWTIDCAGQEAHSRIKSEIRITTVLSVLNFIITLYASYWYVYPIEGDKEIYYALKFFEEYCPRHKMVLSVVYRATFPLLSYAMIVQAYQVIYTTQHIRFQAILFIEFVLNIGHQTKNLSEEKLFYDTDYQKIVGERFKFCIMRHHEFIAFRRLKLNEMSNLIVGFSILGCLLLFSFGLFVLTGKLHREHFWRFGLGSLAAVCTFGSVIWAGQSIEIESENVVNSLNSVKWYTFDENNKRNYIIMLVNTMQPYKLKFSENFSINYSLGVSIVRAFFSILSVAAKLYFNHV

>TcasOR300

MIGLTNGDYSPRPSMEGDCLKILKFFAVDIFNPKIVRFFLWIMLLYHVVFTLVTAYFMLYVLSNSEIIGYTPAFLGNFYPMLCVWSVLFISRLIYVKEDMPLWAIDTAGAKVQASIKRKIFLYTAFGIFNLVLSLSAGSFYLKNVSEDVNVFLALRIFRDYFPNYYQVLDLIYRLIYFCFSYLMVAPSYLLIYYILHVRIQAIIFAAYVAHIDGHSDYGTDIDLFDNEEFQSEVERRFKFCIKRQIEFLLMESKKLSQISNLIAAFSLAGCLFGISIIFHLFTGQLIQEYYFRIGLTSLAAIATFSAFIYTGQSTEVQIELVDNAIDNLCWYNFNRSNKLLYLIAKADLARVRKIKFSGQWAVNYDLGFAIVKGIYSIISVVVSMW

>TcasOR309

MPFEWTIRKNKIKPILQNDVLLNLMLVPNTIISNKFLVILNYFYFGFIILQSVFVAVIIITKDEWKLLNGQYAGYTSGCAIVWSSYITMYTYVDKFLNLYKEIFPHLWSLDVVGQDHFNKFSKMAKVLKLGKNILLVVGFLSATVGLPWYRDEYEIIITVRVYKDYVDKWTTLLYFVLFSSLYHIALTVIFCVLCLVYMVLHLHNQCVMLNKRLEALDDEQLFLDNDNYQDFVTKELKFCIQQHQFLLKFAKRLNDILYYPTFYYVLSGVVTGVSLLLFPKNDIKNLLRCVLIIVLGGGFAISFCFLGQILENASEELLFSAYSARWYLWNIKNRKLLSVFLLKTQDNIVLSSSGIITINFRLLISLYQSIYSCLTFLLNIK

>TcasOR311

MHHKNIQPMTDDYLKFIKFVSSDIFQLLPVKIFLAVVFLTHAVLDLLTIYFVLFVIEPHDFITYISVFLGEFYAPLFAIVMLLFRGKITDSLKHKLAMWTITSTDEKTQSDIKRQIVFFNGFVVLNSVIISIASWFYAARLSDDVNAFFALRLIHEYFPKSIFEVIYRVTNFVLGQMMCVHVHQTLYYTQHINIQVQMFKKIIRDLENESKIEQQLKFCIERHAEFIKIITLTTKELRGAFVGFAFGGLLLGVAVAFYIFSGLLTPEYYLRVGAIGLASVVNFAVTIWFGQSTESHLDELMLAVGEVQWYNFSQRNKKVYLILLMNVMKGRKWRVSEEYSVNYRLGLAIVRGVYSIISVTSSYKKS

>TcasOR313

MEQLPKNDPLLVLRVLPELLLLHKIVRHFVVFIVCYLTATTIFCLYVLATVRGLWDLFWSQYSLLTFGSVIGFSCYFVAFWKGFKFLELRRRVFADYWALTSLGEESFQKIKKLSKSANIFTVGTILASIATSSTCMPWVGDEYDIMFPVRVYTDYFGERAVPLLVPFYLAMYCTGFVMIATGFIFVHFALHLKFQFFLLNRRLDGLQTEPLVNDFSYQNRVKEELTCCIEYHQKLLKVAKEMNEIVYYPIFIVVSSGIICSVCLIFYMKTFENSIVRGTAMAISGGLITFGFGFTGQLMENESGRLFDTSVMLPWHLWCLSNRKLYHIFLTKSQYHVSFSSSGIINLNHTLFISLYRKVTSIFSFLMNVSNKNST

>TcasOR314

MEQLPKNDPLLVLRALPEILMQHKIIKYVVLFIICYMTVTMILCSYVLATVRGLWDLFWSQYSLLAFGSSIGFSCYFVAFWKGSEFIKLRRRVFANYWPLTSLGEESFQKIKKLSIFANVFMVATILASLATSTAGLPWVGDEYDIMFPVRVYTDYFGERAVPLLVPFYLAMYCTGFVMISTGFIFVHFALHLKFQFFLLNKRLDGLRTEPLVNDFLYQNHVKEELTCCIEYHQKLLKVAKEMNDIVYYPIFIVVSCGIMFSVCLVFYMKNFKNSFVRGTTMAMTGTLTTFGFGFTGQLMENESGRLFDTSVMLPWHLWCLSNRKLYHIFLTKCQYHVSFSSSGIINLNHTLFISLYTKITSILSFLLNVSKKNHTK

>TcasOR315

MTLVRKLQAAATNAFEIRIKDDILAELFNWPFLVLDSKWSTKFAVFLTVYCVFETLACALVYSTLDVNMMGTYAIVIARFATTFCSFFSFFTKRKQYFEIINENFPHFWPLQSLGKSTFNRIKMRASSVKFYSFLNVVVMLIGAVILISFTQDESEVYLSVKIYKDYVNKWTTGFVMFFYVSFIYIGLVVAAISFVLTYTAFHLIFQCFLLNQKLKQINDSIVENEQKQAKFDEKYQSFIYKELISCVKLHQRLILFGKRINHLVYAPLLVYIFGGIVVGVALIYYLKSSVQHIFTSLILLLIALINSTTFVINGQMLENEAENIYISLTNLPWYSLNVQNRRVVYVMLMQSQKIIHMSASGLVSLNYQLTIVFFRCIYTGMTFLVNVGL

>TcasOR316

MTLMRKLQTAIRNLFEIQIKDDILAELLDWPTLVLFSKWPKNFAIFSTIYCVFDTLVCTLVYSTLDVEMLGKYAIFIAKSTIALCSFFSFFAKRKQYHKIINENFPHFWQLQSMGESTFDQMKKIATTVKFYSCLSVVAMLIGAVILILFTEDESEIYLSVKIYKDYVNKWTTGYIMFFYASFLYIGIVTAAVVFGLTYIVFHLIFQCFLLNQKLKLINSYIVKNGQKLVKLEERNQNFIYKELISCVKLHQRLIYFSNQINDLLYAPIFMYTFSGIVVGVALIYFLKTSIQYILTSLVLSIVSLIITTTFVINGQLLEDETENIIISLTNLPWYSLNVQNRRVVYVMLMQSQKIIHMSASGIVSLNYQLTIVLFRCIYTAMTFLVNMGL

>TcasOR322

MTFHWITTPLEPILKDDPLFVLMALPNKLIGSKLQALVNYFFFVYMVILPVSCFLVIVATNQWQIFYSPYSGYASGVFIVWSCYVSFFIFGSKYRRVYRDVFPHLWSLDVAGEEHHNRLKKIGKQLRTFKLVLITLAFIGATSGLPWFGDDYDFYIPIKLIVDYCDQWKLFFSIFFYLSFYHIGVTVLSCFFSLMFLVLHLQNQFYLLKTRLQTFATDSGTSDVFLSMKVKDEEYNRSVTQEIVFCIRHHQSVLMYCDRLNDLLYLPIFYFTLSFIVTGVSVILFPKYDLQALIRSLFVIVLGMCMTLLFCSLGQLIENESENVLYSLIEAPWYLWNTTNRRLYYLFLLKAQDTVNLSSSGLITINFQLILTLYRGIYSALTFFLNFS

>TcasOR328

MSYNIKLTKDDRLKLLKIMASDVFQSKTVKIILIVVFLVHAIANSLTIYFALHVSDTKQFISYASVFFSEFYPMLAILTIIFKGEVVQHLTDDINIWTIDGASKKLQSEIKLKIKILTAFVIINSFSVVIGGFCFVQQLSDDVNLFFAIRLIRDYFPNHSTILEFFYRMTYPICAYLMAVHAYQCLYYTQHINFQLQMFTEIITELTDLKTISLPENRLFYNKKYQTVIEQRLKFCIKRSQEFIKVCVTKNKEIGSLIPGFAICGLFLGIGITFFLSTGKFTTEYYLRMGVTSICGLTTFSALIWSAQTTETMINDLVMVINKVSWYNFNQSNKKLYLTFLLNTMKERKIKFTEKYSVNYQLGLAIVRGIYSVISVVASKRHH

>TcasOR329

MNCENQFAKDDYLKTLKIMASEVFQSKAVKVILIFVFLVHAIANLLTIYFVLYVSDTKLFVNYASVFFSEFYPMLAILTVIFKGQIVQHLTDEFKIWAIDSASKKLQSEIKLKIKIITAFVITNSLIAVWGGFLYVQPLSEDENLYFALSFIHQYFPNQSSTLEFFYRMTYPILGYLMTVHAYQCLYYTQHINFQLRMFTEVVAEFAPVKRFLLFEHHLFYNKKYQTEIEQRLKFCIKRSQEFVQICVIKNSEIGSFIPEFAICGLLFGIGVTFFLSTGKFTSEYYLRMGVTSFGGVMTFSALIWSGQTTETMTSELVKALNEVRWYNFNQSNKKLYLTLVMNIMKERKIKFTENYSMNYRLGLAIVRNIYSVISVVVSKRRH

>TcasOR330

MNYKKQFAKDDRLKTLKLMASDVFQSKTVKIILTVVFLVHFIANSLTIYFVLYVFETKLFINYASVFFSEFYPMLAILTIIFKGDVVQNLTDEITFWTIDSASKNLQHEIKLKIKFLTAFVIINSFTVVMGSFSYVQQLSDDVNLFLAIRLIRDYFPNYSTILEFFYRMTYPICGYLMAVHAYQCLYYTQHINFQLQMFTEVITELNNSKTSSLLENHLFYNRTYQTNTEQRLKFCIKRSQEFIKICVTKNKEIGSLIPGFAICGLFLGIGITFFLSTGTFTTEYYLRMGVTSICGATTFSALIWSAQTTETMTSDLVMVINEVNWYNFNQTNKKLYLTFLMNTMKERKIKFTENYSVNYQLGLAIVRGIYSVISVVASKRQH

>TcasOR331

MNFFQKKLAKGDFFKTLKFIASDVFQSKAVKMVLILLFLIHAIIYLLTIYFLLYVLEPKQFVNYATVFFAEFYPMLAILTVILKGKIIENLTDEIKIWAIENASKNLQSEINLKIKIITTFVIVNTLIAVSGGFLYMHPLPEDVNLFFALRLIRDYFPNHYTSLEFFYRMSFPIFAYLMTTHANQFLYYTQHINFQIKMFREVCLEVKAWKTVSPFENHLFYNKKYQTEIEQRLKFCIKRSQEFVKISVYKNKEIASFIPGFAICGLLLGVGLVFFLSNGKITWEYYLRMGFTSLGGVTTFLALVWTGQTTENITSDIERAINEIRWYNFNQSNKKMYLILVMNTMRERKIKFTEKYSVNYRLGLAIVRGIYSVISVVLSKYQH

>TcasOR332

MEFGNYKLMTDDYLKTIKFMSSDIFQPIPVKILLGFIFALHSAVNLVTAYYMLTTFDAKLFINYSSVFFGDFYPLLATFALISKNNTVRNLKDELEIWTIDSAGEKLRSEIKLKIKFLNIFVVCNSLLVLVTGLTFIQPLPKDSDIFFAYRLIHEHFPKHGQALEFLYRTTYVLISYIVAVQPFQIFYYCQHINFQLQISIETLKKISDWKTLSEDGENLIDNVKYQTEIKRRLKFCIQRSQNFICLHTEKIKEVSTFIAGFAVCACLLGIGVIFYLISGNFTPEYYVRMGFTSVVGIIIFAATIWAGQSTESAIDEMVTSLNEVEWYNFDQSNKKLYLIFLINSMRERTIKFTENYSFNYQLGLAIVRGIYSVISIVL

>TcasOR333

MEFEVKTFMTRDYLKVVKFLASDIFLAKPMKILLLLIFIVQASVQAMTGYFMATAFNAKFFNNYAPIFFGTFFPLLAISILLLKNKIFHNLKNELKIWSLDNAGEKIHSGITTEIKVVTYFVIVNSVFVLLANSTLAYPLSQDVNVFFGCYLIHKYILTYGRTFEFFYKATYLVIGHTNTGHVYQLLYYTQHINYQLQLYIEFIKFLDEGKTISKNEDDLFNNPTYQTLINQRLTFLIKRGQEIVKFHIKKTNEIRTLIPAFSVCTCTMGIGVVFFIISDNFIREYYFRMGMVSLVTVSTFAAGIWSGQSMETNLNEITTALNEVKWYNFNKSNRKLYLIFLTTSMRERKIKITENYSLNYQLGLTIVRGIYSVISVIINMK

>TcasOR334

MDHPDIKPMTDDSLKLIRFIASDILQPLPVKIFLGVIFLFFTVGSNLLMIYFVLYVYDIREFMDYAPVLFASFYSGVAILSAIFKGKIIHTLPDDISLWALDSGGEKIHSEIRFKARMVTIFVICNTLLIIGGIILNLIPLSDDLHVYFALRFIHEYFPNHKTCLIILLKASIFPVIPHMLVVHAYQILYYTQHSNFQIQLFNKVIAEVDFWETPLRETELFYSKPYQKGIEKKLKFCIQRLQVLINAYIVKTKEIGTLIALFAICGVLMGIGFSLYLFSGKFTPEYYLRLTFMTLVAVTTFSSIIWGGQSTETIITEMITALCQVRWYNFSQTNKKLYLILLTNMMKDRKIKFTENYSINYQLGLAIVRGIYSIMSVVVKMRS

>TcasOR335

MDHPDIKPMTDDPLKLIKFMASDILQPLPVKIILLVTLLALPVGSNVLMIYFVLYVIDIREFIDYAPVLFGGFYPSLAILIAVFKGKLIHNLQDEIKLWAIDSAGEKIHSKIKLKVRMVTIFAICNTLLLIVATVHNLIPLPRDLHIYFVLRLIYDYFPNHKTYLLILMKLMSPVTTYMLLVHAYQILYYTQHINIQIQLYNKFVADVDFWETPLCEPELFYNELYQKRVEKRLKFCIQRSQHFVYVHVAKIKEIGILIALFAVCGVLMGIGISFYLFSGNLTPEYYIRIFIIALVGATTFSSIIWGGQSTETIVTEMIATISQVRWYNFSQTNKKLYLILLTNMMKERKIKFTENYSINYQLGLAIVRGIYSVMSVLVKMYSINT

>DponOR24

MSVLGETTQIKKKWRSSIAITEKVLVITEIWPNDDTSLYRTMKVVFITIVCIVFNLTVIDELKMLAIRQDYKTLSMHLSTFGLYIGFSVKIILFQFTKHGPLKNMLDSMDSPIFHAYPPEMQKHQDNCIRVSNLIGKFFVYLVGGTILFYLNKPFYSSYPLPITFSHPLTTTTFYLLLTLQCVCSFYLIMIGICFDMLVMGLANVATAQLDMLIEEITTFTPTSIETLEKEEHRFIKRCAERHNAIISYVNSIEDVFTYIFLAQCVVSVTCICNGLFQLTHVAPVFSIHFYYNCIFTFNVLFEIGICCWFATLMTNKGNDVADACYNYNWLHSSSATRKLLLIMLCRSQKPLFITVGKIIQLSIGSFLSVLKTAYSYYALMQHLYDKTSQ

>DponOR25

MAIYPKCRLIQISMISSSLLGTFPWQFLFQDNKTFKNMYAMYSKLMLGHFTLFLFTAQLQLWILITDEELMRNAIFANLSVTFIYNITLAKQLIIMLNSNFRATIKQIIETENCKSPIEDDEVTNEIEFKMVQRSDKIVKCYGFLLIVLTILFFVKPFLMTPTIVSIGNTTKVIRDLPISSWLPFDEQEHYSYAYIWQVLNALQGSTYVASTDILMFNLIVFPAVQLRKLQHLLKNFAHYKEKVKTLYNIADDEQAAKITLVYFISRHMEIIQYVRRFNESMEIVMMFDFLQSSLHIASILPEVLMSEFSVMVVLMVASFLVSMIFRLILYYYHANNVMILSAELSYSMYESNWFDQTPKVQQMILIFMLRAQEPLTLRFGGFGVMSIESMIAILKATYSYVMLMI

>DponOR11Fix

MNLSKFIEFPRKTLVLTGSWPQQHSSWPYLSRRIIVMTSIALLLLALVYNASFHFDDPIKLSESLFILISVVNVFLKLIIMVVNEKVFLNLIARLETSTFIKGGILYQPIYVKFMEIVRPVYVTYFILVCGCVTFRSSFPVFSLSSVAYDNMAIDLLVVGVVSIAAVQLQVLNSKLRDTKQNVQFLPNYSISNHEALTVGYLKDCCIHYSDIEEYIKCLLDMFSIIILVQLGSSIVVICSSGMVLLSLKPLSIEAISLYFYLITMFTELGMYCWFGNFVYVESLEVINSCYLSHWEERGPAVRKTLFMLMERAKRPLEIKAVRFFTLSFDTFIVILKWSYSYFALLRNWM

AD

>DponOR16

MDFIQLFAPFKLILNISGFWPQKNPKPIVEVRKVFTLLVNLFFCLSLSIQCLFLRSQIEEFLDVLTVITPPVAYLFKQIVFFGHSGAFLTLMDFLKDDDLVSIPLQLRKQISDSLQVAKIIGVGYQACCTMTILFIVIWPMFTEHQLPVQFTLFDLGDFYAFMYLLQIFALANAAANSSSLDLIALTLMCIVKGQICVLNDKIRSLGEINASKGHGAQRVKYVSGCVLHHTKIIELVALIENVYSQIVLIEYLTSMVVICNIGFQLVIVELASFAFLLMLTFLVTMLCQLGMYCWFGNEIMLHSAAIRDACYESDWIHSCPQVRKMLLMIMERSKRPLYLTAGKFSILSLNSFTSVIHSAYSFFALMQRMYGKSTTF

>DponOR30Fix

MFIRNQRAFVARTCKLAGLYPVQLLPEDENLRKLYTIYYQALIMLYFICLISFCTELFHLLRAEKATVDDILKSISMTTLFAMTALRQWVIRSSPDVQKILRKAGNVEQRVYEENDPEVVNIFERAGHVALLYYIYYAVGTVFLCLGCILEPLYDNQKVFSGNATAFSRKLPLPLWFPYDIQAHYWETFCVTILLICLLVVFQVAVDVLFFYFIRSPVIQLEILHHFFKRFNDYTGRISVEPGNVASNVMMRKCIDMHRKVIKFVDIFNENFSNIIVLDFVQSSFRLASISAAIIMIESFTVTSFVFTLIFLWITLVREYYIYHAGNEIIFLSSGLVHSVYETDWYIENRQFKYMVKMFMVRAGKPLDIKIGRFGSLGFPALLSILQASYSYVTLVRGIQKS

>DponOR45

MAKTLSFIQLSKFYLLLSGLWPFKISDNFLVDKIYRIYTLCQICYYLCVIFGLLVNLVILIIRFDQPQRIIGDINLFIIVFECCLKVVIFQIGKVPFMLNQITQFEDSMEASGDAEVKDYYAKDAIYCRRINVIQSIATIIACASFAQDSVVTFITSDDMSVFKDNPFMHDLWYPFNREDYIYLVICIAFICDMQGLVCNAACQTTLLCLMIYARTRLKILQIRLRKFDKIAVEEYEGDVVRAVKDLIAEHQYLINFVKSLNDRTQHVLLLEFMLNSLCLASGTSQFIIIDTTSGWLATVFLNLYVIVQIFILSWHANEISVEGLAVSDAIAASQWQKQSKEVQKLLIIMMMRAQKPIGLTAGPFFRMTNSTAVQTMKVAYSYASIMTQNMPE

>DponOR7Fix

MANLKQALQIYDICSFLEGEHIRLGIGGFYPRRIKRTFIVNLVTVFAYIITIAQMAVVINFVLSITDIVTITEVLLFSMTQVGFVNKLVNFHRNSRKVATLDELISQEIFTRVTVAEMDIMKTSFQRCQKVLNIFLLSCFGVTLLYGVVPAVNGIMTGTKMYPFPGKFPFNPDDYFVLIYGGEVATVAVSAWNNGAMDCLFTKHTVIATTLFRILRKKIKDLHYNTNEGERPLENRIKHCVRYYNEIIKYVSAIENIFAYGILVQFMCSAIVICLTGFQLLVVASESGQSGLLVVYLFCMMFQLVLYCWYGHMLMEESNRITEACYAINWHEMKIGQQKMLITIMERAKKPIALKALGIFRLNLSTLMTILRSSYSYFAVLQQIYRNDKIMALVTN

>DponOR19

MYPIRKDLPFYASLRMLESIGFYSENTNGFKKRSIVRTLIFCILCWSIILLSAVLLIYENLNDKNYASVFFNIAVAVASTSTYCCTLLFVKYQEKWSDILTALVNYEKFGKPRRYNQLKERGDRVAMACWGGILTGVFLYMLFAILHENDCELEKTGGGVCGLIIPTWLPAPYDNSLLARRLVLLYDIPNAAAVSSFVLVTHLNIQVNEFNIARIDHLSLLFNDIEFCKDPQAQLNKMKHCIEYHQDIIRVSLQFKNLSKRTMGHMTLTFTIVTASMGCQLLQTSKNFYQENAFFFEIIYVINMFIMCYCGQRLEYKMKTVGDFLYSTHWYNLNPKLQSLIPLVILNSQKTIRMDAVPIGYLNYELFVTLLKTTFSYFSVLTQLT

>DponOR43

KFFLKLSGLWPFKISDNVLVDKIYRFYTLCQICYYLCVILGLSINLVILIIRFDEPQRIIRDINLFIIAFEICLKVVIFQFRNVPHMLYQITGYEDTIEGSSDAEVKAYYAKDAIYCRRINVFQFIATFLACASFAQDSVVIFLTSDDMSVFKETPFMHDLWYPFNRADYIYLVICIAFICDTQGLICNTASQTTLLCVMIYARTRLKILQIRLRKFDKIAVEEYEGDVVRAVKDLIAEHQYLINFVKSLNDRTQHVLLLEFMLNSLCLASGTSQFIIIDTTSGWLATVFLNLYVIVQIFILSWHANEISVEGLAVSDAIAASQWQKQSKEVQKLLIIMMMRAQKPIGLTAGPFFRMTNSTAVQTMKVAYSYASIMTQNMPE

>DponOR40

MYSKQESLLGMLKPMMMFTGTWRLDGMNSTVRWFYWLYSLIFHGFGVLFIISVVAKFVEFVKSGADSEDIISSQVFLLSGTCIFSKFLIYQICNVSDILKAILQEEEKIWLSKDSESISAYQADIKHVRKWNWGILLSTMFTGVALMSAGVASLIQADISSINSEGNEKEEWSMIPMWLPYNEREHRSTVVVLKCIFTFIYVCMFIVSGMTFVALMIYSLGLLKMEQVKIGKCNWTSYNMADLSVDMKTLLINNRRVFRFIKHLDRSIRYVVLVDVLLNSISIAALATNITNVQRGDFVFCTGFLLMQVTQVFVLGWFANDIIMLSRTRADVLYNLNWYYLDLKNRKLFGMMLMQCQQLSVISIGPFGPMTIGSVISVIKAAYSYMMLMQSYK

>DponOR6

MIKQPPYEVYATDFFSVNRWILKCAGLWPPSTPNRVVRRLYQLYTIGVFLFVNLWFTGTEFVSLFYTYKSQYELIKNVNFFLTHFMGAVKVILWYFYGHLLRDIMNALESPQLHYEGYADFSPHRISHLHRAIGRRYSLLFLCLAHATLISSYIPPLIAVAEYLNQPQGGLQKLPSRLPYFCWMPFSYDTPGKYLLAVAYQAGPMFSYAYSVVGMDALFMNILNCIAENMVLIQGAFKTVRERSTLHYCVGALAPPCDAIREHPLVLRQMDLETKKIIKHLQITLRACKHLEGIYHIITLSQVTATLFILCTSLYLISTASPFSKQFFAELVYMMAMLFELFLYCWFGNEVTLKYEQLPMHIWESQWLATDDCFKKQMIFTMLRTNRPVYFTAGKLARLTLPTFMSILKTSYSIFALIKNFSK

>DponOR14

MLDISVSAVSGNCVIFAYTLVILLQCVIEVTGQLYLLTTKPSIGKILLLAPMFFTSFMVITTIIALLFQRSSHGRIYSQYEALSMEQLRRANKPILAEVRQLFDAGIYQFAAFLAAVALGSIGYLPLNEFDYEHNVWPAIQALKLIEDGPKAVIYTIATLNYLVMPGKGCILLLYGLHIMHLCSLYCVSSILLRGKLKGIAVNRPAAADLMLSDQAWVTQELKSCIKQDVRLKTFCSAIIDHFKWILLFHVALAVIILSLLYYINLFLNGGYSPLGSRCFVAIANILSFTYSYNSENFREQVENIRQEICNLPWYSFNKCNQKLVHVFLSNMLQPRYLSVGGLFDANHEFLVLVMHKVFNVLTILSNLDPDRAGN

>DponOR33Fix

MKNDFFGFCIPLARFIYIMPDKTPQNVYGWRNKLWAVFMYGLAVFCHLTEIIKLFQIVTAKYFLLGEFIRNFVITSLHFTSLGKAMFIGGKTGKKAFEKILDFEKHVYKNLGDDIRLIYKNKVTSIQKVKKYYLIGIILVVIFYVAAPIFREPIHIQDGNQTIRFRQVPLSSWSPFEQYYWLTFIWTGLTGIYLSIFFVTTDLICYSYVQMINEGMKNLMVLDFLPGSVQLAGMIYQMMTNLSVIQCILLGQFICSLIARVFIYSNSANNLSQLSKQLAVDWFEIDWTELPKDVTNNLNFCIMRSQKNLQITVGDLSVITMESFLTILKGTYSYLMLLMTI

>DponOR39

MSDSKSKKVFAVMRIILMCAGFWNQSISKNRLINKIFHGYSVFVKLSCLVFWLLILAETSRLIICQYEITIITASVAILLTDTKIVVKIVIFLKHNILDIVADVIENKDEIRTEHYKEIKMLYDRKANFLKFAISVLGGSTTGAVFLLQGSGGALVLQDRKHNKLFNDTVETHGMYQTIFPLHRNNHIYWLFATEVFWSYLGITANIVTQLICVIILLHAASRLEVLQVRFKHLIMPDFQIKASDEDMKAKVTELKSIIRKYQLTIRFINEFNQSTKYITMIEFCLSTFDMASCCASLTKMKGYESVWLLFFMMVLLTQLYLIGWTANEIRVQSEAIATALYESNWYELNKEGRQLILISMIRAQRPLNINIGPMGPMTTRSILTVLKGAYSYINIMR

>DponOR3

MMENKSYRIMQLHTNLLKCLFLWPVDTFSPGLNSLLMYGSFCISMCCGIPIISAAGYQFYVGIEDVNILLEALIGVYDIIGNTVTYVCFLRKQRQIQEIIDDIKDFFQYCGYETVRKIDSEIMCHTKYFLFYVTVSVILNLAWPMLSVNNCLKSRRSDFYIKHDPCGMPTQNLYPFEASEGIVFWVLYIIEAIFCYHTCCFFSLATVIVIGFLKHITAQLKCCAYKFEHICDYMGSCKNDENVIIREFVHLIKYHQRIIKYAEKVFGIFDVMIIVYIGVTSFTLAIIGYQIAIPKTNLEDRIRYTMLLIGWVLLFYSICFYGQQVRDESMKVGEAIYKSEWYQHQTLLGMKTDIMFVMRRTQKPLDFKATLLGEVSLIVFVAVMKRAYQLFTLLLTVTEDGP

>DponOR5

MDTLGPALTKVRLRLPSLDIPDSKGTDYPKNLYSAIDRISFLCGQAKLSKNRSWILRFAYSLYSYTLIIIAIMFIISEIITFRKSLTELTTVLSEIGMMFTHLVGMVKFWILIHKRDEIEQVKNKLRDVQFEYVGIDDFQPGLKMRKEKLFIIIISTFIFALYNFVGISAHISAASMMYKYTANGNFLGNTTCETFVPYYFYYPFDVSSPSSCHYLLFYMDLSLDIYASYIATFDSVFVILLNLLATQLNILGDALRTIRKRCVKRLQMKVDSSSLYDADNPLLENEMYNELTHCTKHLYLLLEVGNDIESIFTFLTLLQTIASLLIFASCLFVAARVKPTTPIFYSQLEYFSAVLSQLTVYCWFGNEITLASSAIPYSIYSSDWFSSSERFKKSMLLTMARLQRPLYVSIGKFTPLALTTLLSVIKGSFSYFTLFQSAGTD

>DponOR36

MKPIEDQSLFRACKILVLCGGMWRGNIPNWPIAHQKLYKVFLRGAQFAYFFCLPSLVLSLWVNVDQDNEKAISVLKNITFVVVIFCKMIIIQSRPVTMLIEAASEKEQQAILSEDPQISEIHRRHVVYTEFVVKSIMLCTFLAGLAYVVGDLYLANEFYKLHPNAAPTDPKPHSIYFWFPFNPDEYYKIALTYEFVHIVQTVIYNGASHAVVNSAIIFVKVELKILEYEIRHMMSKPNLSNLTPAQLMKIHIRKHQELIKWVCKFNDSFKYIILLEYSVVSLTLASTLTEILQGIKMVFNGIFFLLSTLSLFILSWNANEIIVTSVFDLSDALYHFPWYELDKEAQELVLFMMLRCKRSLNISNGPFGFLTLRGAVSRLKLAYSVVSVLSR

>DponOR37

MFSASKWVIMSSGSWGLEVDSKYRILYKIYVLYIRFIYITSTVAVFAMFLVNLGSNNDKAIEALSLTLCSVSCIIRLAVCLKQKVVNLLKIVMEDQFNYAVNDPKIKVMLQEYKSYVTFLCVFVVCYTYSLVILFNIFNGIIEFQSFRKLHPNATEYPQYLVSIWLPFNVQTHFTLALICQTVLFLQSCVLNYSSTVLFNTLMIYVVIKLKILQHLFQNFNTYPKNLENFHMELRDVLAIDNLKHLIRQHQDIISFVKELDKNIKIGVLIEYTITSLMLATISIQVLTGNKVASFSFYGLILIYQLFLLSWNAAEIKTQSEKIAGAIYATDWYVYGPGVKQIIHFIIMRCSKGLSLDIGPFGPNDLGAASARLKLAYSYVSVMGNNK

>DponOR27

LLLCVIQPYNCLKMAIRTSLFQFAKYFMTFCGLWKVPFSPKVQRFYVVFSNVSHFVYCSFVLSLFVKALLIVVGFESSDNVFNAISVAVIMFDINFKAMIYVKFGLPRLFHQLMKEEQSIEENSHQEISEYYLRQCELYVNICTLQLVTTIFPTYFYILVNILQFPLDAENFMYEMWIPFPVQWKVLAIFKIVICQYGIFMNTAVRSALQSLMMFITSQLWILQVNIRNVFSEFSEEAAREELGKLIRKHQFLIGFVEKVNDSVKYILLLEYLLDSINMAAAMLQIATASSVTEMTFTLVYFILLLTQLVILAWSANEINTQSVEVSNAIYQSNWMDQ

>DponOR12

MEKDKFKILSLHINVLIFLQFWPNPLFNNVVNNHIMSIVCFITVTSCIPCIWTIYKVFEGMYDIGILFESFICFVNIMAYLTAYWTIFRNKAVIENLINDICIFLPYCPTNLIRDTDASSIRYTKYLIVYVTLGVFVNLAWPAISPEGCMRQRQSEYLLKHDPCGMPTHNYYPFDASKPIPFWIAFACEALLTCNICILFSMVTAIILGLLMQITEQIKHCCDKFEHINFKGDVETARKEFLECVRYHRAILEYAERVFTVFAPVMSAYLVVTSFATALIGYQIVETDNTQDRFRYAMLLLAWGCLFFMICLYAQILQDESVLIADALYNSDWTCNSIYFRHYIIRVIARAHKPLYFNISFLGKISLTRFVSVMKTAYTVFTVLVTVVDRK

>DponOR32

MNSKVNQAEKLDPVDKRSKVFPSSDFLRLYAIFTGQMGIFPWQLMFERNKTYQNLYNLYSKLILSYYMYVTVSMWLALVFLCLEDTLRIPEITKNITVSVICTVTIIRLFVMKLHPAFLRNITFIIDAEQYILSSNDAEVHRIYKNCKIISNRHTIFFIVLSYLMALFISLRPFFTDAYEINYKNESLQITSLPLSIWVPLNEQEHFLSVYFWNVLNNLVMTSIVLSIDIITFLLLIYPVGQLQILHHILSKFENYKNRMKLNYPGLDDDTIGAITLKACIDLHRNIIAYVDDLNACMNIFMVVDFAQSSLLLTSVFAQLLWVEPSITFYGFVFMYVTYLNQRLFMNYYYSNEVWLLSENLNLSVWKSNWYEQSHYVKFMIYFFIMRTRKSLKFKIGPFGFMNLSTYIAILKASYSYIALLHSTQK

>DponOR15Fix

MGGSGEFSSNILNLGISALHLCATNRVSRWFLIKNEFEVILANLRKINTDFSLFDYQSHQSGFKMDANEPEIHNEEYIGQMAWRNLNCTQATGFKLRYADDTASSAISNAIIKYKTTKLLETKRYCLGIFLTFLINVALNISISYTINYGNPLYEKWNPLLNKTSVYRDYPYPLLYPFDTSVSDGHYLLGFFYQPYAFFCLMCAFFCIEYLCVGTIIHLTTHVNILGYAFSYVDENIDPMLDYSKVIMLKEKRIIKLSGELKEIYNCAKELNAVFSGQLLMQEFLMSTVMCCCVYRVTTNISTAEIGYLSTMVAVCVAEMFTVSWFNQCFTLELFKIQQRIYELEWIDYPPKLRRVLLFLMCRVQKPFNFTMGFGFPLDVNVFLSMIKTSYSFYTLITRSGSKFSNEDV

>ItypOR28Fix

MGLYPASRYFKNPIMWSSILGAFPWQMIFQENAKLQQVYRWYSNFMLTWYFGMVTTEYIQLYHILNANVIQMDEVCENVCMSLVFTCTGLRVWVMRRTNGLSEIIQTVVDAEREADGLDDEKTRQYEDIHVKHMEKVSFIYAAFVFMSVTNGCLATLYADTKSVIIGNSTIVEKPLIISTWFPFDKNEHYWVAYGLQVFDGYMAALTVACTDILMFNMISYPIGQLTKLQHLVRNMAVYKTHFEAFPTFTKIVQRHKHIIKYVELFNQSMGTFAIFEFVQSSVQIASVLVQTSPDDLTLMSFCFIVLFFTSMLTRLFMYYYSANEVIIQSINLGDSVWESSWYHQPHQLKQAMLMVLVRAQKPVSYKIGGFGIMSMQSIVAILKATYTYISVILRN

>ItypOR32Fix

RVKQLRLVSILCGLIGILPYKFISPDKKLYQDIYRAWSILSNLIFVLGMFLAYMKLYTLFNEEKIRFVELSRNLAVTMLCTMTMARQIIIRVKPEVSNMLSQILETEEAILIKNDKTVAEIYTKNANNLYRKSVYFWFIMFNVVVSHIARPFYIFEEIQQGNVTVIHKTLTLSLWFPVDDQEYFWECYTISSLYAVCFASFQSYTDIFMYAMITYPVGQLQILFHTIKHFQTYKMELQRLLDSMNDSIAAKLMMKNCVEMHRLIIKYIEDYNSCMSILTVFDFFQTSIQITSILSQIVMIEITVFLAVFIMIFLTMVFYRLLMLYYYANEIIITSEDLCQAVWQSEWYNEPPHCKFMMQIMMMRSKTPLMLKIGPFGSMSLRAFLSILQASYSYFMLVYSQKDN

>ItypOR31Fix

MHIFPKSDHLDFSFALCSMLGILPWKLVFQDNSFLQTLYYLYSKTLLIITVIFITTEWMEVCRILNXDPVNLTDLNNAIAPVLLFTVTAIRMIIFNRNPDFMKLLNYIINRQEFMAQRDDEIRKPSQKFIKINKWTGVGYLIMYLAVIYQLLALPLVLGPIEQQTANQTTTIRILPLLSWIPFDTQQHYWGCYLWQALNLQLASCNICHIDVLMFALILYPIEELGYIKHVLRNFDSFKSRTGIENSNFASITVFKDVIKIHNNVINYVGTVNDTLSFVMLLDFLQSSLHIAVILGAVVVGGPTDLASLSFVGTHFFSMVLRPFLYYYYANQVMVLGGDLTKEVWNVDWFDESKDVKYMVRFFNMRAQKPLQYFVGSFEVMNLQSFISILRVAYSYVMLLHTLQ

>ItypOR23Fix

MAVYPKSEYLKVPAIYCSTIGIFPWKFMFQDNKNLQTIYRCYSIVMLAWCIGFVVTDYIQLVILLTSKTLDMQEISFNTCITLLFTCIGLRAVIVYFSPNSANLIQSIIDSEKVTYLDDAECMKLEKKHLRSVRLISHCYFIFIIFSTTSRCVYSFPKEPDIIQNGNETEIVKEHMLSIWFPFNQEKYYLTVYNIELLDSFLGTFFVAYVDIYTFNMISYPKGQLKKLQHIMKHFHNYKAKYSSETNEENDFIVFKDLVQRHKQIIQHINAFNELMEFVAIFEFVQSSAQIACGLTQSSLENLTIGSFLFVMSFLISMLVRLFLYYYAANDVTVESTKLAQCIWESNWYEESQKIKLSMLMVIIRAQKPLIFKIGGFGAMSVQSIVTILKATYSYITLAYKRT

>ItypOR16Fix

MYNIPENERKNYFLKFSRVTMLMLGIWPVRRGGDLLEKLYESYFLTTFLYYIAFNLSGLALAIRTWSNNYLTTASSMGIVIEYMSNAYKVWLFKTSVFKSLIKEIQDREREIFEGPDEAFKEIYIRNAESNKKVVLFYTIMGTSGISLYFITPLVSNVLMPLGYNNVTGVYEHYFIVFNWFPFDPNRYYWAAYLIQFTGCLIGYSYIVHCGAFYISILNFIRTQLKILRHVIVNMSEYSLLYKNTYKLTEEQSQFVLLRAVVLEHQRIISFVTKTNHTIQLFTLINFVISSFQLALLVYQIFQVAILQQVTVLSYFITLSTQLFLTYYAAHMILFESSNIASSIFEGNWDTYPPQTLKLLQMICMRAQKPLAMTIGPMAAVKVTALFQIFKALYSYICLIIKF

>ItypOR34Fix

MKFRELIHNDFLGICIQLGYYFCIIPEKAVTTDKIESRNYFFYTCIVRTLILYCHICQWVKMYQIITADIFIFDELVRNCAITSIHFQSFVKTSIFRQNYQLFENVIDFENVLYKNNDQKVLLIYRDTLQAIKNSRLVYVFGILIVIVFYIAAPLFRGPYYVEMGNETVTIIQLPLSAWSPTNNYFSNFAVTGAMGAYLAMVFVQTDLLYYCFLYFSICQLNILEHYIVHFHHYSNELVNDHKCSHVMALSLTQKIYIKYHQNIIKNVKQLNDALKNSLLIDLVPSSIQFANQFYIIATNLNIMQCVCIGFFTIMLMSRVMAYCYLANQISVQSQKIGSAWFQMDWSDFPNEMKKMISFCIMRAQKPLVITLGNFGNITLMTFVGILQASYSYVMLFITL

>ItypOR2Fix

MKVLQRETEITFFKFNIWVLKTCLLWPEDLNYKYDKKRFIKDLTMVASLMPCFLPILADFLQQLYEEVPDLTEAVENMIALNCLIGMFYMVICFVRNRRMIIQLMIDIRTFNKYGNDSITQEVDNKANLFSKMFMFYGILGNFVYMAMPQIRVSKCHLNRTEDMIEKGVPCGLVVRSYFPFKFDYSPVFEIVFVHQIYTCTMVSVVVLVLTMLFCGFLMHIVNQLKHLRVLIARLKNVPPEKFERKLIFVVRYHVAIIQYSQNTAGAFSTMLLFYITLTSVVLSVLCFEILMVDAFADSVRFTLHLLGWLIILLSICYNAQLVLDQSQEVANDVYSLDWVSILSVDVQKKSKXVIMRSQKALVMEAGGMGVVSLSAFLKVLSSAYSFFTLLLKFK

>ItypOR8

MSELFLLHFPRILMVICGVWRLPYFKSKKVQTVYDIFSIFLQFTFSLMCLSMFFELVNLINTWNVLNLIEFSRVALSSYLCIIKALVLRNSSIQRIMVYMIKEERNVLRSKHQQPKALYMDTVKLINRVSFLLLLVVLPTLLAFSADCLRKGIIDFDDAVKYVYLPLIDQKKYKTVQLTVQTIFINLIGFYYCMTQAFMVTAMKFAQGQLELLQLYFREFDYYAARQSTTEIAYLKTLLEYHQKIIHFVETLNKEMRLVIIIEFFFSAVNIACSLFTLLTMATNLIDILFSVNCLVFLLAQLAILSYLGNEIYQAGLNIASASYELKWYEKNREFQKNLLLVIKRSQKPLVLSVGPLGPLTNETFVSVLKASYSYFNLMTRYN

>ItypOR17

MGLMTTPRFFLQLWGIWPVNTLLPPKYVMYRFCIIGWYSFFNIFQFIASIRLILNNESFERISRCISVMVTLVLMLVNSLIYQKNCIPQLCSTVMEIEQXLARSNDAKITQTYHTTVAKNKYLNLYIVGSSLFTLVAFIGLSLLDVIKAGPAFWDFDNVTFMHELYVPFNRGNHQALIITTNIFTACESVVVNGVIQTTFYALVMYGALRFKILQLNLKKIEQTEGDRKWRMRELIQDHQYCIRFVGELNQATKNVLLMSFVLNSLKVASVLFPLMAIREFTDLAFPLIYSSMLVSEVVFQGWMCNEITEQSLQVAQTIYDTFWYKESKQYNVLLQLMLMRAQRPVTMRIGPFGAMTTSTILTTMRAAYSYATLMMNSS

>ItypOR18

MYTLSKNKPFYYALVLLRAFFWYPSSPKCSVTFILCSLVLRLLSTLAALGTLAHLVLNLTGETKAEISEDIGDLTGFVGCMSACLNFLWHRSRWSSFINRLLTFKQFGTPPGYVKVVRRGNLITLACILYTIPGMLWYSHLTHLDIPRCEALNREFGMKEACGMVNPTWIPPGYDRRNGWRFWVLYVLQCTGIFVYLPSTFVISNIPLEAVGVIVTRIHLKYHLKRCGSDLGRLYHCVKYHQDIIEVSKELSDLVQATLGTLLLTGAVVIGSLGSQVIKASTPKAVTFILGYVTTIFMVCHAGQKLINQSLTLADQVYWMEWYEQQPKIRKDLRFVLARCQKPLGLVGPPSMGXAGYSLFLIMLKTSYSYLTLLNEVIS

>ItypOR4Fix

MDKQSQILKFHVKVLKFLMIWPFDGLDPNQNYYLMRGCFAYACFCSIPVFSGAAFQFCVGIDNVKVLLEVLVGVGNITGYNIAYVCFLKNQEKIQFLIRDFQEFVQFSGPEIIQNTEEKTTRYTKYLLGYASIGLVITFSWQMLSTESCVAQRGGDYYVRHDPCWLPVRNWYPFDASQPKLFWIVFPIEAIYSIHICLFFSLATSTIIGFLMQITSQLQYCSNRFEHVFDEVDLKPFQQIKPDFLFLIKYHKKILDYSKKLFNVFDALIVVYISLTSFIMAIICYQIVDPKISAQDRIKYAILLIAWCLLVYLICYYGQKVQDEALKIGQSIFKSHWYGGTTAVELKPYILFTLARTQIPLEFKAQLFGTISLLQFMKVMKWSYSGLTLLLAVTDED

>ItypOR13Fix

MSNYQRYNIQTFLKEERLFLGITGFVSGNLKLHLPTGVTYVLTTMQVLASIYYGLSTTDLAEITAAFMITLSHINTLNKLFGLHLKSLTQLDRILVKQIFALADDRELTILKRTLTACHNMLTMYLVTVLGSILLYGATPLVANYTTMERNYPTLAKFPFNPDDYYWAVFAGEFFIVALSALSNGCMDRLFAKHVAIATGLLKILRHKIKQIMDVDDQKVIEAKMKHCVLYYNEVMGYANQIENQFSFGIFIQFLCSCIVICLIEFQVLLATSSETIGLLLTYLTCMITQVTIYCWYGHQLMEESNSISMEFYDLNWIEMSVKNRKTMLTSKERAKYPIVLKASGVFPLNLATLMKILRTSYSYFAVLHQVYTK

>ItypOR9

MFSSKNLYKYYSHFASTSIIMYTIMLTIRLVQLVIEGQTPSAKLYRCFTINIVIYMMTANLIIFRRYGLPDLISQVMKDEEAALNSLDKDIRKTYLAQTKIYEFTSVAQVVSTFASGLMFIALNVYMKVKGLLKHEAFMYELWFPFNRENHDGFVIFFNLYIVVLIMFCNVASRIIPQTMIIYANAQLRVLQILLEKAFDAPCSDPLVKIQELVKKHQDLINFITFLNSALRNVIFMEYIINAINVAAGLLQFITVRAAMDLVYAFVHFSLLVIQIFVLALNANNVSTQSEAIANAAYNSQWMDQSNNIKKIIYIMIMRAQKPLVLNIGAFGVMNAESALTTMKAAYTYV

SIGLQR

>ItypOR19Fix

MLIFGNIISANVIVLFGSSLINGDYFLVTSSFPFALAIIVVNGSTLSFAVNHKQWSNLFKSLTDCQKFGKPPNYDLLKKNGDRKGMICMTCYISTCTLFAIIEAVEEQRCLKNVSKHEICGFIIPVWWPTNYHPSTFLKTMVQVYEVGTIIILSNFTIIATLQYQVCEYIAAKAAHLGLNFNAIDPNSDAKTQFEQFKLFVDYHQHIISLCAEFDSSCKRTVGHVTFTTAAISALFSYHGMQGNYKLLAFLALYILNLGFMCHTGQNLEDAMLGISNSIYSSKWYELNIRVRQRIPFLLARTQKRIGLDAVPIGYLNYALFMTVLKTTCTYLNLLNHTI

>ItypOR27Fix

MLNGDVLKMDAICSNICLTLAFTCSALRATVMRVGPNLLKIIEQVMHAEKNPASIEDQTSFNLERKSIKTMRKLSHLYAVAITMIASSKCALAPFEKGEIVHIGNTTIIDRPLIMSAWVPFNKNTHYWAAYIIQIYFAALGAWHVAYVDMFMFNMLGYPIGQLKKLHYYIKNITTLTRNDDSLEEFKNVIRQHQQIISYVKFYNDSMGTFAIFEFLQSSVQIASIFIQTSPSDMNLGQFGFIGGFFIGMLFRLFLYYYTANEVMTESEKVGVSVWESDWYEQPTNLKMALLTVMMRGQRPLYYKIGGFGLMSVQSIVAILKATYTYLTVVVRNN

>ItypOR43Fix

MDAALALMKNITFVAVVVFKTVVVQSDAIVKLVKAASVEEEKIRNLTDQAIRKIYKSNVDYCNRVTKVIITYLYGSGTIYVLDGLYKSYTYYENHPNVKPEDPKPHTVLFWFPFDHNRYYKIAIAYESFHIFQTLNYNGVAQSVVSSVMVFLKIELKVLQHHIRAIQGGSRDYQKVLIKCAIKHQQIIQWVNDFNNNFRFIILFEYSMISLTLATILIDILQGTKICFNATFFALNFTQLFVLAWNANQISDESSISISDALYACSWYEFDKTTQDFVLFMTLRCKKPLNISNGPFGYINMDAALSRVKLAYTVVSVLSTSTK

>ItypOR36Fix

MKIPHMFEHAMKHERNIFKSRDLELIICYQEQVKYGRRVNLSQFLVTTLSTSTFAVTALLDVYWAADMSKYEKEPFMHDLWFPFRRETHMNWVIFFNLFMIVQGTCFNTATQATLINLMIYSSSRLKLLGLKLRKFDAIASQNGRDILETVHDLIFEHQDLLSFVESLNVRIKYVLLMEFILNELGLASGIIQLIVIDTTSYMVSVVTIIILQLFQIFVIAWTANEITIQGAKIADSVMASNWVEQPTNIKKLFLIMVMRAQRPLGLTAGPFFNMNANTAVSTVKAAYTYLTFMMNNYN

>ItypOR6Fix

RYATDFFFGKPMDVKKGWFTEALKQKQGSKYSLMFLMLAHATLTSSYVFSTITTIQHMKGNSTVALPDRLPYYSWMPFSYDTGPKYLLAIGRIKQVPMFSYAYSIVGMDSLFMNIMNCIAANVTIIQGAFKTIRERALPGQPNNVPHESKADMDVLRVELRKIVNHLQTIFKACDKLENVHRMVTLCQVTATLFILCTCLYLVSIAPPLSKQFLVEFVYMLAMSFQLYLYCWFGNEVTIKFQELPRYIWASSWLATDTQFKKALLFTIMRTKRPVFLTAGKFSRLILPTFMSILKTSYSIFALIRNTSK

>ItypGR6

MSYNGDVIKYPREITVFAAVCAVVFCVLGIVGNSITILALFRCPKLKSHATTAFVISLCVSDLLFCGFNLPLTAARYIAEEWIFGDTLCQLFPVFFYGNVALSLLNMVAITLNRYVLITLYDYYTKFYSKLSICLQLFCTWLIAFLIMMPPLTGIWGQLGLDPSTFSCTILEKDGRSPKKTLFLFGIGFPCLVIILAYSCIYWTVRNSKLRLRSHEPLPNQRTSKRDRDDRRLTRLMALIFICFVLCFLPLMLVNVFDDKVSYPTLHVLASIMAWASSVINPFIYAATNKQYRSAYKRLLALVRSSIGPESMHSNSLRSKQEKFQGVSYKAK

>ItypGR4

MQFVNIVELNALEFHLEGVLLNLIFLYMAKIWAAFVREWSLIEKSMKYYEGPKNAKIKIVVAMVSIMGIALFEHALVTSQIAWTSIRNNPLSFFNASREYFVHHEFVEVFHFVPYSIWTGLFFKLLIIQKTFIWSFIDVFISTVSICFHCKMVQISRKVARLSAYEEKNCMIWRSTREDYTKLSKLCQIVNSKLRWLIILSFFNNVYHILSQLFNSLKPNDDAMHKIYFCISFTLLILRMTTVCVYAGSICDEQDRLITVLTTAPSGVYNIEVERFIMHLDTFEMALSGSNFFKITKGLLLKISSAIVTLRISVNSV

>ItypGR3Fix

MVETVHPITKVAPAPPPRYSNGDLMEAFDDSGELNSRIFSCLKPAYATLRLFGLMPVTQSGPVFHVTAKWIIYSFMLLCSLAGFLGYLKYFNIAITRNAEGRFEEAVIDYLFTVYLLPVALNVIAMYEASKQAGVLTQIVAFERIYTRTFRKRLTLDMGSKQLILISVLLILGCVVMVVTHFTMANFIVYQVVPYCYVNIVTYIIGGSWYIYCDVIGKVATSIAEEFQFALKNAEHSSRVADYRSLWMMLSKIIRNVGNSFGYQLTFLCLYLFFVITLTVYGLLSQIQEGMGIKDIGLTITGVSAVTMLYLICDEAHYASSCVRTYFQKKILLMELSLLNEEAQQEINMFLRATEMNPTDMCLCGFFDVNRNLFKSLLATMVTYLVVLLQFQISIPAGNDNLTNSTLNS

>DponGr1Fix

MSKPPMAAQMDGQFLSPYPPEGPDVSRRKSDNIRIVTPETVRPEHVPDNELLEKLHTYDNFYQTTKSLLILFQIMGVMPIQRLRGKTIFRWFSANTCWAYFVYTVETIFVSIVFKERLVLILKPGKRFDEYIYGVIFLSILIPHFLLPLGAWRNGQEVARFKNMWTKFQLKYFKMTGTVIKFHRLTLTTYSLCVLSWLVGILIMLAQYYLQPDMLLWHTFGYYHILAMLNCLCTLWYINCTAKGRVAGWIAEKLQEALQTKGSAKKLLTIGKLWVDLSHMMQQLGTAYSGMYALYCLLVLLTTI

>DponGr3Fix

AYFQLFAVVQFSLFLQKWIIYSFLLLLAVAGFIGYLKYYNINVTRTAEGRFEEAVIDYLFTVYLVPIFVNILALYEAKKQANVLTQMVSFERIYTRMFKKRIGFDLGSKPLVMTVVLLILGCGVMVITHFSMANFIIYQVVPYCYINVITFIIGGAWYIYCDIIGNIATCLAEEFQFALRNIEYSNRVCDYRSLWMILSKIIRNVGNSFGYTLTFLCLYLFFVITLTVYGLLSQIQEGMGVKDIGLTITGVSATLMLYFICDEAHYASTCVRTYFQKKILLVELSFMNEDAQQEISMFLRATEMNPTDMCLCGFFDVNRNLFKSLLATMVTYLVVLLQFQISIPSSGDDVTNNTTNTTPSAPK
