# Supplementary material for: Antennal Transcriptome Analysis of Odorant Reception Genes in the Red Turpentine Beetle (RTB), Dendroctonus valens
Source: PLoS One. 2015 May 4;10(5):e0125159. doi: 10.1371/journal.pone.0125159 (PMC4418697; doi:10.1371/journal.pone.0125159)
Supplement: S5 Fig — (DOCX) [file pone.0125159.s005.docx]

**S5 Fig. Amino acid sequences of SNMPs used in phylogenetic analyses**

>DvalSNMP1

MSQHQTPLFKGMVNLVLRDSPVFLPIVSKAIPSIFDNPQTIFLTAKVKDILFDGVELNCSGKEFGTTAVCSQMKSQIPGLKFKKDNENIFLFSLLGSRNGTLTRRLKVHRGITHAKDLGRLVELDGKKEINIWREAECNRFHGTDGWIFPALTTPEEGLQSFSTDLCRSITLQYINDTVLKKVPVRVYETDLGDQMTDENEKCYCRSANSCLKKGVFDLTKCMGVPIYATLPHFLRTDPSYINLVDGLAPSELLHAIRVYFEPMTGTPLFAAKRMQFNLDLQPTNKIPLFSHLPIALFPMFWLEESVDLDGYLLKKVQ

>DvalSNMP1a

MPKLKTLSFVFFPKLHLLALFTKFAYRCGKKQSPEKNMNFPMRLAIGSACSVVFIILVGFVGFPKMIKGKVKDMVNLKPGMEIREMFLKVPFPLSFNVYIFSVLNPAEVQGGAKPHLKEMGPFCYNEWKTKINVADNEGDDTISYEPVDTFKNAKRPKCLSVDTQVTIPHPMILGMVNTILRQKPGALTLANKAIKSIWSNPSSLFITVKAQDLLFNGVVIHCGVSDFAGKAICTNLKAEPSLTHLGGDDLGFSLMGPKNGTAGKRIKAFRGTQDFHKVGRIIEFEGKPKLDVWNNSKCDAIVGTDGTIFPPMLKKEEGLASFAPDLCRSLIAQFEKHDKYDGIPVSSYFATLGDQSRNPTEKCFCTTPETCLKKGLMDLYKCAKIPLYVSLPHFYDSHESYLKGVKGLKPDVEKHGIRIMFESLTGSPVSARKRLQFNMPLEPNPKVELFHNFTPTVLPIFWVEEGVDLNNTFTKPLKTLFLTKKLVNVVKYLVLMMSIGGFCAAGYLYFKSDNSMNVTSVQKVQPDQNGHRNIISTVFNGNHTAGQDNEAYEDKH-

>DvalSNMP2

MKVPFPFQFKVYLFIVENAEEIQAGVKPLVKEQGPYVYQLTRWKDQVAWNHSTDEISYHEYEAYKFDAESSGGLSEQDLVTVLNPAFLSFLYTAEMDPATRDFLPLIDESLDAIFGSYNSPFFTNITVREFLFDGLRICKNGCNDDGFVAKMACNKIKERMAVTKQMRADGTDILYASFHYRNNSHQGYITVAAGQKNETTIGEITQLNHQSTLNVWTKDQFECNRVSGLTTVFPTNVGVDTTFQSFSEDICRTVSFEYSRREMVGLIKGNRYEALKSTFNTSKNACFCTDRTRNFDGNVGCLYNGVLDLTTCQGAPVLVSFPHLLYADSRYLDKVEGLNPDPSKHAMFVTLEPTSGTPLKVAKRVQFNLLLRAVRNITSLETVGNSIVPMFWIEESTALPEKYQDFIKNKIYRTLLIMDIVRYTVLALALAVIVCCIVLFIYAT

>DvalSNMP3

LLLHAVDIMQYLMIIIGCGCVTIAMYFRLKNRKSVTITPATGTRKSAPPKPNEKMDVSNLSIAGILGDRAEKKP

>DponSNMP2

MFRNCCSPRLVFLYNLLAVLLLIASLVLAFWGLPQIISKQIHKQTELTENTDQWDRFKELPFPMEFNIRFFLVTNPADVLNGSMPILKESEPYKYKSTIKRTDIRFDDIEEDSVTYRRSFSFEFDGSGTTREDDSITVINPLLMASFQLTNDIQRLAMAGCRKYILEPAGLDQVFLTTTVRKLLFDGIYFGFQNATGKGVACEMVRKELGKIVANVRVVEHLNDTDCYRLAIFNYKTDNFLKNSPDGIYTINRGRNNATALGSIMRWNGATTSTTYGTSTSINNLTCHSIKGTDSTIYSPELKAGENLMIFNTDLCRTIQLVQVSSNEVFNGINAFRYSTGYTLFRPETILKENDCYCSHGTKGADGKPSCFLDGLLDFRPCLGAPVLISQPHFLHADVKYIRAVSGLSPDEDKHDIYLLLEPNTGTPLEGRKRVQMNSVLRRQPLLSMITPPNMYEAVVPLLWLDEGFTLPQKYLDDLNAKYFKTVRIATGFKFGFIAVALALLVGCLFVACRKMYFRNAK

>DponSNMP1a

MNFPMRLAIGSACSLLFIILVGFVGFPKMIKGKVKDMVNLKPGMEIREMFVKVPFPLSFNVYIFSVLNPAEVQGGAKPHLKEMGPFCYNEWKTKINVEDNEGDDTISYDPVDTFENAKRPKCLSVDTLVTIPHPMILGMVNTILRQKPGALTLANKAIKSIWSNPSSLFITVKAQDLLFDGVVIHCGVSDFAGKAICTNLKAEPSLTHLGEDDLGFSLMGPKNGTAGKRIKAFRGTQDFHKVGRIIEFDGKSKLDVWNNSKCDTIVGTDGTIFPPMLKKEEGLASFAPDLCRSLIAQFDKHDKYDGIPVSSFFASLGDQSKNPAEKCFCTTPETCLKRGLMDLYRCAKIPLYVSLPHFYDSHESYLKGVKGLKPDVEKHGIRIMFELLTGSPLSARKRLQFNMPLEPNPKVELFHNFTPTVLPIFWVEEAVDLNSTFTKPLKTLFLTKKLVNIVKYLVLLMSIGGFCAAVYLYFKSDDSMNVTSVQKVQPDQNGHRNIISTVFNGNHTAGQDNEAYEDKY

>ItypSNMP1Fix

MPHPKNIAWAGGALAFGGVLFKVWLFDVLVRFGVKDQTALRYRNEVRGIYLKIPFPLNFKIYFFNVTNPEEIQNGAKPVLNEVGPYWYDEYKERVDVIDNDTEDSLTYTPYDLFKFNPNMSTPLSDNDYVTIIHPVIVGMVNLLLRDSPMLLKVVSKAIPFIFNDPKTIFLTGRVKDILFDGVVLNCTSKEFASTAVCGQMKGQVPGLKPTPGQPNLLLFSLLGPRNATRTGSLKVLRGIKHFQDLGRLLEVNGRKSIGIWAGDQCNRYDGTDSWIFPPLIQPESGLKSFSTDLCRNIKMKLVNETVVKKIPVGVFEPTWGVKVVTRRKSATVPTLPVXXXXXVFDLTKCMGVPLYATLPHFLDTDPNYLKLVDGLKPDHEKHRIVVFFETMTGTPLKAAKRMQFNLELQQTNKLELFSKLPAALFPIFWLEEGMELEGYFLKKIQTVFMLLLFADVTIYVTIATGLSVCGAGFYQYWKNTKSLSITPLTKNNNGLSEPKLN

>ItypSNMP1aFix

DLFKCAGVPLYASAPHFYDCHVSYLKGVRGLHPDEQKHAIKILFESLTGSPVYAKKRLQFNMPLEPNQKIDMFKNITPTVLPLFWIEEGVKLNNTYTKPLKSLFMMKKIVGVVKYLILLGSIAGVTVGVYLYFKSGDTVNVQ

>ItypSNMP2Fix

MRFLQRVKFNLKTVFLCGISGVSLLVVALFLGFIIFPKVVNDQLLETKILREDTEQWAIFKKIPFAFTFNVYLFTVENPEEILKGAKPVVKEKGPYVYKLYKWKEDIIWNYTTDEISYYEYEKYVFDQEASGSLTEHDKVTLLNLPYLTFLYTAEANEATSGFLPLIDEALEFIFSGHNSPFLVNVTVRDYLFEGVEICKNGCEDDGFVAKMACGKIKDNLKVAKQMRLHHKDILFATFHYRNNTHQKYLTVNSGRQNHLEIGAITQLDNSSTMNVWNQFGCNQVSGLTGIFPINLGFKT
TFQSFSAEICRPVKLHFSTIKPFGSIKGYKYVALNTTFNTSMVENQCYCTGKIPNLDGNLGCLYDGVLDLSTCLGAPIVVSFPHFLYADWRYVNNVKGLSPNETNHQIFVNLEPISGTPLEAATRIQFNLFLRPVRNITSLDSVADALVPLFWIEELTYLPQKYQDVITGKLYRSIFILNAIKYVLLAIALVIITVCILIFLYTD

>DmelSNMP1

MQVPRVKLLMGSGAMFVFAIIYGWVIFPKILKFMISKQVTLKPGSDVRELWSNTPFPLHFYIYVFNVTNPDEVSEGAKPRLQEVGPFVFDEWKDKYDLEDDVVEDTVSFTMRNTFIFNPKESLPLTGEEEIILPHPIMLPGGISVQREKAAMMELVSKGLSIVFPDAKAFLKAKFMDLFFRGINVDCSSEEFSAKALCTVFYTGEIKQAKQVNQTHFLFSFMGQANHSDSGRFTVCRGVKNNKKLGKVVKFADEPEQDIWPDGECNTFVGTDSTVFAPGLKKEDGLWAFTPDLCRSLGAYYQHKSSYHGMPSMRYTLDLGDIRADEKLHCFCEDPEDLDTCPPKGTMNLAACVGGPLMASMPHFYLGDPKLVADVDGLNPNEKDHAVYIDFELMSGTPFQAAKRLQFNLDMEPVEGIEPMKNLPKLILPMFWVEEGVQLNKTYTNLVKYTLFLGLKINSVLRWSLITFSLVGLMFSAYLFYHKSDSLDINSILKDNNKVDDVASTKEPLPSANPKQSSTVHPVQLPNTLIPGTNPATNPATHHKMEHRERY

>DmelSNMP2

MIHWSLIVSALGVCVAVLGGYCGWILFPNMVHKKVEQSVVIQDGSEQFKRFVNLPQPLNFKVYIFNVTNSDRIQQGAIPIVEEIGPYVYKQFRQKKVKHFSRDGSKISYVQNVHFDFDAVASAPYTQDDRIVALNMHMNAFLQVFEREITDIFQGFANRLNSRLNQTPGVRVLKRLMERIRGKRKSVLQISENDPGLALLLVHLNANLKAVFNDPRSMFVSTSVREYLFDGVRFCINPQGIAKAICNQIKESGSKTIREKSDGSLAFSFFGHKNGSGHEVYEVHTGKGDPMRVLEIQKLDDSHNLQVWLNASSEGETSVCNQINGTDASAYPPFRQRGDSMYIFSADICRSVQLFYQTDIQYQGIPGYRYSIGENFINDIGPEHDNECFCVDKLANVIKRKNGCLYAGALDLTTCLDAPVILTLPHMLGASNEYRKMIRGLKPDAKKHQTFVDVQSLTGTPLQGGKRVQFNMFLKSINRIGITENLPTVLMPAIWVEEGIQLNGEMVAFFKKKLINTLKTLNIVHWATLCGGIGVAVACLIYYIYQRGRVVEPPVK

>TcasSNMP2

MGCSCCTIKVLLVCVVISVALLIVSLALAFKVFPDLLESEVNKAVRLEDGTKQYDRFVELPFPVDFKVYLFNVSNPQQVLDGTEKPKLEEIGPFVYKQYRKKTILGKNEEEDTISYTQKETFEFDAEASKPLTEESVVTVLNPALMSIYQLAEDLHLAGAADTCIKQTFENNQGKVFIEANVRKLLFDGFSFCKNTSPGICGLVNDLICAIAATKRNSDLVLPDYSLIFSYLNYKRKPDDGKYTVKRGLTNIEKLGHIVAWNDSLYTKFWGEGTTCSEVKGTDSTLYPPRVTTDSAFYIYSTDICRFVKINYKGEESYKGIDGYLFETSEDTLRSSAPEEDCYCSKLSRDMEGKKSCFLDGVIDMQTCFGVPVLFSFPHFLWADNKYLSAVEGLNPVEEKHKTYLVVEPNTGTPLKGMKRIQLNGVIRPIVGIKSMLQTKRALLPLLWIEEGVSLPQKYVDELKSSYFDKVQIVDGVRYALIVISAILVGAFGIIILRKRSHAKHHV

>TcasSNMP1a

MRLPVKIAIGCAIGLVVIIVFGFIAFPKMIKGKVKSMINLNKGSEIRQMFVKVPFALDFKIYMFNVTNPMDVQKGALPVLKEVGPFCFEEWKEKVDLDDNDDEDVMFYNPKDTFYKANGPGCLDGSQMITMAHPLILGMVNTVVRTKPGAISLISKAINSIYGNPDSIFMTASAMDILFDGVVIKCGVKDFAGKAVCSQLKEAPDLRHVDENDLAFSFIGPKNATPGKRFKVLRGVKESHDVGRILEYDNKKEMEVWPTKECNQYKGTDGTVFPPYLTKEEGLASYAPDLCRSLVAVYSGDTKYDGIPVRIYTATLGDMSKNADEKCYCPTPDTCLKKGMMDLFKCAGVPVYVSLPHFYESDESYVKGVVGLNPNKKDHGIQILFESTTGGPVKAAKRLQFNMPLEPNPKLPIFANLPNTVLPLFWVEEGVALNNTFTKPLKDLFKIMKIVKIAKWLIMLGCLGGLGAAGYLYFSKKGEANITPVHKVKPAENGVSTLGGEVNHAMSDNEIEKY

>TcasSNMP1b

MVKWQRQLKPGNEVRDFYIKLPIPLDFRVYFFNISNPEEVKQGEKPILKQIGPYCYDAYKEKINVEDDKDNDTLTYNPYDTYFFNQMRTGDLSQDDYVTILHPLTVGIVNAVATQKPQYLSAVNKALPVIFKENSSIYLTAKVREILFDGVLINCNVKDFSANAVCSQFKGQPAMVEVEKNIYSFSLLGSRNGSIPTRITIHRGVKNAADIGRVVTIDNKTDLDVWPEPECNAFRGTDGWVFPSFLEKEDGIWTVASDLCRSFKAQYVEDLKFHGVVVRKYFADLGDMSSNPAEKCFCPAPEKCLPKGVMDLTKCMKVPLYCTLPHFLRADEKLLQQVEGLSPELERHIIKIYFEPLTGTPMLGQRRIQFNLQLMPIPKVAMMKTVPEALHPILWIEEGVELEGFLLKKVTSVFTLLKLMTFVRYIMLGLSIQGILYGGYKLYQESKSKKVSPVQNGTTESKNHNQGKTGGIELPSMNKRNKENTKNA

>TcasSNMP1c

MSYKKITIISACCVVTIIGVAYIYAIRDISHRRNVRYKYIDRVNNVSNDVNGGVVSVGYCYDYKRIDVDNADSTYTYDIYNRSGNSDDYVTIIHVVSVNYVSVKTHYNDAGKSITAKVRDIDGMINCTSRDTAMAVCTIRTKIGISKDYKYAGNGTTRITVRGIKSNGKVAVDNVTKSDWSNCNYKGTDGWISGRKTIWMHATTCNIHADVGATSNGAVNKYYSDNICTNCSCGIDVTKCTAIYISHRSDSIRGVKGNDTSHITRIGTSMAIRNVVKKITIMNVSVIHVWVMGVVNGWRMIKTYTAVMKYISVASGTAYGGYHYKNKKYSKNIVSSK

>TcasSNMP1

MTSTARRRNIMKKVYKIMDRVYNITNSVNGVVKVGYCYDAKKIDVNGDSTYTYTYNDKSGRTADDYVTVHIVGIVNTVSRDSIVDRAIKSIKDNIYITTKVRDDGMTINCKVDSATAVCTKAIGIIKNVYKSIGRNGTNRYKVRGMKKWHGRVVNHKSTVWSTKKCNRRGTDGWIIDKVGWTYSSDCRNMHVVTSHGVAKYYADGDMSSNDKCYCKTCKGMMDTRCMGVIYATHRVDKVRRTVRGKITDHIVRVIIGTAKRMNIVKKISMKTAHIWIAIVGKMIKVVVAKVDVVKYCAVCAVAGSYCYKRKKKAVTVSKTAKA

>AplaSNMP1

MYMKTPFALDFKVYMFNITNAEAVLNGESPVLDEIGPYCYDLWKEKVDPIDNEVNDTLTYKGKMTWIFNKAKSAPLTGDEMVTIPHPLILGIAVAVARDKPAMLSLVSKALNSIFNNPPSPFITATTNEILFEGLTVYCNVTDFAGKAACAQIKSEAKNVIYISDKIFKLSFFGDKNGTVDERPFTVKRGLKNYKDIGRVVEFDNKPNMNVWPTKECNEYHGTDSTIFPPLLQKEEGIVAFSPDICRSLAAVFEKETFVKEVKVNKYTATLGDMSADDSLKCYCPEPNKCLKKGLMEITKCVGAPLYASLPHFYASDESYVHGVRGLHPNEEEHGIYMYFEPMTGTPLGARKRLQFSMPLEPIPKISFMKNLPTTILPVFWVEEGADLGDEYVDQIKSAFKMIQIVFTAKWILFVVSAVTGAIAGLMHFKNSKNILVTPVKNDESKGRSSVINTIDSLNTYVNGSNNKY
