# Supplementary material for: Antennal Transcriptome Analysis of Odorant Reception Genes in the Red Turpentine Beetle (RTB), Dendroctonus valens
Source: PLoS One. 2015 May 4;10(5):e0125159. doi: 10.1371/journal.pone.0125159 (PMC4418697; doi:10.1371/journal.pone.0125159)
Supplement: S1 Table — (DOCX) [file pone.0125159.s006.docx]

**Table S1. Gene ontology of *D. valens* antennal transcriptome**

| **Ontology Class Number** | | | **Total** |
| --- | --- | --- | --- |
| biological_process | biological regulation | 2674 | 38416 |
| biological_process | cell killing | 1 |  |
| biological_process | cellular component organization or biogenesis | 1987 |  |
| biological_process | cellular process | 5854 |  |
| biological_process | developmental process | 2336 |  |
| biological_process | establishment of localization | 1555 |  |
| biological_process | growth | 390 |  |
| biological_process | immune system process | 313 |  |
| biological_process | localization | 1825 |  |
| biological_process | locomotion | 560 |  |
| biological_process | metabolic process | 4417 |  |
| biological_process | multi-organism process | 294 |  |
| biological_process | multicellular organismal process | 2460 |  |
| biological_process | negative regulation of biological process | 726 |  |
| biological_process | positive regulation of biological process | 589 |  |
| biological_process | regulation of biological process | 2438 |  |
| biological_process | reproduction | 787 |  |
| biological_process | reproductive process | 741 |  |
| biological_process | response to stimulus | 2165 |  |
| biological_process | rhythmic process | 89 |  |
| biological_process | signaling | 1511 |  |
| biological_process | single-organism process | 4478 |  |
| cellular_component | cell junction | 198 | 20508 |
| cellular_component | cell part | 4330 |  |
| cellular_component | extracellular matrix | 55 |  |
| cellular_component | extracellular matrix part | 24 |  |
| cellular_component | extracellular region | 254 |  |
| cellular_component | extracellular region part | 144 |  |
| cellular_component | macromolecular complex | 1874 |  |
| cellular_component | membrane | 1922 |  |
| cellular_component | membrane part | 1325 |  |
| cellular_component | membrane-enclosed lumen | 579 |  |
| cellular_component | nucleoid | 7 |  |
| cellular_component | organelle | 3085 |  |
| cellular_component | organelle part | 1916 |  |
| cellular_component | synapse | 272 |  |
| cellular_component | synapse part | 191 |  |
| cellular_component | virion | 1 |  |
| cellular_component | virion part | 1 |  |
| molecular_function | binding | 4527 | 11016 |
| molecular_function | catalytic activity | 4207 |  |
| molecular_function | channel regulator activity | 7 |  |
| molecular_function | electron carrier activity | 47 |  |
| molecular_function | enzyme regulator activity | 302 |  |
| molecular_function | metallochaperone activity | 1 |  |
| molecular_function | molecular transducer activity | 282 |  |
| molecular_function | morphogen activity | 3 |  |
| molecular_function | nucleic acid binding transcription factor activity | 205 |  |
| molecular_function | protein binding transcription factor activity | 92 |  |
| molecular_function | protein tag | 1 |  |
| molecular_function | receptor activity | 217 |  |
| molecular_function | structural molecule activity | 351 |  |
| molecular_function | translation regulator activity | 7 |  |
| molecular_function | transporter activity | 733 |  |
| Total |  |  | 69940 |
