# Supplementary material for: Antennal Transcriptome Analysis of Odorant Reception Genes in the Red Turpentine Beetle (RTB), Dendroctonus valens
Source: PLoS One. 2015 May 4;10(5):e0125159. doi: 10.1371/journal.pone.0125159 (PMC4418697; doi:10.1371/journal.pone.0125159)
Supplement: S2 Table — (DOCX) [file pone.0125159.s007.docx]

**S2 Table. Semiochemicals overlap within three bark beetle species.**

| Source | Compound names | *Dendroctonus valnes* | | *Dendroctonus ponderosae* | | *Ips typographus* | |
| --- | --- | --- | --- | --- | --- | --- | --- |
|  |  | Phsiology ※ | Behavior ◎ | Phsiology | Behavior | Phsiology | Behavior |
| Host | (-)-α-Pinene | + | + | + | + | + | + |
|  | 3-Carene | + | + | + | ? | + |  |
|  | β-Pinene | + | + | + |  | + |  |
|  | Limonene | + | + | + | ? | + |  |
|  | Myrcene | + | + | + | + | + |  |
| Beetle | (+)-Ipsdienol | + | + | + |  | + | + |
|  | (±)-Ipsenol | + | + | + |  | + | + |
|  | cis -Verbenol | + |  | + | + | + | + |
|  | exo-Brevicomin | + | - | +/- | + | + | + |
|  | Frontalin | + | +/- | + | + | - |  |
|  | Lanerione |  | + | + |  |  |  |
|  | Myrtenol | + | + | + |  | - |  |
|  | trans -Verbenol | + | +/- | + | + | + | ? |
|  | Verbenone | + | -/+ |  | + | + | + |
| Non-host  (NHV) | 1-Hexanol | + | - | + | + | + | + |
|  | 1-Octen-3-ol | + | - | + | + | + | + |
|  | 3-Octanol | + | - | - | - | + | + |
|  | Benzyl alcohol | + | - | + | + |  |  |
|  | Hexanal | + | - | + | + | - | - |
|  | Z 2-Hexenol | + | - | + | + | + | + |
|  | Z 3-Hexenol | + | - | + | + | + | + |

(※) Active in electoantennogram (EAG) assays or gas chromatography- electroantennographic detection （GC-EAD）or in single-sensillum recording (SSR) antennal response

(◎) Active in lab and field bioassays, as attractant or anti-attract alone or as synergist.
